# Complexation of chiral amines by resorcin[4]arene sulfonic acids in polar media – circular dichroism and diffusion studies of chirality transfer and solvent dependence

**DOI:** 10.3762/bjoc.15.187

**Published:** 2019-08-12

**Authors:** Bartosz Setner, Agnieszka Szumna

**Affiliations:** 1 Institute of Organic Chemistry, Polish Academy of Sciences, Kasprzaka 44/52, 01-224 Warsaw, Poland

**Keywords:** cavitands, chirality, macrocycle, resorcin[4]arene, self-assembly, supramolecular chemistry

## Abstract

Directional self‐assembly of conformationally well-defined complexes in polar environment is still a major challenge in supramolecular chemistry. In the present study we demonstrate that resorcin[4]arene sulfonic acid (RSA) interacts with chiral amines (amino acid derivatives and aminocavitands) to form inclusion complexes and capsules based on electrostatic interactions. The complexes were characterized by circular dichroism and DOSY NMR spectroscopy. Chirality transfer from amines onto a resorcinarene skeleton was manifested by the appearance of signals in CD spectra and diastereotopic splitting in NMR spectra. The complexes proved to be thermodynamically stable in methanol, but DMSO and methanol/water mixtures were found to be highly disintegrative for these complexes. This result is quite non-intuitive and worth attention in the context of formation of supramolecular complexes in polar environment, for which DMSO is most often a first-choice solvent.

## Introduction

Despite of the great progress that has been made in supramolecular chemistry in polar and aqueous media, formation of discrete ordered supramolecular associates with precise patterns of noncovalent interactions still presents a challenge [1–2]. Especially self-assembly involving chiral molecules has elevated requirements for precision due to the necessity of distinction between compounds differing only by handedness. Such strict requirements are difficult to meet, because hydrophobic forces that are the strongest interactions in aqueous and polar environments are nondirectional, while directional interactions (charged/neutral hydrogen bonds) are diminished by solvent competition. Numerous studies show that the most promising strategy for the formation of ordered complexes in polar media involves a combination of at least two types of interactions, one of which is usually a hydrophobic effect, while the other one has a more directional character, or application of multidentate interaction sites [3]. Macrocyclic compounds with persistent hydrophobic cavities constitute a fundamental class of scaffolds for the construction of supramolecular host–guest complexes in water [4–5]. Their cavities provide initial spatial constraints and prevent self-aggregation while hydrophobic interactions ensure thermodynamic gain. However, these restrictions alone are usually not sufficient to ensure the formation of complexes of a precise geometry. Seminal examples here are cyclodextrines, which, when unmodified, interact with a wide range of hydrophobic guests albeit with low general selectivity and enantioselectivity in particular [6].

**Figure 1:**
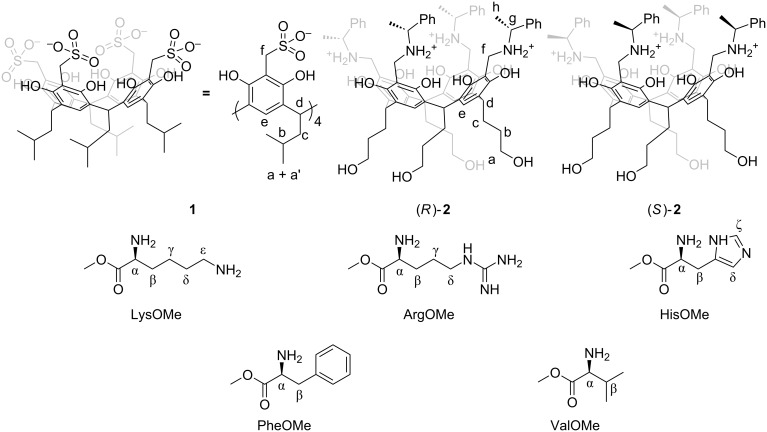
Structures of the compounds used in this study and labelling scheme for NMR spectra.

In this paper we present a group of synthetic macrocyclic compounds – resorcin[4]arene sulfonic acids (RSAs) and analyze their interactions with chiral multidentate amino compounds (Figure 1) in polar media (DMSO, methanol, aqueous media). RSA **1** features a hydrophobic cavity and a polar rim of sulfonic acid groups capable of forming electrostatic interactions. We will put a particular emphasis on ordering during complex formation that is manifested in chirality transfer. We will also present a crucial and non-intuitive solvent dependence.

RSAs (e.g., **1**) can be considered as analogues of calix[4]arene sulfonic acids (CSAs) – the class of macrocycles widely studied in the context of various host–guest interactions, especially with various amines [7–9]. Recently the group of Crowley showed that CSAs also co-crystalize with proteins modulating surface interactions mainly through interactions with surface-exposed lysines and arginines [10–14]. RSAs, in analogy to CSAs contain hydrophobic cavities and sulfonic acid groups capable of forming electrostatic interactions. However, in CSAs polar groups are positioned directly on a macrocyclic scaffold, while for RSAs at conformationally labile methylene linkers. Therefore, we think that RSAs are more adaptable than CSAs and are able to enclose guest molecules more tightly (by an induced fit mechanism) and, as a consequence, they can form better defined complexes. We think that RSAs can serve similar purposes as CSAs and their adaptability features and possibilities of additional modifications of their lower rims can be highly beneficial for applications in modulation of protein crystallizations. Due to the interest in interactions with proteins we have selected basic amino acids as chiral bidentate amines to interact with RSAs. We have also studied interactions of RSAs with chiral tetradentate ligands (tetraaminocavitands) in order to evaluate the influence of a chelate effect on stability and ordering, and, additionally we aimed to test the possibility of formation of water-soluble capsules, which are of particular interest to supramolecular chemists.

## Results and Discussion

RSAs can be readily synthesized by the Mannich reaction of upper-rim unsubstituted resorcin[4]arenes with sodium sulfite and formaldehyde according to a general synthetic procedure [15]. While the synthetic procedure for water soluble RSAs is straightforward, purification of the final products is tricky and highly substrate-dependent. RSA **1** was obtained in 92% yield after purification involving column anion exchange using DOWEX^®^ 50W resin. Chiral resorcinarenes (*R*)-**2** and (*S*)-**2** were synthesized by the Mannich reaction of *C*-(3-hydroxypropyl)resorcin[4]arene with (*R*)- or (*S*)-2-phenylethylamine and formaldehyde in methanol in 38% yield for (*R*)-**2** and 36% yield for (*S*)-**2** yields according to modified procedure described in the literature [16].

Complexes of **1** with various amino acid methyl esters (LysOMe·2HCl, ArgOMe·2HCl, HisOMe·2HCl) and with (*R* or *S*)-**2**·4HCl were obtained by dissolution of the components in water (or in water with sodium acetate buffer, pH 7.0) and mixing the solutions in 1:1 (for complexation between **1** and (*R* or *S*)-**2**) or 1:5 stoichiometric ratio (for complexation between **1** and amino acids methyl esters). In all cases except monodentate amines (PheOMe, ValOMe) we observed precipitation of the complexes. Samples [**1**(PheOMe)_2_] and [**1**(ValOMe)_2_] were obtained by mixing of **1** with PheOMe∙HCl and ValOMe∙HCl at pH 7 and lyophilization. The samples were further analyzed in methanol, DMSO and methanol/water mixture. We would like to stress that upon dissolution of the precipitated complexes they can either remain intact or partially or entirely dissociate depending on the solvent and concentration, therefore, we introduce a notation that distinguishes a structurally integral complex from its solution that may contain various components. For example, the intact 1:1 complex between **1** and (*R*)-**2** is denoted as **1**(*R*)-**2**, while its solution in methanol is named [**1**(*R*)-**2**)]_MeOH_.

### Interactions of RSA **1** with methyl esters of amino acids

**Figure 2:**
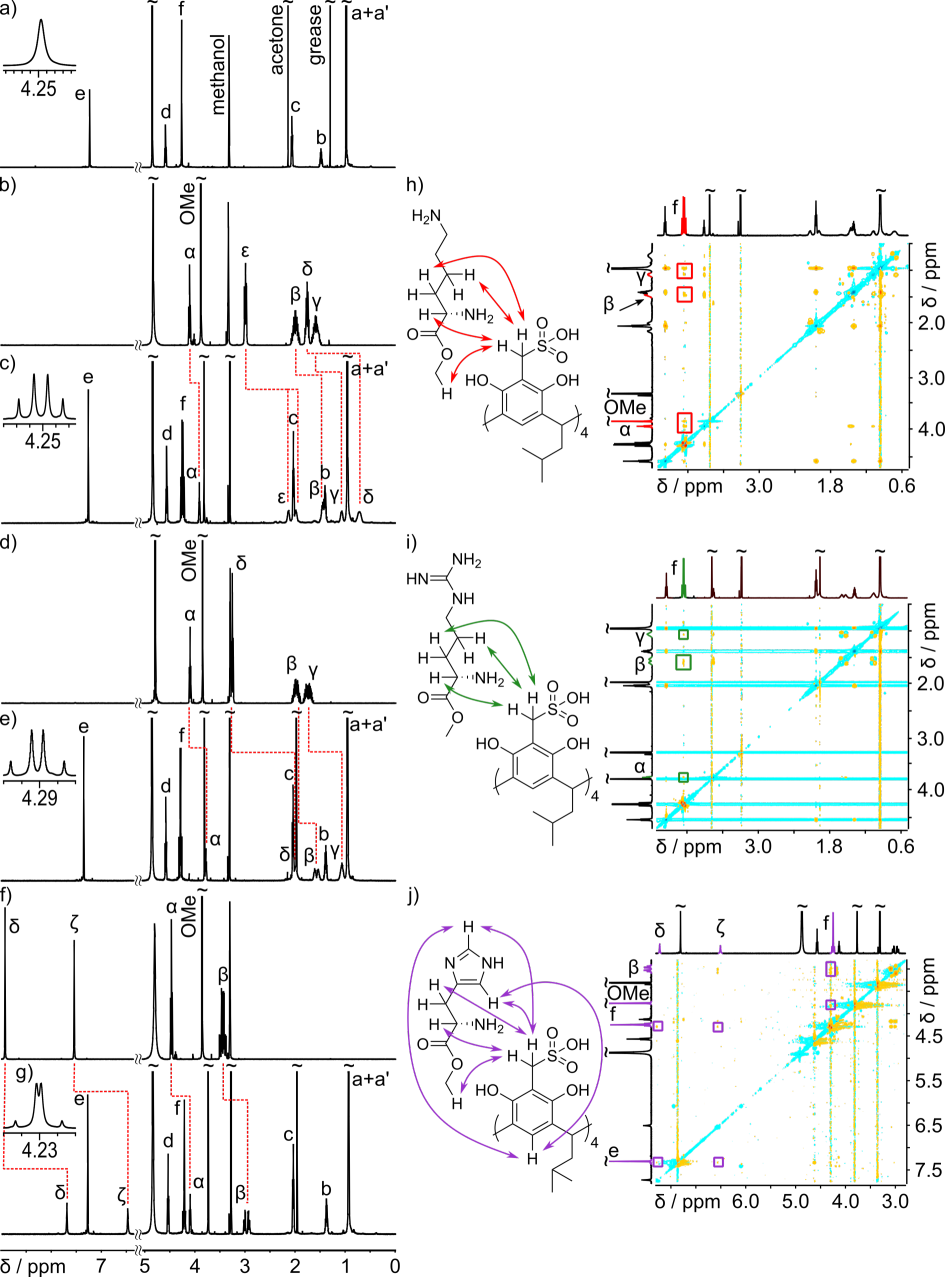
Spectra of complexes [**1**(LysOMe)_2_], [**1**(ArgOMe)_2_], [**1**(HisOMe)_2_]: ^1^H NMR (a–g) and ROESY (h–j) in methanol at 298 K, 600 MHz (NMR).

The complexes of **1** with LysOMe, ArgOMe, and HisOMe precipitate from water solutions at pH 7 (sodium acetate buffer). The complexes are soluble in methanol and DMSO. The composition of the precipitated complexes was determined by integration of signals in ^1^H NMR spectra in methanol-*d*_4_. All complexes have 1:2 stoichiometry, consistent with formation of neutral salts, i.e., **1**(LysOMe)_2_, **1**(ArgOMe)_2_ and **1**(HisOMe)_2_. The comparison of NMR spectra of the complexes with the spectra of the substrates reveals considerable complexation induced shifts, mainly for the guest molecules (Figure 2a–g). For **1**(LysOMe)_2_ the most upfield shifted signal is for the δ-CH_2_ group, that may indicate incorporation of this hydrophobic fragment in the aromatic cavity. ROESY spectra for all complexes show correlation signals between β-CH_2_ groups of the guest and C*H*_2_SO_3_^−^ of **1** (Figure 2h–j).

**Figure 3:**
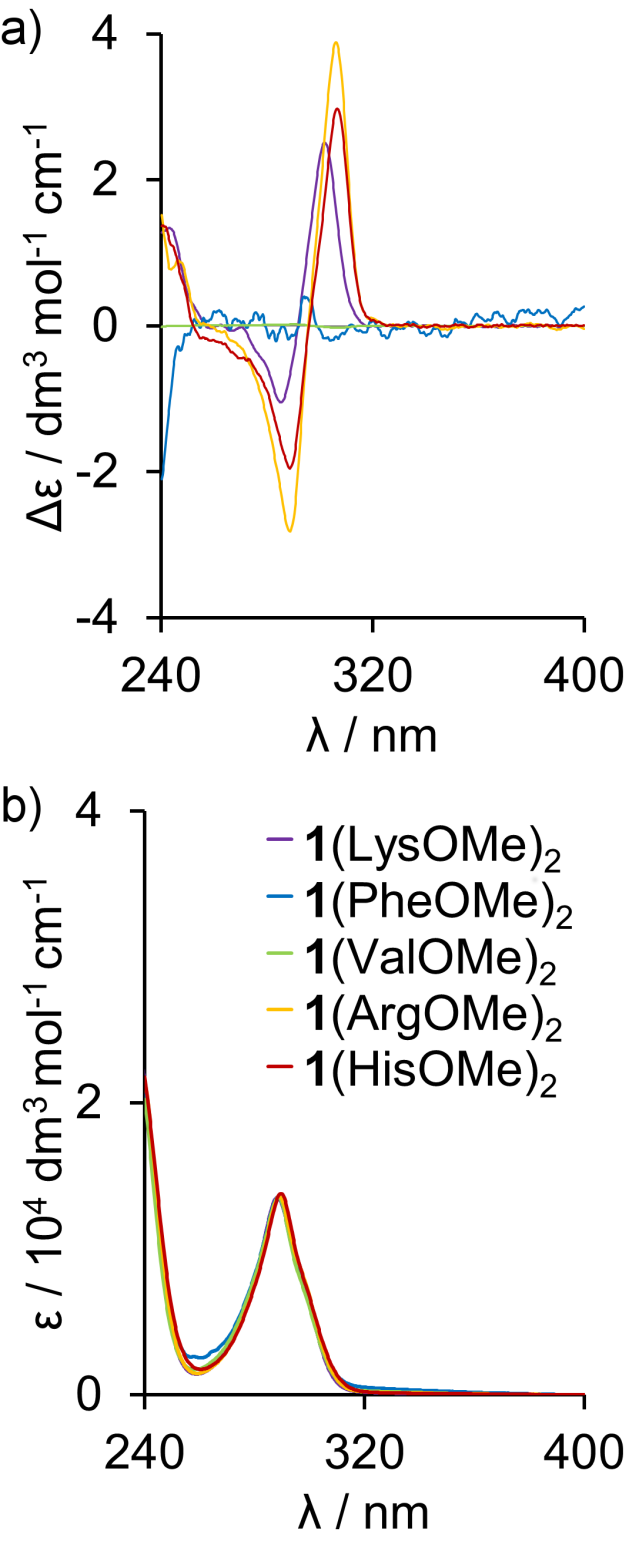
CD (a) and UV (b) spectra of complexes [**1**(LysOMe)_2_], [**1**(PheOMe)_2_], [**1**(ValOMe)_2_], [**1**(ArgOMe)_2_], and [**1**(HisOMe)_2_].

The direct evidence that the complexes are tight ion-pairs with an ordered structure comes from the diastereotopic splitting of the C*H*_2_SO_3_^−^ groups of **1** (insets in Figure 2a–g). Such splitting can only be present when chirality of the guest is transferred into the host and the effect is not averaged by dynamics and suggest formation of a well-defined structure. Additional proof of chirality transfer came from analyzing the ^1^H NMR spectra of **1** and LysOMe possessing different chirality (ʟ-, ᴅ/ʟ-, mixture of ʟ- and ᴅ/ʟ-, ᴅ-). No diastereotopic splitting was observed for [**1**(ᴅ/ʟ-LysOMe)_2_]_MeOH_ and only minor splitting was noticed for [**1**(ʟ-, ᴅ/ʟ-LysOMe)_2_]_MeOH_ (Figure S29, Supporting Information File 1). CD spectroscopy was further used to analyze complexation processes. For complexes **1**(LysOMe)_2_, **1**(ArgOMe)_2_, and **1**(HisOMe)_2_ the CD effects are observed for bands at 300 nm – in the region where chiral components are silent, whereas achiral RSAs have absorption bands (Figure 3a and Figure S65, Supporting Information File 1). These results indicate that chirality of guests is transferred to the skeleton of **1**, that, as a result, exhibits CD bands. Control CD spectra of [**1**(PheOMe)_2_]_MeOH_ and [**1**(ValOMe)_2_]_MeOH_ show no CD effects in this region (Figure 3a). These control experiments confirm that the chirality transfer does not come from nonspecific electrostatic interactions, but from the formation of conformationally well-defined complexes because specific interactions are needed for chirality transfer (Figures S30–S33, Supporting Information File 1).

It should be noted that chirality induction can be exploited to design chirality sensing ensembles [17]. This strategy was successfully implemented by the Nau group for sensing of chiral aromatic compounds. Ternary complexes between cucurbit[8]uril, dicationic dyes, and chiral aromatic analytes afford strongly induced circular dichroism (ICD) signals in the near-UV and visible regions [18]. *Endo*-functionalized molecular tubes, first reported by the Glass group [19], were used by the Jiang group for molecular recognition and chiroptical sensing of epoxides in water. The absolute configurations and ee values were simultaneously determined by circular dichroism spectroscopy [20]. Recently, dyn[4]arene, first described by the Leclaire and co-authors [21], was demonstrated to bind various lysine derivatives which leads to distinct ICD outputs in buffered aqueous solution [22]. Even a larger macrocyclic host, like hexameric resorcin[4]arene capsule, shows chirality induction from a chiral tertiary amine guest in deuterated chloroform [23].

**Figure 4:**
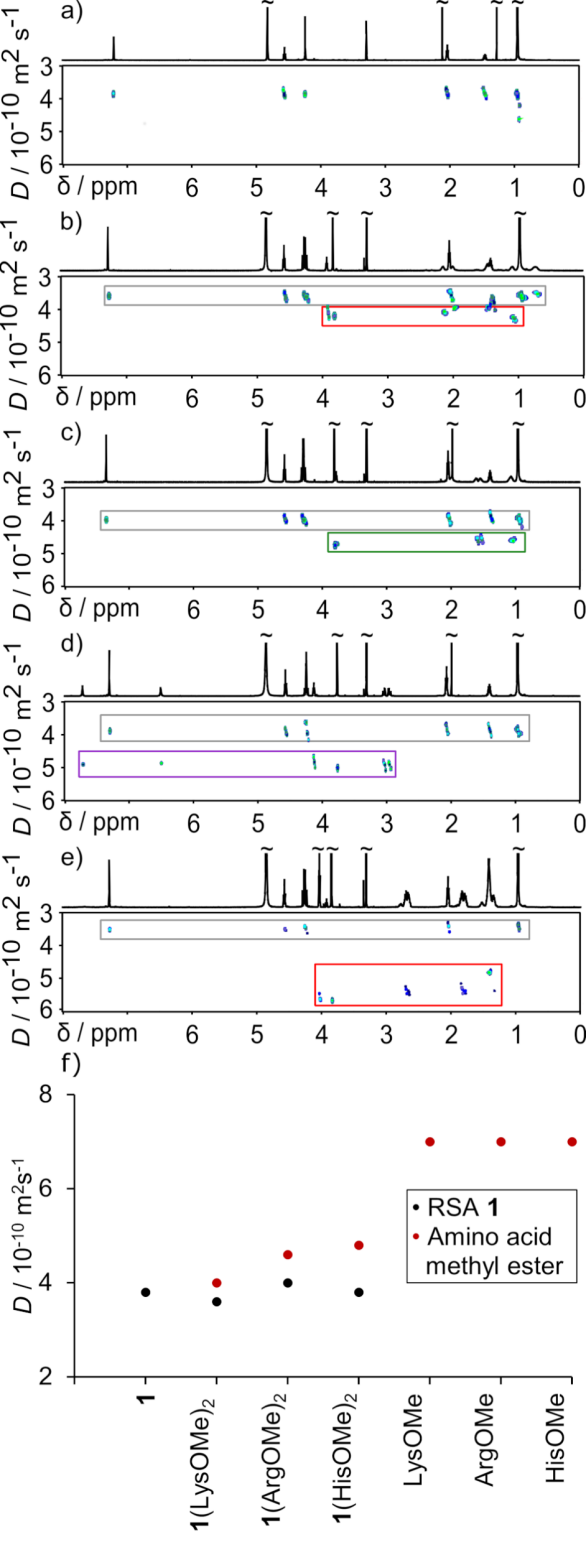
DOSY spectra of **1** (a), [**1**(LysOMe)_2_] (b), [**1**(ArgOMe)_2_] (c), [**1**(HisOMe)_2_] (d) and [LysOMe + **1**(LysOMe)_2_]_MeOH_ (e). Cumulative plot of diffusion coefficients (f) (methanol-*d*_4_, 298 K, 600 MHz).

Interestingly, there is a difference in the “degree” of guest-induced diastereomeric splitting of the host’s signal in the order **1**(LysOMe)_2_ > **1**(HisOMe)_2_ >> **1**(ArgOMe)_2_ that may come from the differences in stability of the complexes. This qualitative observation is supported by DOSY measurements (Figure 4) [24].

DOSY measurements indicate that for [**1**(LysOMe)_2_]_MeOH_ the two components of the complex exhibit noticeably different diffusion coefficients (*D*) with a lower value for **1** (3.6 × 10^−10^ m^2^ s^−1^, methanol-*d*_4_, 298 K) and slightly higher value for LysOMe (4.0 × 10^−10^ m^2^ s^−1^, methanol-*d*_4_, 298 K). This suggests that under such conditions [**1**(LysOMe)_2_]_MeOH_ partially dissociates and the LysOMe component exhibits a diffusion coefficient that has a dynamically averaged value between complex **1**(LysOMe)_2_ and free LysOMe. This interpretation is supported by an experiment with an excess of LysOMe (Figure 4e). In [LysOMe + **1**(LysOMe)_2_]_MeOH_ mixture the diffusion coefficient for the LysOMe component exhibits a considerably higher value than in [**1**(LysOMe)_2_]_MeOH_ that corresponds to a higher molar fraction of free LysOMe. On the other hand, component **1** in the [LysOMe + **1**(LysOMe)_2_] mixture has a slightly lower diffusion coefficient than in [**1**(LysOMe)_2_] which reflects a higher molar fraction of complex **1**(LysOMe)_2_. These results show that DOSY experiments are highly informative and reflect the thermodynamic stability of complexes. They also suggest that this technique may be suitable for estimation of the order of thermodynamic stability of the complexes using only a single-spectrum measurement. Assuming that the size of amino acid derivatives is roughly similar and provided that the spectra are recorded at similar concentrations under similar conditions, the diffusion coefficient reflects the molar fraction of complexed species. For the currently studied complexes the order of stability is **1**(LysOMe)_2_ > **1**(HisOMe)_2_ >> **1**(ArgOMe)_2_ (Figure 4f).

**Figure 5:**
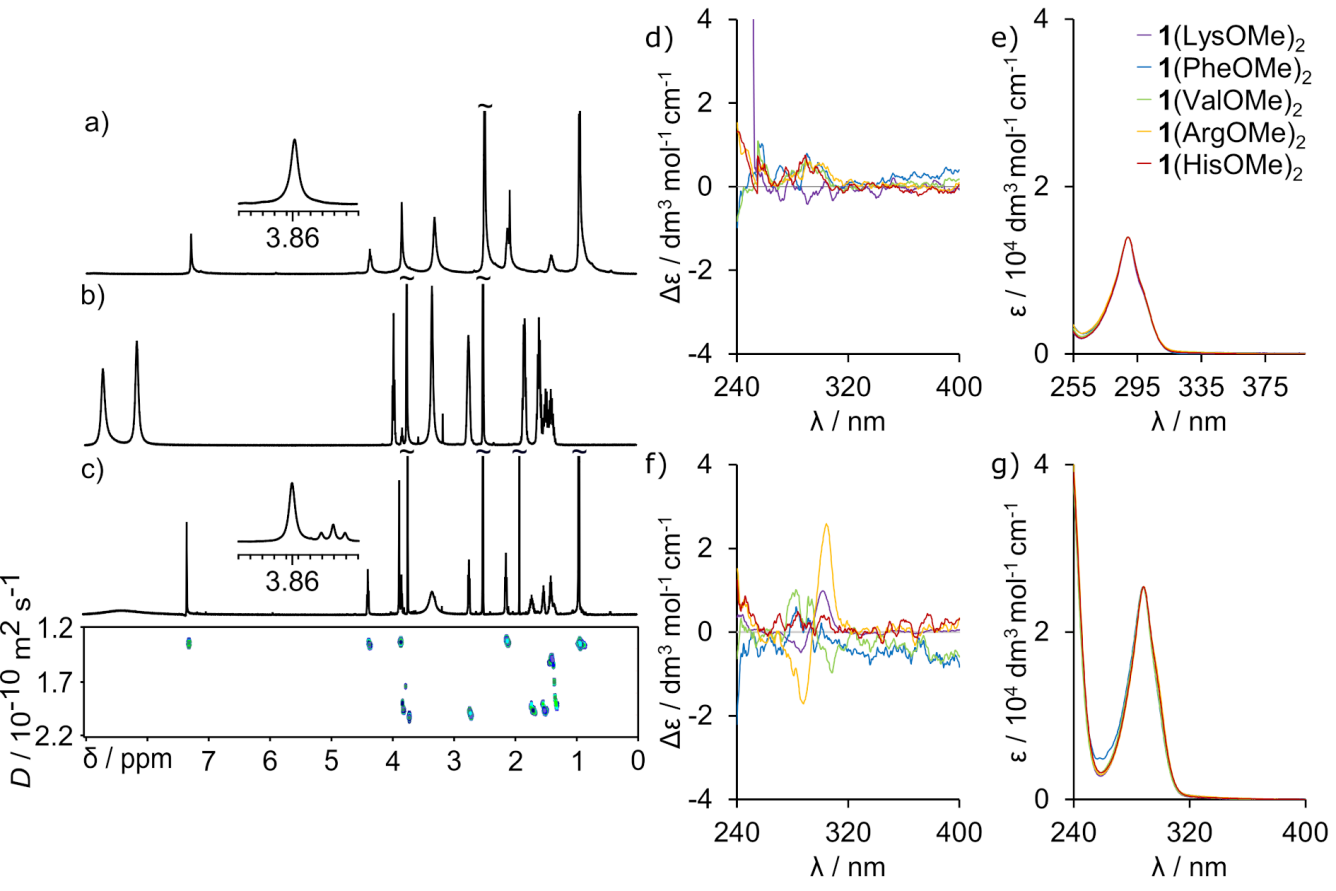
^1^H NMR spectra of **1** (a), LysOMe (b), ^1^H NMR and DOSY spectra of [**1**(LysOMe)_2_] (insets show the shape of signals f, DMSO-*d*_6_, at 298 K, 600 MHz) (c). CD (d) and UV (e) spectra in DMSO; CD (f) and UV (g) spectra in MeOH/H_2_O (1:1) mixture.^.^

A quite nonintuitive conclusion comes from a comparison of complexes in methanol and DMSO. One would expect that due to the dual hydrogen bond donor/acceptor character of methanol this solvent should be more destructive to ion-pair complexes based on charged hydrogen bonds than DMSO that can only serve as a hydrogen bond acceptor. However, we have found that complexes **1**(LysOMe)_2_, **1**(ArgOMe)_2_ and **1**(HisOMe)_2_ dissolved in DMSO-*d*_6_ disintegrate completely – there are no complexation induced shifts, the DOSY spectra show independent diffusion coefficients of the two components and CD spectra show no effects (Figure 5a–f). In order to evaluate if complex disintegration is mostly affected by the dielectric constant or by proton/acceptor affinity of the solvent we have analyzed the complexes in MeOH/H_2_O (1:1) mixture that has a similar dielectric constant to DMSO (ε(DMSO) = 46.7, ε(MeOH/H_2_O 1:1) ≈ 52). CD spectra of [**1**(LysOMe)_2_], [**1**(ArgOMe)_2_] and [**1**(HisOMe)_2_] were recorded in MeOH/H_2_O (1:1) mixture at concentrations analogous to the ones used in the experiments in MeOH and in DMSO. The intensities of the CD effects are pronouncedly lower in MeOH/H_2_O than in MeOH, but they remain detectable for [**1**(LysOMe)_2_] and [**1**(ArgOMe)_2_] (Figure 5f). This is in contrast to results in DMSO and indicate that DMSO is more disintegrative for noncovalent complexes based on electrostatic interactions, than the MeOH/H_2_O (1:1) mixture, which has a similar dielectric constant. We find this result quite nonintuitive and worth attention in the context of formation of supramolecular complexes in polar environment, for which DMSO is most often a first-choice solvent.

### Interactions of RSA **1** with chiral cavitands **2**

**Figure 6:**
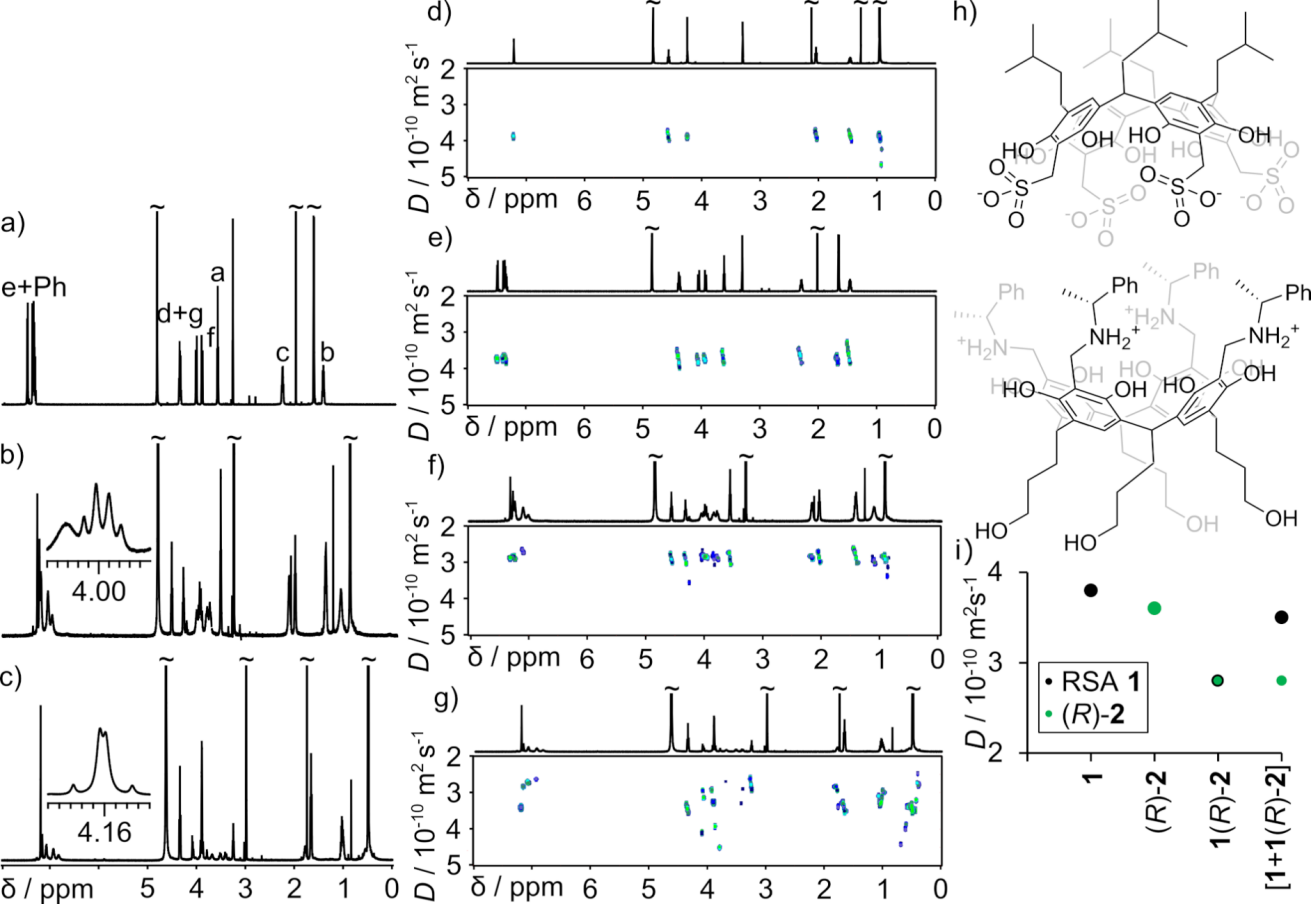
^1^H NMR spectra of (*R*)-**2** (a); [**1**((*R*)-**2**] (b); [**1** + **1**((*R*)-**2**] (c) (insets show the shape of signals f, methanol-*d*_4_, 298 K, 600 MHz); DOSY spectra of **1** (d); (*R*)-**2** (e); [**1**((*R*)-**2**)] (f); [**1** + **1**((*R*)-**2**)] (g). Proposed capsular structure of **1**((*R*)-**2**) (h); Cumulative plot of diffusion coefficients (i) (methanol-*d*_4_, 298 K, 600 MHz).

Having confirmed that RSA **1** forms well-defined complexes with chiral bidentate amines in polar solvents we next aimed at exploration of the possibilities of the formation of complexes with chiral tetradentate amines, here cavitands (*R*)-**2** and (*S*)-**2** (Figure 6). We expected that the complexes will be more stable (both thermodynamically and kinetically) due to the chelate effect and will have a capsular shape retained in polar environment. Upon mixing of water solution of **1** and (*R*)-**2** and (*S*)-**2** the complexes **1**((*R*)-**2**) and **1**((*S*)-**2**) precipitate. The complexes are soluble in MeOH, DMSO but not in water or in MeOH/H_2_O (1:1) mixture. The diffusion coefficient for [**1**((*R*)-**2**)] in methanol-*d*_4_ at 298 K is 2.8 × 10^−10^ m^2^ s^−1^ corresponding to a hydrodynamic radius of 14.3 Å (Figure 6d). The theoretically predicted radius for a capsular complex is 12.8 Å, that is in a reasonable agreement with experimental data. For comparison, the diffusion coefficient for (*R*)-**2** under the same conditions is 3.6 × 10^−10^ m^2^ s^−1^ (Figure 6e) that corresponds to a hydrodynamic radius of 11 Å. Similar to interactions of RSA **1** with bidentate amines (amino acid methyl esters), interactions with tetradenate amine (*R*)-**2** lead to the diastereotopic splitting of the signals of the methylene bridges (C*H*_2_SO_3_^−^, f) and their substantial upfield shift (Δδ 0.25 ppm, Figure 6c). The addition of an excess of **1** to a solution of **1**(*R*)-**2** in MeOH changes the ^1^H NMR and DOSY spectra (Figure 6f,g). The signals of methylene bridges (C*H*_2_SO_3_^−^, f) shift back towards the position typical for free **1** and their diastereotopic splitting is reduced (although it is not diminished completely). The DOSY spectrum shows two diffusion coefficients (Figure 6g). The first value (2.8 × 10^−10^ m^2^ s^−1^) observed for signals of the amine part (*R*)-**2** is the same in [**1** + **1**(*R*)-**2**]_MeOH_ as in [**1**(*R*)-**2**]_MeOH_ and corresponds to complex **1**(*R*)-**2**. This value indicates that in both samples (*R*)-**2** is fully complexed. The second diffusion coefficient is observed for signals of **1**, being in excess in the mixture and its value corresponds to a dynamically averaged value between **1**(*R*)-**2** and free **1**. Taken together, these results indicate that in MeOH complexation between **1** and (*R*)-**2** is effective and under the current conditions it is quantitative. The kinetics of complex formation is fast on the NMR timescale. The complexes exhibit a high degree of ordering that is manifested by chirality transfer observed by diastereotopic splitting (Figure 6c, inset). In this case CD spectra are not diagnostic (CD bands are observed already for chiral cavitands and they do not change considerably upon complexation) (Figure S63, Supporting Information File 1).

**Figure 7:**
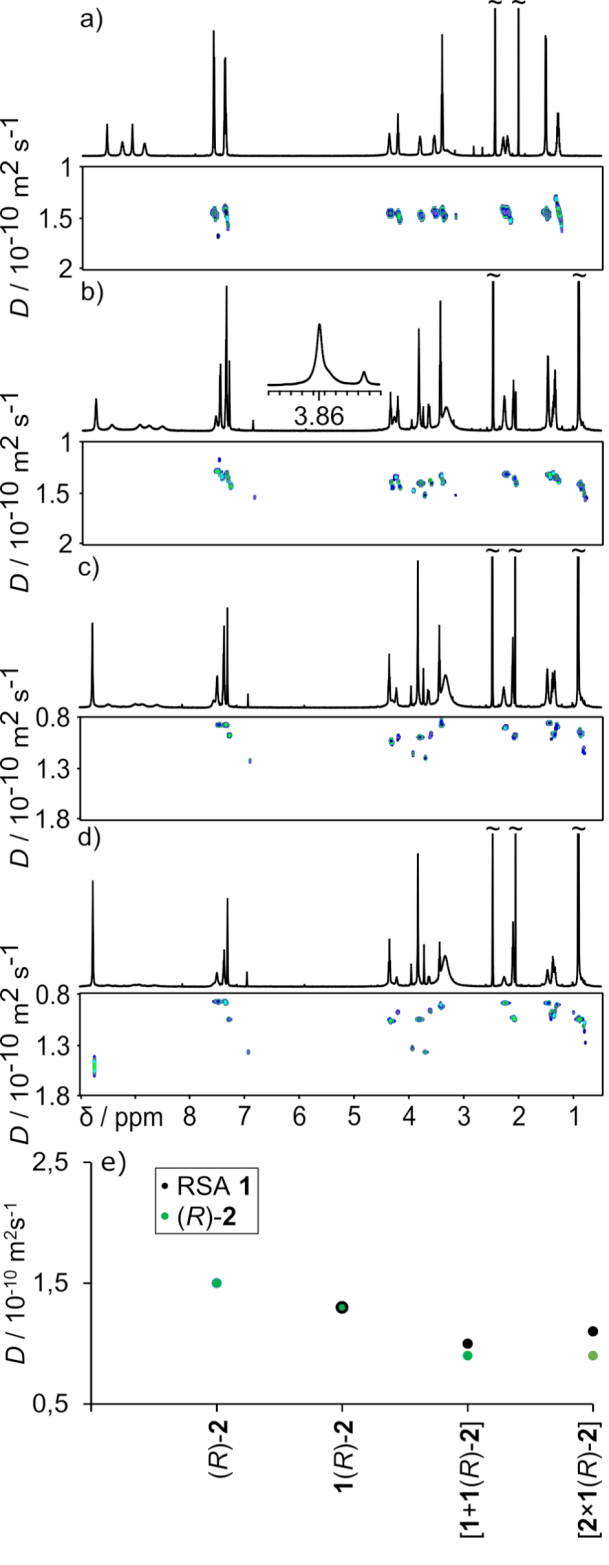
^1^H NMR and DOSY spectra of (*R*)-**2** (a); [**1**(*R*)-**2**] (b) (inset show the shape signals f, DMSO-*d*_6_, 298K, 600 MHz); [**1** + **1**(*R*)-**2**] (c); [2 × **1** + **1**(*R*)-**2**] (d); Cumulative plot of diffusion coefficients (e) (DMSO-*d*_6_, 298 K, 600 MHz).

A different behavior was observed for **1**(*R*)-**2** dissolved in DMSO. The ^1^H NMR spectrum of [**1**(*R*)-**2**)]_DMSO_ shows no changes in chemical shifts as compared with spectra of the components **1** and (*R*)-**2** (Figure 7). There are also no signs of chirality transfer (no splitting of signals of the methylene f proton in RSA part (Figure 7b, inset)), nor changes in CD bands (Figure S64, Supporting Information File 1). Considering solely 1D NMR spectra one may conclude that the complex is completely disintegrated in DMSO. However, DOSY measurements indicate that both components have the same diffusion coefficient, and this value is lower than the diffusion coefficient for free (*R*)-**2**, that may indicate that the capsular complex is actually formed. The addition of an extra amount of **1** (>2 equiv) to [**1**(*R*)-**2**] in DMSO-*d*_6_ leads to changes in DOSY – both components exhibit lower diffusion coefficients (Figure 7c). The direction of the changes is qualitatively different from the changes observed in analogous experiments in MeOH and apparently counterintuitive. However, these changes can be rationalized, and, in fact, they reveal important information about the thermodynamic and kinetics of the complexation, that cannot be obtained based on standard ^1^H NMR titration (because there are no complexation-induced shifts). We rationalize these results assuming that in [**1**(*R*)-**2**]_DMSO_ solution the complex partially disintegrates and the observed *D* is a result of a dynamic exchange between the complex **1**(*R*)-**2** with free components **1** and (*R*)-**2**. The addition of an extra portion of **1** to **1**(*R*)-**2** shifts the chemical equilibrium towards complex formation. Thus, in the mixture [**1** + **1**(*R*)-**2**]_DMSO_ the component (*R*)-**2** is fully consumed to form complex **1**(*R*)-**2** (Figure 7c). Therefore, the diffusion coefficient for (*R*)-**2** is lower in the mixture [**1** + **1**(*R*)-**2**]_DMSO_ than in [**1**(*R*)-**2**)]_DMSO_ and its value corresponds to an actual value for complex **1**(*R*)-**2**. Component **1** in mixture [**1** + **1**(*R*)-**2**)]_DMSO_ is present as complex **1**(*R*)-**2** in equilibrium with free **1**. Because the exchange is fast and because the equilibrium is shifted towards the complex, component **1** exhibits an averaged value between complex **1**(*R*)-**2** and free **1** that, in total, is higher than the value it exhibits at lower concentration. This explanation was supported by a subsequent addition of the second portion of **1** to form a mixture [2 × **1** + **1**(*R*)-**2**)]_DMSO_ that results in lowering of the diffusion coefficient for component **1** but the value for component the (*R*)-**2** remains invariant (Figure 7d). We would like to stress that this information was not accessible by standard methods based on NMR or UV/CD titrations, because complexation induces virtually no complexation-induced shifts nor UV/CD changes.

## Conclusion

We have shown that resorcin[4]arene sulfonic acid (RSA) **1** is able to strongly interact with basic amino acid derivatives (LysOMe, ArgOMe, HisOMe) forming 1:2 complexes in methanol and methanol/water mixture but not in DMSO. We have also shown that chirality of guests is reflected at the hosts’ structures by diastereomeric splitting of methylene protons (C*H*_2_SO_3_^−^, f) and by induction of considerable CD effects. These results demonstrate that CD spectroscopy can be a very sensitive technique for the detection of noncovalent interactions involving chiral molecules, even if only one component of the interaction pair is chiral. Considering strong interactions with amino acids we think that RSAs, in analogy to CSAs, can also act as modulators of protein crystallizations, especially for those proteins that have surface exposed basic amino acids [25].

The 1:1 complexes of RSA **1** with tetradentate aminocavitand (*R* or *S*)-**2** are also effectively formed in polar solvents. The complexes have capsular shape that is retained in methanol as manifested by DOSY coefficients and chirality transfer was observed in the form of diastereotopic proton splitting. The exchange between complexed and free species is fast on the NMR timescale, indicating that even for tetradentate ligands, for which a high degree of cooperativity is expected, the complexes are highly dynamic in polar medium.

Interestingly we have found that for complexes based predominately on electrostatic interactions DMSO is more disintegrative than methanol. This can be partially attributed to higher dielectric constants. However, other intrinsic solvent properties may also play a role, because the results of the analysis of complexes in MeOH/H_2_O (1:1) mixture, that have a similar dielectric constant to DMSO, reveal that in DMSO the complexes dissociate to a much higher extend. We find this result quite nonintuitive and worth attention in the context of formation of supramolecular complexes in polar environment, for which DMSO is most often a first-choice solvent.

## Experimental

### General information

All solvents and chemicals used, including sodium acetate buffer, were purchased from Sigma-Aldrich, TCI Europe N. V., Roth, Chem Impex Inc. and Eurisotop, were of reagent grade and were used without further purification. Unless noted otherwise, all reactions were carried out under an atmosphere of air.

^1^H and ^13^C NMR spectra were recorded at 303 K on Bruker 400 MHz and at 298 K on Varian VNMRS 600 MHz instruments with a residual solvent signal as an internal standard. All 2D NMR spectra were recorded at 298 K on a Varian 600 MHz instrument. Spectra were referenced to the residual solvent signals (acetone-*d*_6_, 2.05 ppm; dimethyl sulfoxide-*d*_6_, 2.50 ppm and [^13^C]dimethyl sulfoxide, 39.5 ppm; methanol-*d*_4_, 3.31 ppm and [^13^C]methanol, 49.0 ppm).

NMR DOSY experiments were performed on a Varian VNMRS-600 spectrometer equipped with a 5 mm PFG AutoXID (^1^H/X^15^N-^31^P) probe at 298 K. DOSY experiments were run with the ONESHOT or DPFGDSTE (with convection compensation) pulse sequences for measurements in methanol-*d*_4_ and DMSO-*d*_6_ solutions, respectively. The gradient strengths were incremented as a square dependence in the range from 6 to 55 G/cm. 64 transients were recorded for each increment with 2.5 s acquisition time and 1 s relaxation delay (overall experiment time of 60 min). The duration of magnetic field gradients (δ) was 2 ms, whereas a diffusion delay (Δ) was chosen as 80–100 ms in methanol-*d*_4_ and 220–250 ms in DMSO-*d*_6_, respectively. Other parameters include the following: a sweep width of 12 000 Hz, 32 K data points. The data were processed using Varian DOSY software [26].

ECD spectra were recorded on ECD Jasco J-715 spectropolarimeter.

**2,8,14,20–Tetraisobutylresorcin[4]arene** was obtained according to a literature procedure. Analytical data are in agreement with literature data [27]. The product was obtained as yellow powder, yield: 93% (33.12 g). ^1^H NMR (400 MHz, acetone-*d*_6_, δ) 8.45 (s, 8H), 7.55 (s, 4H), 6.25 (s, 4H), 4.47 (t, *J* = 8.0 Hz, 4H), 2.17 (bt, 8H), 1.48 (m, 4H), 0.95 (d, *J* = 6.7 Hz, 24H). Figure S1 (Supporting Information File 1).

**Tetrakis(sulfonatomethyl)resorcin[4]arene (1)** was obtained according to a literature procedure [15], as a pale yellow powder, yield: 92% (10.90 g). ^1^H NMR (600 MHz, methanol-*d*_4_, δ) 0.97 (d, *J* = 6.6 Hz, 24H), 1.47 (sep, *J* = 6.6 Hz, 4H), 2.06 (t, *J* = 7.4, 8H), 4.26 (s, 8H), 4.58 (t, *J* = 8.0 Hz, 4H), 7.22 (s, 4H). Figure S2 (Supporting Information File 1). ^13^C NMR (150 MHz, methanol-*d*_4_, δ) 151.7, 126.7, 124.4, 110.1, 44.5, 33.0, 27.0, 24.2, 23.3. Figure S4 (Supporting Information File 1).

***C*-(3-Hydroxypropyl)resorcin[4]arene** was obtained according to a literature procedure. Analytical data are in agreement with literature data [28]. The product was obtained as off-white powder, yield: 71% (11.60 g). ^1^H NMR (400 MHz, DMSO-*d*_6_, δ) 8.89 (s, 8H), 7.23 (s, 4H), 6.14 (s, 4H), 4.19 (t, *J* = 7.9 Hz, 4H), 3.41 (t, *J* = 6.7 Hz, 8H), 2.09 (dd, *J* = 7.8 Hz, 8H), 1.33 (m, 8H). Figure S5 (Supporting Information File 1).

**Tetrakis((*α*)–methylbenzylaminomethyl)resorcin[4]arenes** ((*R*)-**2** and (*S*)-**2**) were obtained according to the literature procedures [16]. To a solution of *C*-(3-hydroxypropyl)resorcin[4]arene (720 mg, 1 mmol) and excess formaldehyde (40% in water, 1 mL) in methanol (30 mL), (*R*)-(+)-1-phenylethylamine or (*S*)-(−)-1-phenylethylamine (650 μL, 5 equiv, 5 mmol) was added slowly and stirred at room temperature for 24 h. The precipitate that separated was filtered and dried under vacuum. The mixture of tetrabenzoxazines was not separated. Into a solution of the tetrabenzoxazine in isopropanol (50 mL) 3 mL of concentrated HCl (37%) and 4 mL of H_2_O was added and heated under reflux for 4 h. Isopropanol was evaporated and the crude product recrystallized from CH_3_CN/CH_3_OH (9:1, v/v) mixture. (*R*)-**2** was obtained as off-white powder, yield: 38% (525 mg), (*S*)-**2** was obtained as off-white powder, yield: 36% (502 mg). (*R*)-**2**: ^1^H NMR (600 MHz, methanol-*d*_4_, δ) 7.50–7.49 (m, 8H), 7.39–7.32 (m, 16H), 4.41–4.36 (m, 8H), 4.00 (dd, *J* = 12.9 Hz, 8H), 3.62 (t, *J* = 6.4 Hz, 8H), 2.30 (dd, *J* = 8.0 Hz, 8H), 1.66 (d, *J* = 6.9 Hz, 12H), 1.47 (m, 8H). Figure S34 (Supporting Information File 1). ^13^C NMR (150 MHz, methanol-*d*_4_, δ) 151.9, 151.5, 137.6, 130.5, 130.3, 128.8, 128.1, 128.0, 126.7, 110.5, 62.7, 60.2, 41.4, 35.7, 32.0, 31.0, 20.1. Figure S36 (Supporting Information File 1).

(*R*)-**2**: ^1^H NMR (600 MHz, DMSO-*d*_6_, δ) 9.56 (s, 4H), 9.28 (s, 4H), 9.10 (s, 4H), 8.88 (s, 4H), 7.62–7.61 (m, 12H), 7.43–7.38 (m, 12 H), 4.42 (bs, 4H), 4.27 (t, *J* = 7.8 Hz, 4H), 3.87 (bs, 4H), 3.60 (bs, 4H), 3.46 (t, *J* = 6.6 Hz, 8H), 2.37–2.25 (m, 8H), 1.58 (d, *J* = 6.6 Hz, 12H), 1.39–1.31 (m, 8H). Figure S43 (Supporting Information File 1). ^13^C NMR (150 MHz, DMSO-*d*_6_, δ) 150.3, 149.7, 137.0, 128.9, 128.8, 127.9, 126.7, 126.0, 109.7, 60.3, 58.1, 40.1, 34.1, 31.0, 28.8, 19.9. Figure S45 (Supporting Information File 1).

(*S*)-**2**: ^1^H NMR (400 MHz, methanol-*d*_4_, δ) 7.50–7.45 (m, 8H), 7.39–7.31 (m, 16H), 4.42–4.35 (m, 8H), 4.00 (dd, *J* = 12.9 Hz, 8H), 3.62 (t, *J* = 6.3 Hz, 8 H), 2.30 (dd, *J* = 7.9 Hz, 8H), 1.66 (d, *J* = 6.9 Hz, 12H), 1.47 (m, 8H). Figure S40 (Supporting Information File 1). ^13^C NMR (100 MHz, methanol-*d*_4_, δ) 151.9, 151.6, 137.6, 130.5, 130.3, 128.8, 128.1, 128.0, 126.7, 110.6, 62.7, 60.2, 41.5, 35.7, 32.0, 31.1, 20.1. Figure S42 (Supporting Information File 1).

**Complex formation:** All complexes were obtained by dissolving tetrakis(sulfonatomethyl)resorcin[4]arene **1** (11 mg, 0.01 mmol) in sodium acetate buffer (pH 7, 1 mL) and subsequent addition of amino acid methyl esters in form of hydrochloride salts (5 equiv, 0.05 mmol): H-Lys-OMe (∙2HCl), H-ᴅ-Lys-OMe (∙2HCl), H-ᴅ/ʟ-Lys-OMe (∙2HCl), H-Arg-OMe (∙2HCl), H-His-OMe (∙2HCl), H-Phe-OMe (∙HCl), H-Val-OMe (∙HCl). After a few minutes, complexes of **1** with lysine, arginine and histidine methyl esters precipitated. The obtained solids were isolated and dried in vacuum.

**1**(LysOMe)_2_, yield 71% (10 mg). ^1^H NMR (600 MHz, methanol-*d*_4_, δ) 7.28 (s, 4H), 4.58 (t, *J* = 7.9 Hz, 4H), 4.26 (dd, *J* = 14.0 Hz, 8H), 3.92 (t, *J* = 3.9 Hz, 2H), 3.83 (s, 6H), 2.15–2.13 (m, 3H), 2.04 (t, *J* = 7.3 Hz, 8H), 2.00–1.99 (m, 3H), 1.48–1.38 (m, 10H), 0.96 (d, *J* = 6.6 Hz, 28H), 0.73 (m, 6H). Figure S6 (Supporting Information File 1). ^13^C NMR (150 MHz, methanol-*d*_4_, δ) 170.8, 152.0, 151.9, 126.8, 126.8, 124.9, 109.7, 53.8, 53.7, 44.9, 39.8, 33.0, 30.2, 27.1, 26.9, 23.2, 23.2, 22.1. Figure S8 (Supporting Information File 1).

**1**(ArgOMe)_2_, yield 61% (9 mg). ^1^H NMR (600 MHz, methanol-*d*_4_, δ) 7.36 (s, 4H), 4.59 (t, *J* = 8.0 Hz, 4H), 4.29 (dd, *J* = 14.0 Hz, 8H), 3.81 (s, 7H), 3.78 (t, *J* = 6.7 Hz, 2H), 2.04 (t, *J* = 7.4 Hz, 8H), 1.98 (m, 2H), 1.61 (m, 2H), 1.54 (m, 2H), 1.39 (sep, *J* = 6.5 Hz, 4H), 1.06 (m, 5H), 0.96 (d, *J* = 6.6 Hz, 24H). Figure S12 (Supporting Information File 1). ^13^C NMR (150 MHz, methanol-*d*_4_, δ) 170.3, 156.3, 150.7, 150.8, 125.2, 123.7, 107.6, 52.3, 52.2, 48.1, 43.8, 39.3, 31.3, 27.4, 25.7, 23.9, 21.7, 21.7. Figure S14 (Supporting Information File 1).

**1**(HisOMe)_2_, yield 70% (10 mg). ^1^H NMR (600 MHz, methanol-*d*_4_, δ) 7.72 (s, 2H), 7.31 (s, 8H), 6.51 (s, 2H), 4.57 (t, *J* = 8.0, 4H), 4.25 (m, 8H), 4.13 (t, *J* = 6.4 Hz, 2H), 3.77 (s, 7H), 3.00 (ddd, *J* = 6.47 Hz, 5H), 2.07 (t, *J* = 7.4 Hz, 8H), 1.40 (sep, *J* = 6.8 Hz, 4H), 0.96 (d, *J* = 6.6 Hz, 24H). Figure S18 (Supporting Information File 1). ^13^C NMR (150 MHz, methanol-*d*_4_, δ) 184.6, 169.5, 152.1, 135.6, 126.9, 124.3, 118.7, 110.1, 106.4, 74.8, 54.1, 53.1, 49.6, 44.4, 33.0, 27.2, 26.5, 23.2, 20.7. Figure S20 (Supporting Information File 1).

**1**(ᴅ-LysOMe)_2_, yield 71% (10 mg). ^1^H NMR (400 MHz, methanol-*d*_4_, δ) 7.28 (s, 4H), 4.58 (t, *J* = 7.9 Hz, 4H), 4.26 (dd, *J* = 13.9 Hz, 8H), 3.93 (t, *J* = 6.1 Hz, 3H), 3.83 (s, 8H), 2.26–2.22 (m, 3H), 2.13–2.10 (m, 3H), 2.04 (t, *J* = 7.3 Hz, 8H), 1.54–1.50 (m, 5H), 1.41 (m, 4H), 1.14 (m, 4H), 1.05 (m, 3H), 0.96 (d, *J* = 6.6 Hz, 24H), 0.90–0.85 (m, 6H). Figure S24 (Supporting Information File 1).

**1**(ᴅ/ʟ-LysOMe)_2_, yield 71% (10 mg). ^1^H NMR (400 MHz, methanol-*d*_4_, δ) 7.27 (s, 4H), 4.58 (t, *J* = 7.9 Hz, 4H), 4.31–4.22 (m, 8H), 3.91 (t, *J* = 6.1 Hz, 2H), 3.82 (s, 7H), 2.22–2.07 (m, 3H), 2.03 (t, *J* = 7.3 Hz, 11H), 1.56–1.36 (m, 11H), 1.12–1.01 (m, 7H), 0.96 (d, *J* = 6.6 Hz, 24H), 0.80 (m, 5H). Figure S25 (Supporting Information File 1).

**1**(ʟ-,ᴅ/ʟ-LysOMe)_2_, yield 71% (10 mg). ^1^H NMR (400 MHz, methanol-*d*_4_, δ) 7.28 (s, 4H), 4.58 (t, *J* = 7.9 Hz, 4H), 4.30–4.22 (m, 8H), 3.92 (t, *J* = 6.1 Hz, 2H), 3.83 (s, 7H), 2.19–2.13 (m, 3H), 2.04 (t, *J* = 7.3 Hz, 8H), 1.53–1.36 (m, 9H), 1.11–1.04 (m, 5H), 0.96 (d, *J* = 6.6 Hz, 26H), 0.81–0.74 (m, 4H). Figure S26 (Supporting Information File 1).

Complexes of **1** with phenylalanine and valine methyl esters were prepared by mixing tetrakis(sulfonatomethyl)resorcin[4]arene **1** with hydrochloride salts of amino acids methyl esters and subsequent lyophilization.

**1**(PheOMe)_2_, yield >99% (22 mg). ^1^H NMR (400 MHz, methanol-*d*_4_, δ) 7.34–7.21 (m, 24H), 4.58 (t, *J* = 7.9 Hz, 4H), 4.22 (m, 12H), 3.74 (s, 10H), 3.24–3.09 (m, 8H), 2.05 (t, *J* = 7.3 Hz, 8H), 1.51–1.42 (m, 4H), 0.96 (d, *J* = 6.6 Hz, 24H). Figure S27 (Supporting Information File 1).

**1**(ValOMe)_2_, yield >99% (20 mg). ^1^H NMR (400 MHz, methanol-*d*_4_, δ) 7.25 (s, 4H), 4.58 (t, *J* = 8.0 Hz, 4H), 4.25 (s, 8H), 3.82 (s, 20H), 2.18–2.10 (m, 6H), 2.06 (t, *J* = 7.3 Hz, 8H), 1.51–1.43 (m, 4H), 0.97 (d, *J* = 6.6 Hz, 24H), 0.91 (dd, *J* = 7.0 Hz, 36H). Figure S28 (Supporting Information File 1).

**Capsule formation**: Capsules formed between tetrakis(sulfonatomethyl)resorcin[4]arene **1** (11 mg, 0.01 mmol) and tetrakis((α)-methylbenzylaminomethyl)resorcin[4]arenes (*R*)-**2** or (*S*)-**2** (14 mg, 0.01 mmol) were prepared by dissolving in water (1 mL) and mixing two resorcin[4]arene derivative solutions at room temperature. Immediately after combining two dissolved cavitands capsules were formed. Obtained solids were isolated and dried in vacuum. **1** + (*R*)-**2** (yield 51%, 12 mg): ^1^H NMR (600 MHz, methanol-*d*_4_, δ) 7.30 (s, 4H), 7.25–7.22 (m, 12H), 7.08 (bs, 8H), 6.99 (bs, 4H), 4.57 (t, *J* = 7.9 Hz, 4H), 4.33 (t, *J* = 7.6 Hz, 4H), 4.06 (bs, 4H), 3.99 (dd, *J* = 13.8 Hz, 8H), 3.85 (m, 4H), 3.80 (m, 4H), 3.58 (t, *J* = 6.3 Hz, 8H), 2.20 (m, 8H), 2.07 (t, *J* = 7.2 Hz, 8H), 1.48–1.45 (m, 12H), 1.14 (bs, 12H), 0.96 (d, *J* = 6.6 Hz, 24H). Figure S49 (Supporting Information File 1). ^13^C NMR (150 MHz, methanol-*d*_4_, δ) 152.1, 1520., 151.8, 151.7, 137.5, 130.2, 130.0, 128.5, 127.0, 126.7, 126.7, 124.7, 109.9, 62.9, 59.9, 49.6, 44.5, 35.5, 33.1, 32.0, 31.5, 27.1, 24.2, 23.3. Figure S51 (Supporting Information File 1).

**1** + (*R*)-**2**: ^1^H NMR (600 MHz, DMSO-*d*_6_, δ) 9.76 (s, 6H), 9,46 (bs, 5H), 8.96 (bs, 5H), 8.78 (bs, 5H), 8.54 (bs, 5H), 7.56 (bs, 4H), 7.49–7.48 (m, 8H), 7.39–7.35 (m, 12H), 7.32 (s, 4H), 4.38 (t, *J* = 7.7 Hz, 4H), 4.31 (bs, 4H), 4.24 (t, *J* = 7.4 Hz, 4H), 3.86 (s, 8H), 3.78–3.66 (m, 8H), 3.46 (st, *J* = 6.6 Hz, 8H), 2.29 (bs, 8H), 2.13 (t, *J* = 6.9 Hz, 8H), 1.50 (d, *J* = 4.9 Hz, 12H), 1.42–1.35 (m, 12H), 0.93 (d, *J* = 6.6 Hz, 24H). Figure S55 (Supporting Information File 1). ^13^C NMR (150 MHz, DMSO-*d*_6_, δ) 150.0, 137.0, 128.8, 127.7, 125.9, 124.8, 123.1, 112.9, 109.0, 96.7, 60.4, 58.0, 48.2, 42.2, 40.1, 34.0, 31.7, 28.9, 25.8, 22.9, 19.6. Figure S57 (Supporting Information File 1).

**1** + (*S*)-**2** (yield 47%, 11 mg): ^1^H NMR (400 MHz, methanol-*d*_4_, δ) 7.30 (s, 4H), 7.25–7.22 (m, 12H), 7.08 (bs, 8H), 7.00 (bs, 4H), 4.57 (t, *J* = 7.9 Hz, 4H), 4.34 (t, *J* = 7.8 Hz, 4H), 4.06 (bs, 4H), 4.0 (dd, *J* = 14.3 Hz, 8H), 3.85–3.78 (m, 8H), 3.58 (t, *J* = 6.3 Hz, 8H), 2.20 (m, 8H), 2.07 (t, *J* = 7.2 Hz, 8H), 1.48–1.45 (m, 12H), 1.14 (bs, 12H), 0.96 (d, *J* = 6.6 Hz, 24H). Figure S61 (Supporting Information File 1).

## Supporting Information

File 1 Copies of NMR, UV, and CD spectra.
