# Supplementary material for: Complexation of chiral amines by resorcin[4]arene sulfonic acids in polar media – circular dichroism and diffusion studies of chirality transfer and solvent dependence
Source: Beilstein J Org Chem. 2019 Aug 12;15:1913–24. doi: 10.3762/bjoc.15.187 (PMC6720235; doi:10.3762/bjoc.15.187)
Supplement: File 1 — Copies of NMR, UV, and CD spectra. [file Beilstein_J_Org_Chem-15-1913-s001.pdf]

BEILSTEIN JOURNAL OF ORGANIC CHEMISTRY

Supporting Information

for

Complexation of chiral amines by resorcin[4]arene sulfonic acids in polar media – circular dichroism and diffusion studies of chirality transfer and solvent dependence

Bartosz Setner and Agnieszka Szumna

Beilstein J. Org. Chem. **2019,** *15,* 1913–1924. doi:10.3762/bjoc.15.187

Copies of NMR, UV, and CD spectra

License and Terms: This is a supporting information file under the terms of the Creative Commons Attribution License (http://creativecommons.org/licenses/by/4.0). Please note that the reuse, redistribution and reproduction in particular requires that the authors and source are credited.
The license is subject to the *Beilstein Journal of Organic Chemistry* terms and conditions: (https://www.beilstein-journals.org/bjoc)

Table of content

1. NMR spectra S2

1.1. NMR spectra of cavitands S2

1.2. NMR spectra of complexes of tetrakis(sulfonatomethyl)resorcin[4]arene **1** with amino acids esters S5

1.3. NMR spectra of chiral cavitands S33

1.4. NMR spectra of capsules S41

2. UV and ECD spectra S48

3. ESIMS spectrum S50

1. NMR spectra

1.1. NMR spectra of cavitands

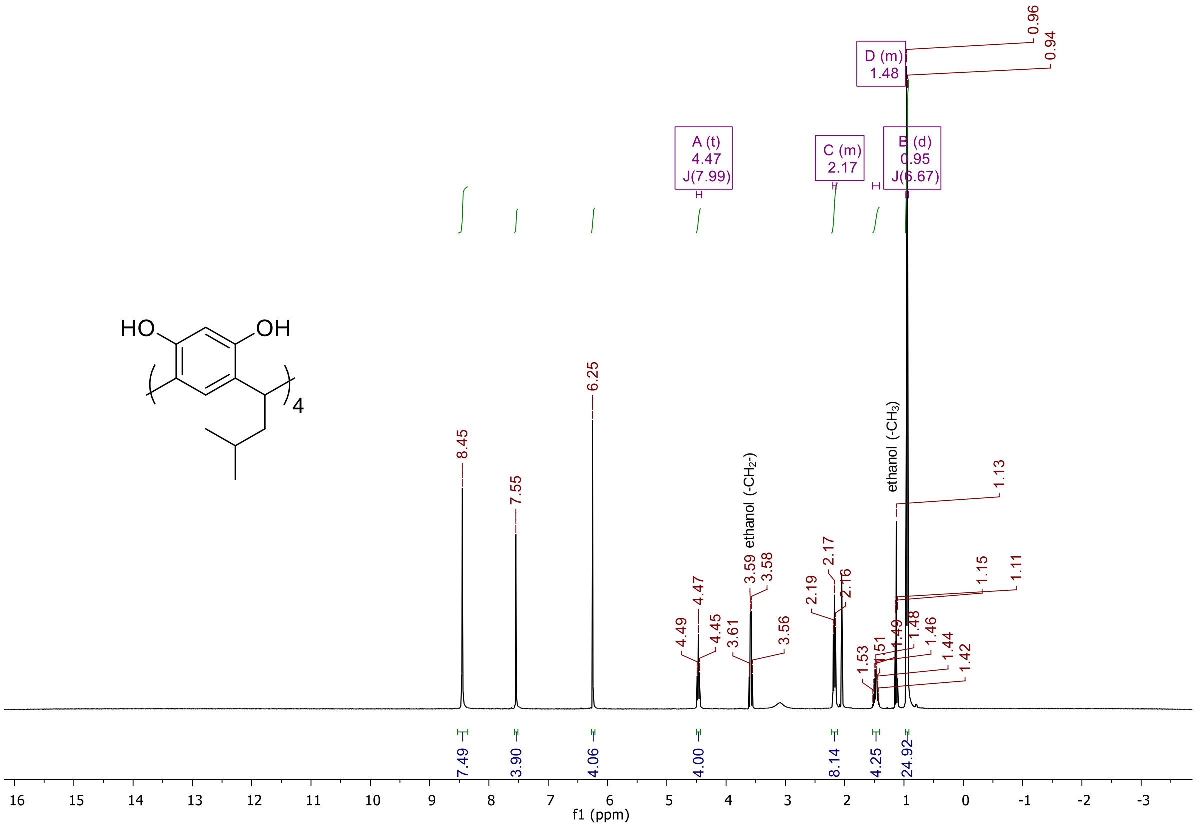

Figure S1. ^{1}H NMR (400 MHz, acetone-d_6) spectrum of 2,8,14,20-tetraisobutyl-resorcin[4]arene.

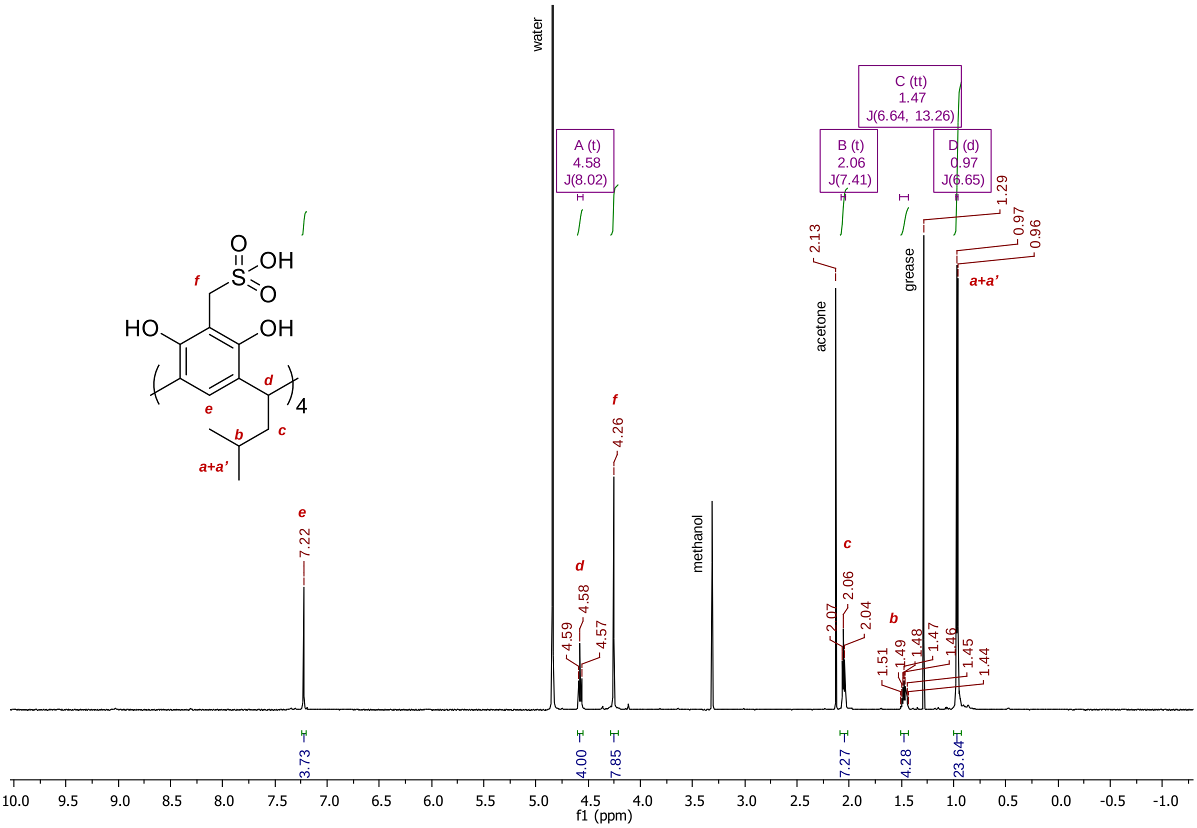

Figure S2. ^{1}H NMR (600 MHz, methanol-d_4) spectrum of **1**.

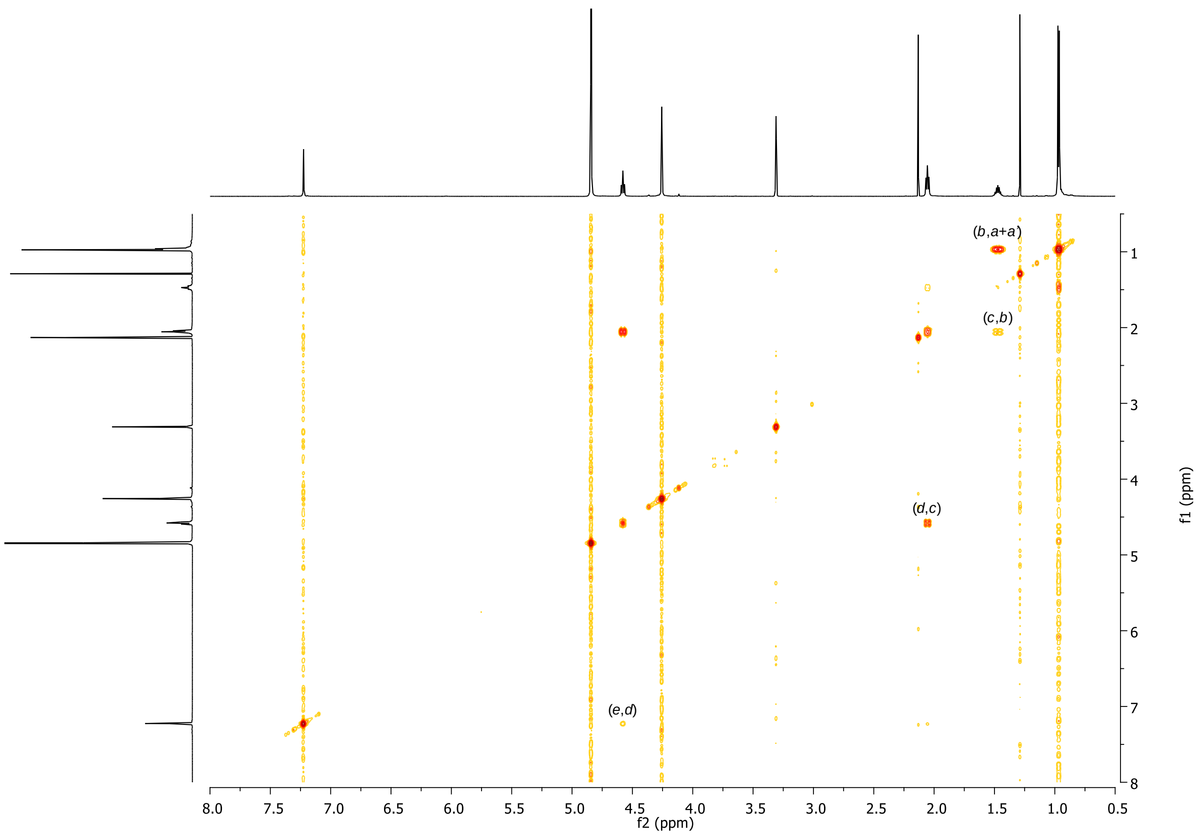

Figure S3. COSY NMR (600 MHz, methanol-d_4) spectrum of **1**.

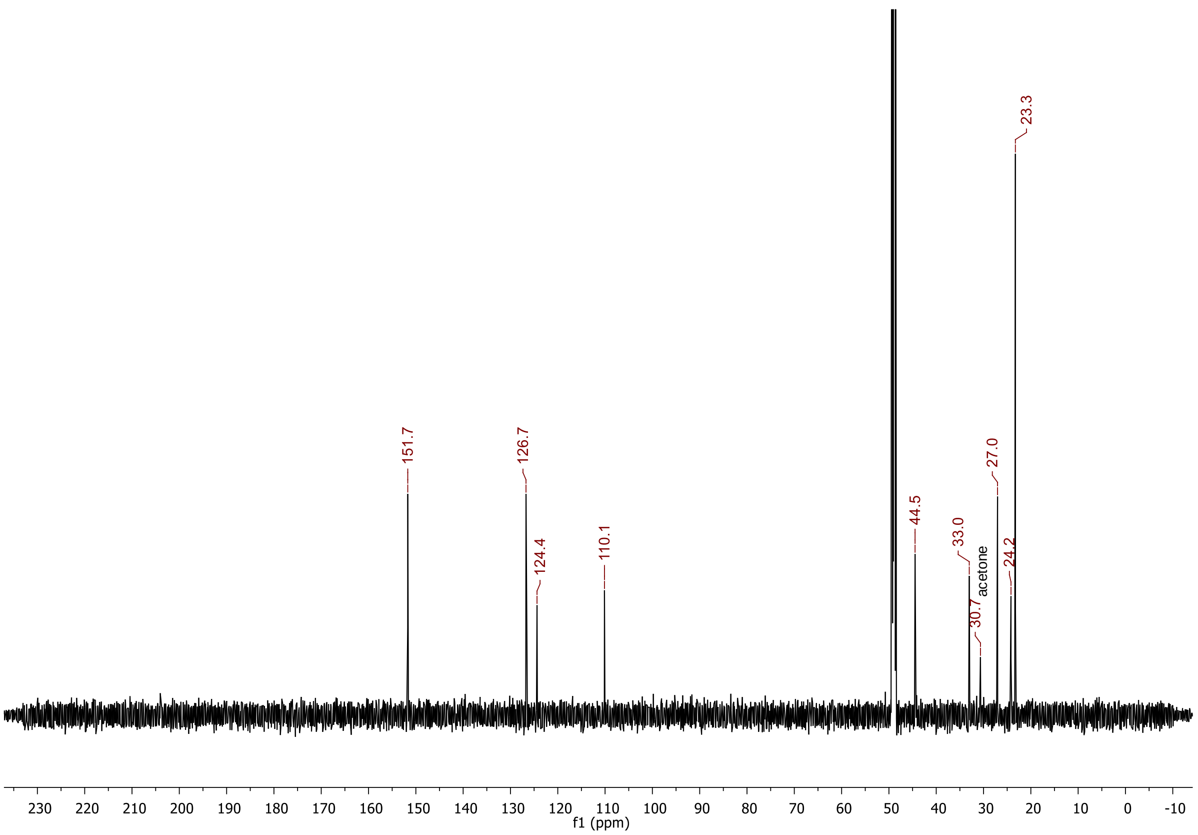

Figure S4. ^{13}C NMR (150 MHz, methanol-d_4) spectrum of **1**.

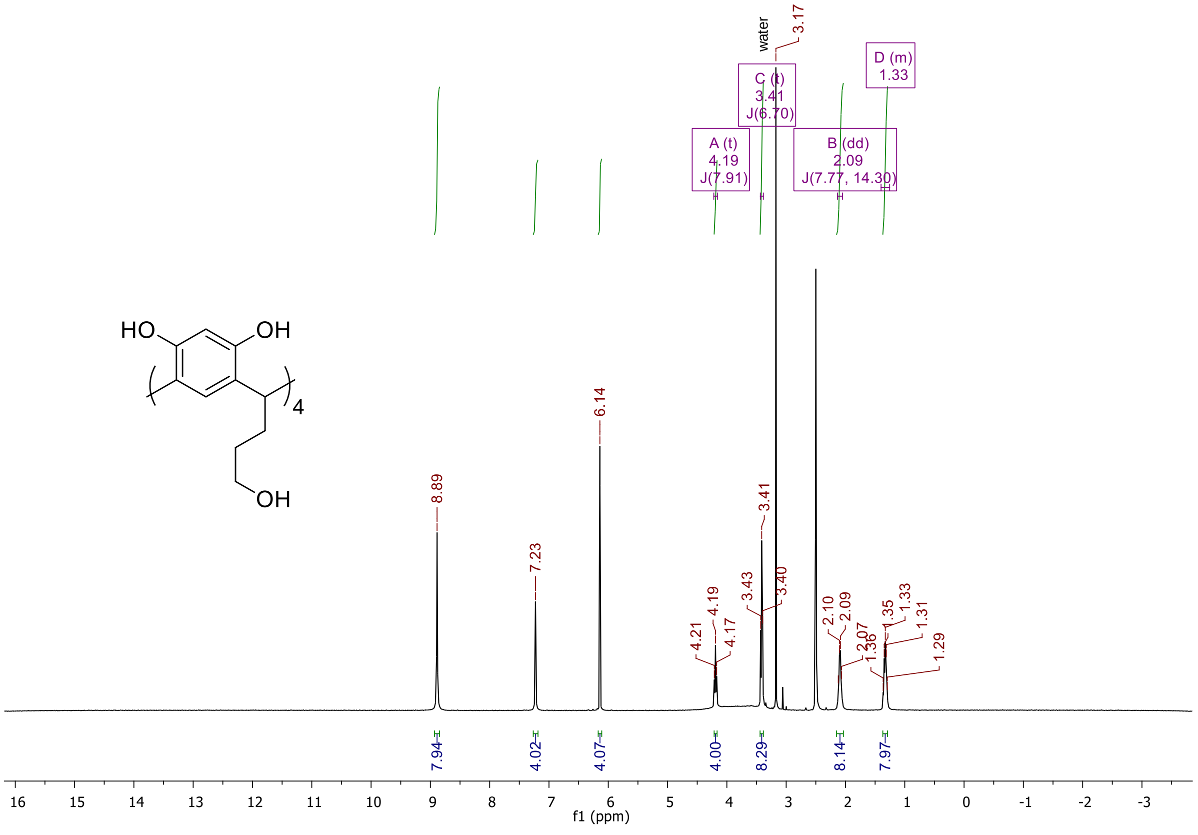

Figure S5. ^{1}H NMR (400 MHz, dimethyl sulfoxide-d_6) spectrum of C-(3-hydroxypropyl)-resorcin[4]arene.

1.2. NMR spectra of complexes of tetrakis(sulfonatomethyl)resorcin[4]arene **1** with amino acids esters

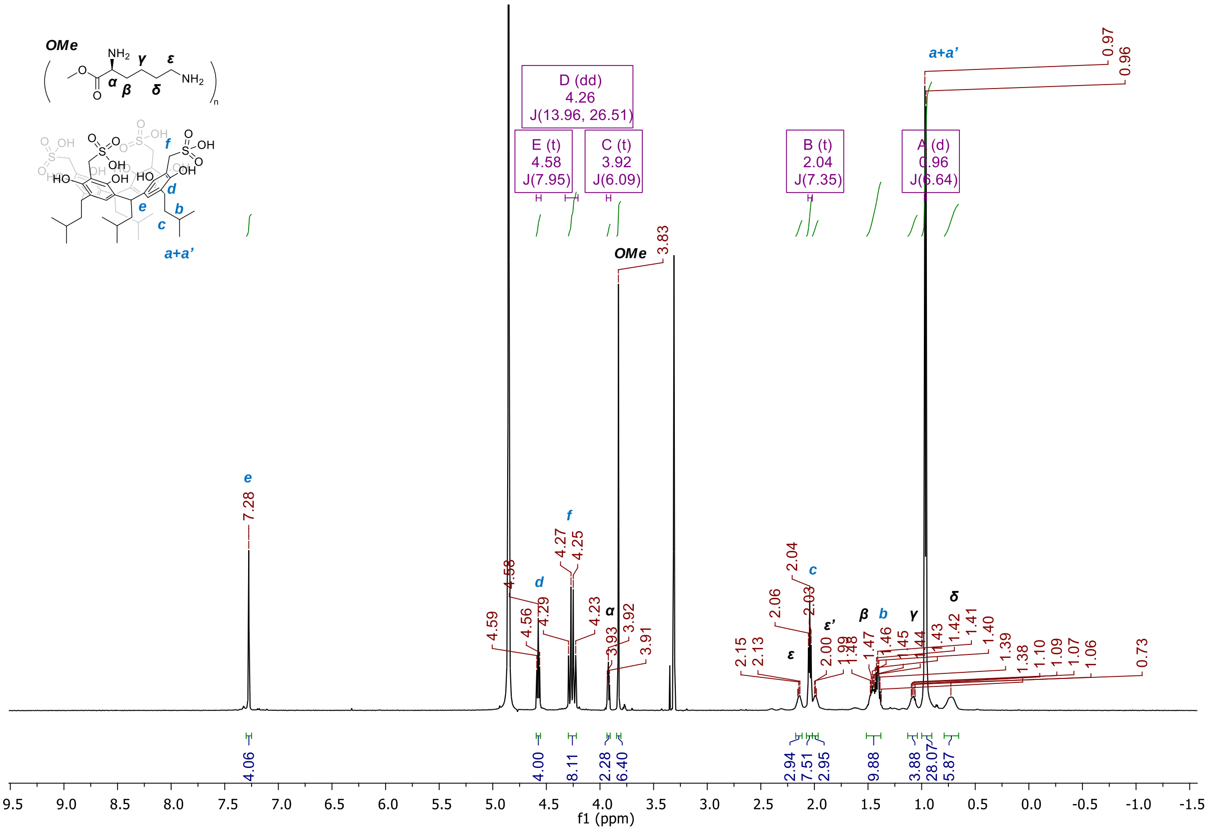

Figure S6. ^{1}H NMR (600 MHz, methanol-d_4) spectrum of complex of **1** with L-lysine methyl ester.

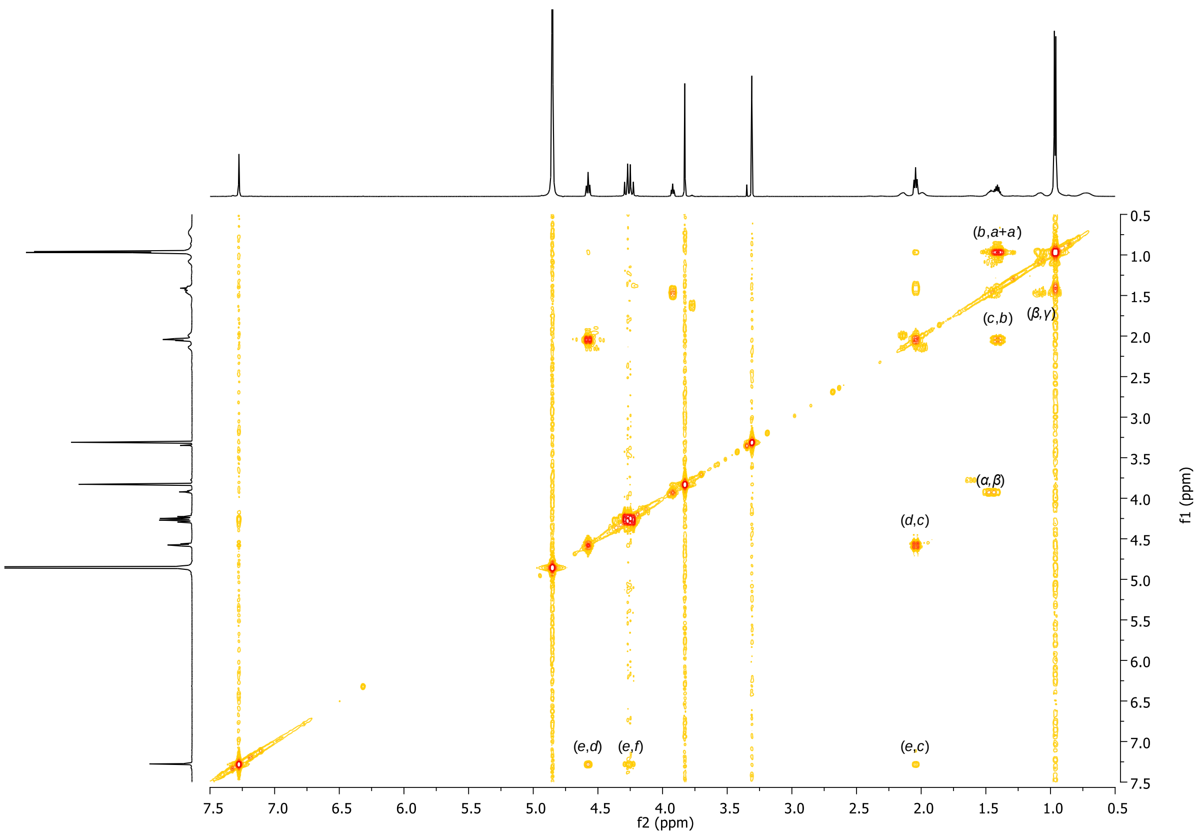

Figure S7. COSY NMR (600 MHz, methanol-d_4) spectrum of complex of **1** with L-lysine methyl ester.

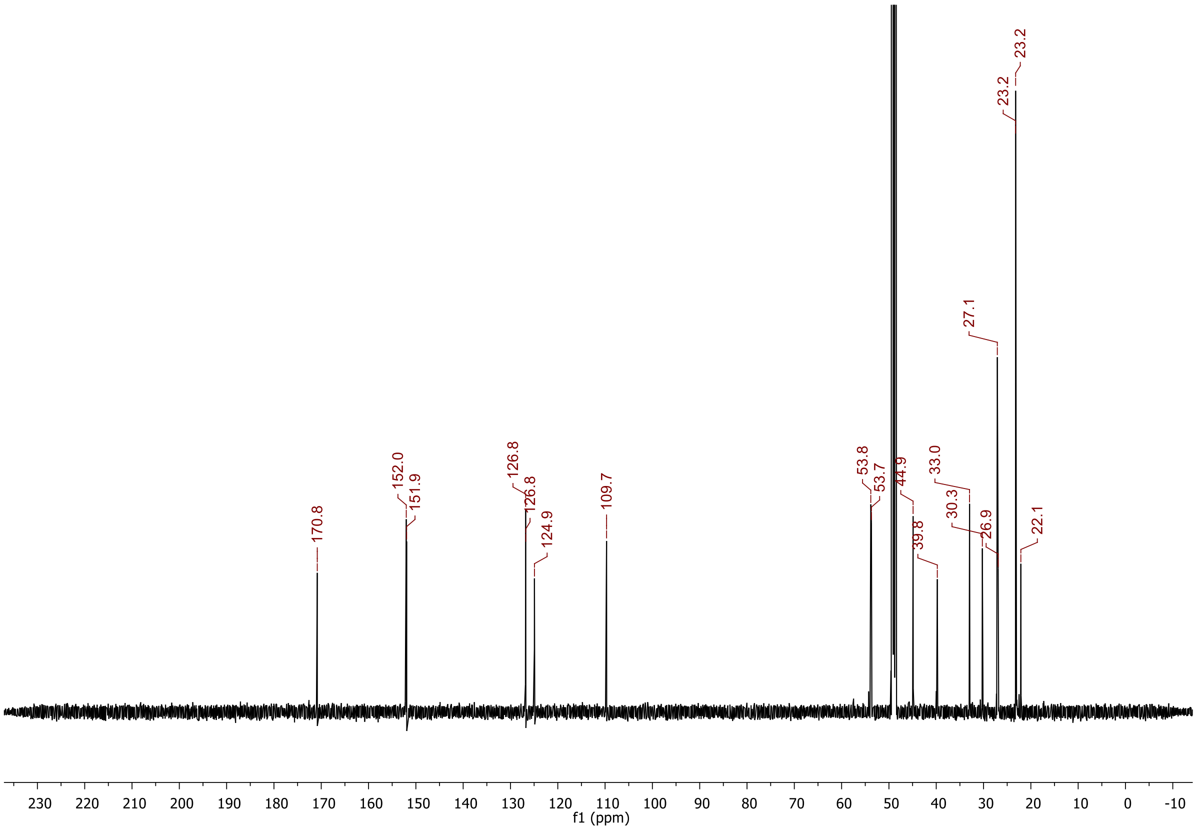

Figure S8. ^{13}C NMR (150 MHz, methanol-d_4) spectrum of complex of **1** with L-lysine methyl ester.

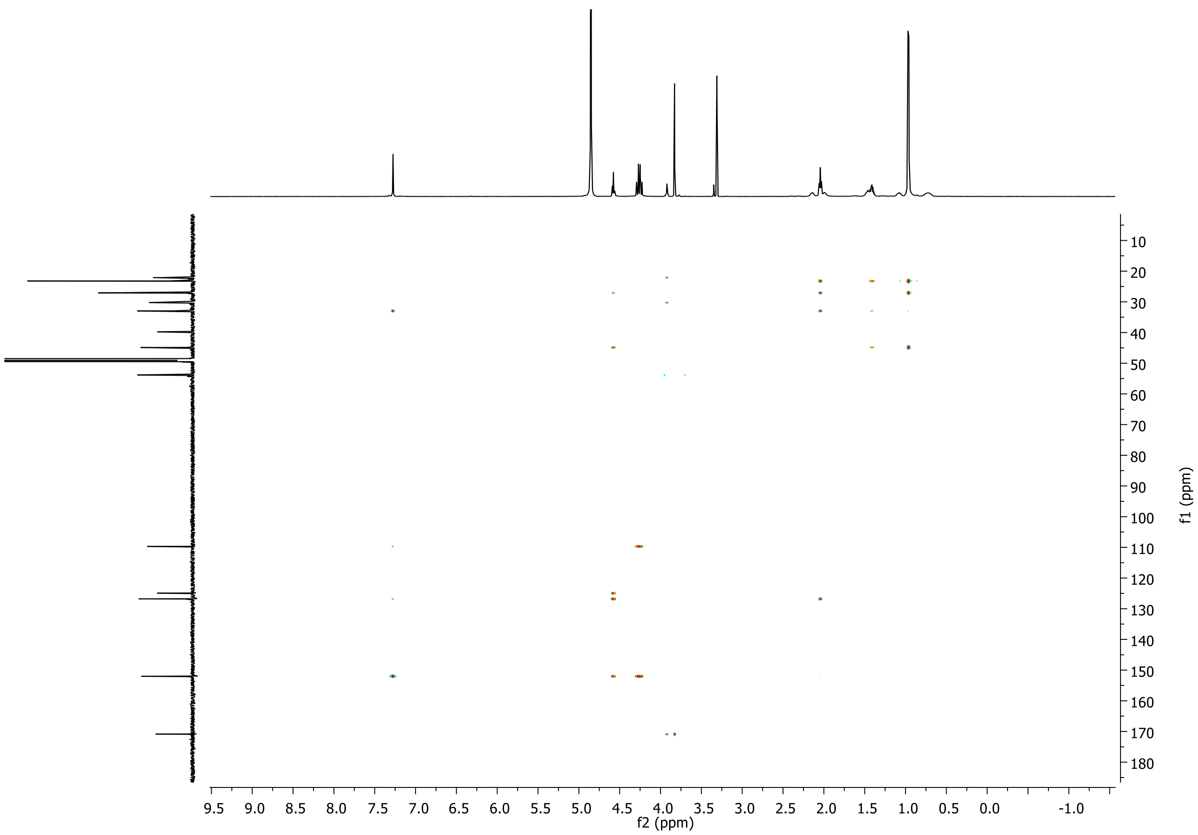

Figure S9. HMBC NMR (600 MHz, methanol-d_4) spectrum of complex of **1** with L-lysine methyl ester.

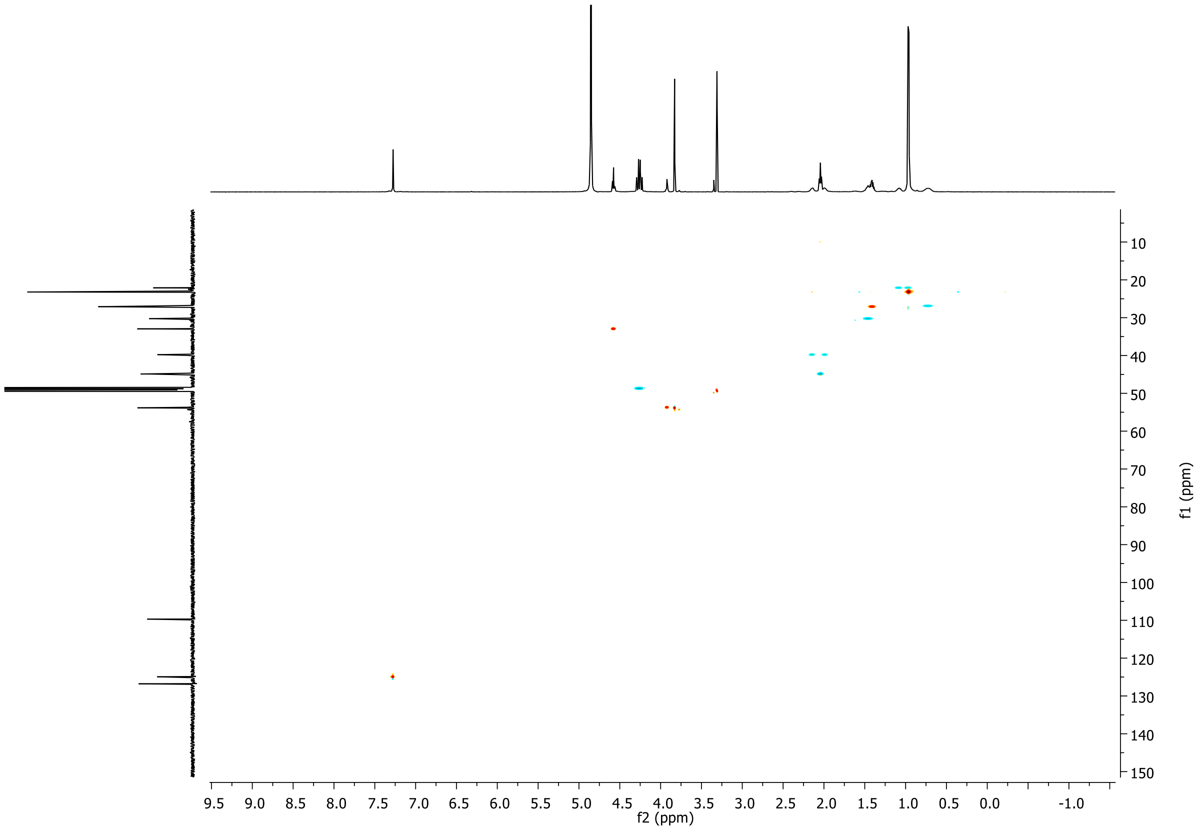

Figure S10. HSQC NMR (600 MHz, methanol-d_4) spectrum of complex of **1** with L-lysine methyl ester.

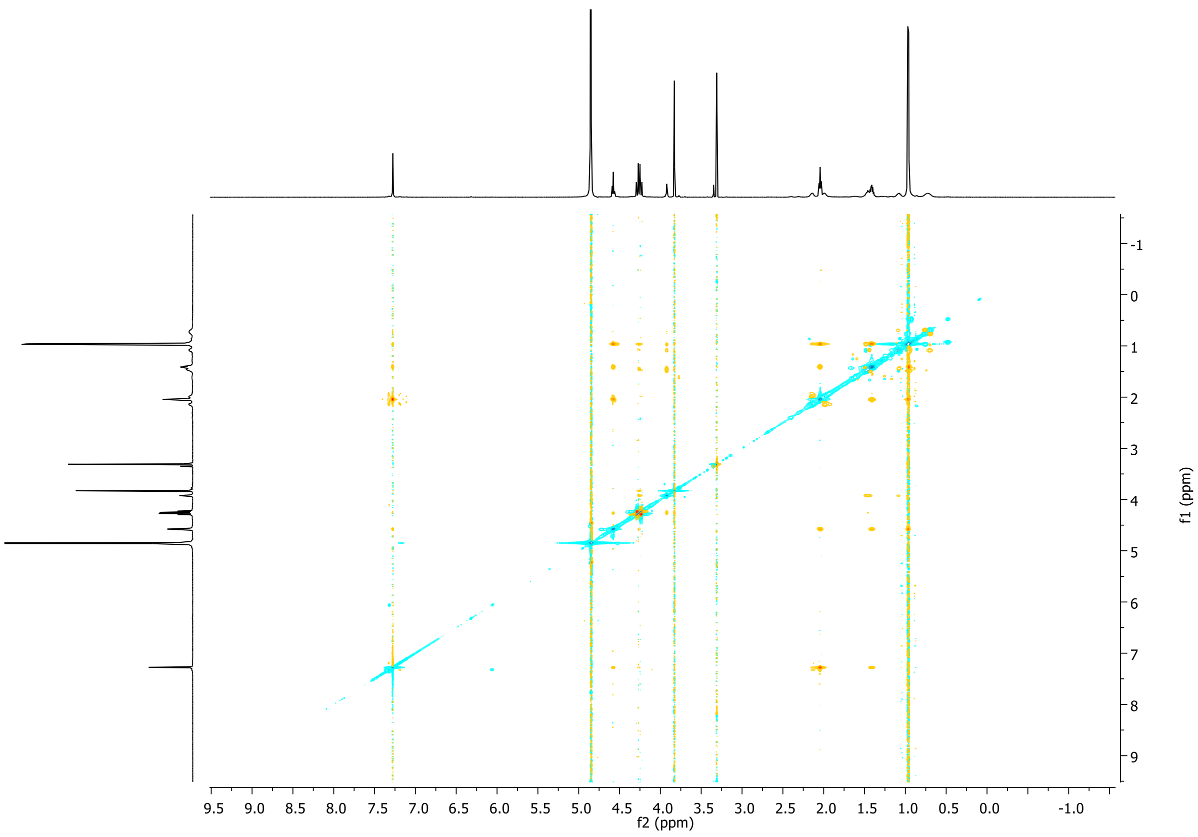

Figure S11. ROESY NMR (600 MHz, methanol-d_4) spectrum of complex of **1** with L-lysine methyl ester.

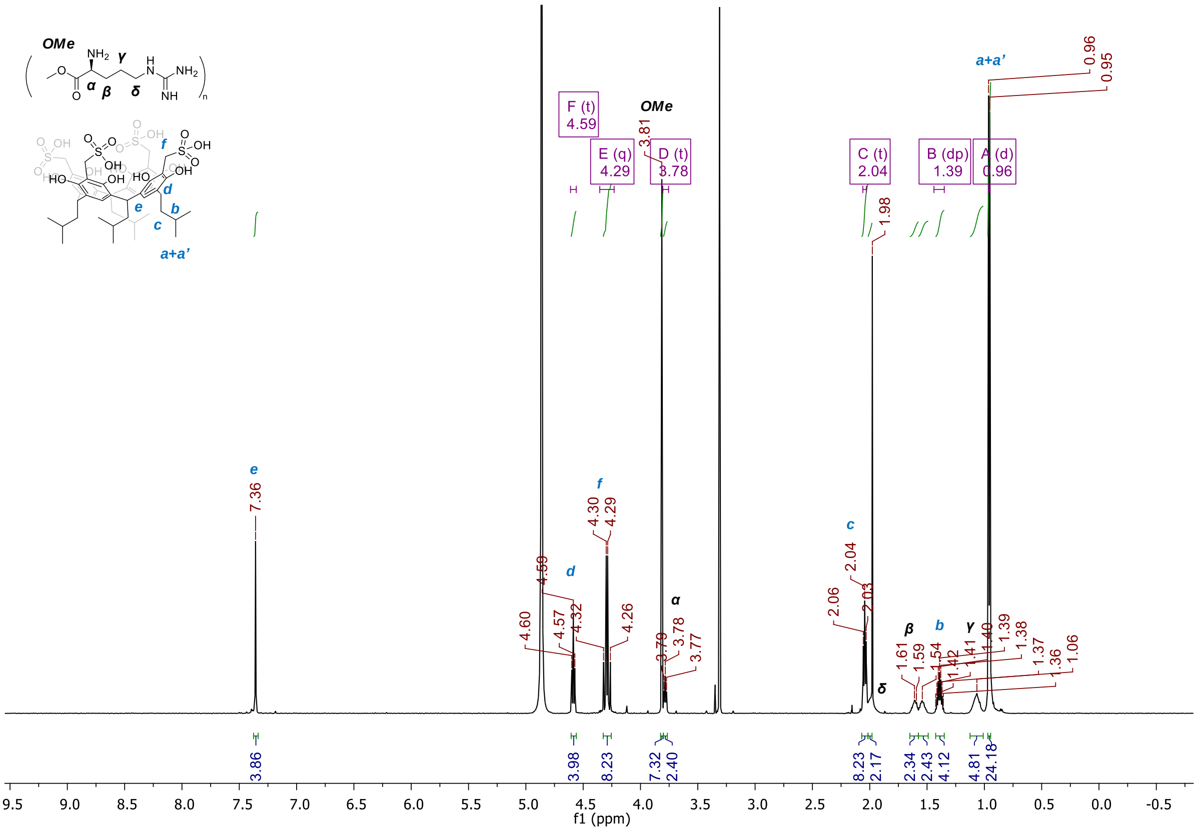

Figure S12. ^{1}H NMR (600 MHz, methanol-d_4) spectrum of complex of **1** with L-arginine methyl ester.

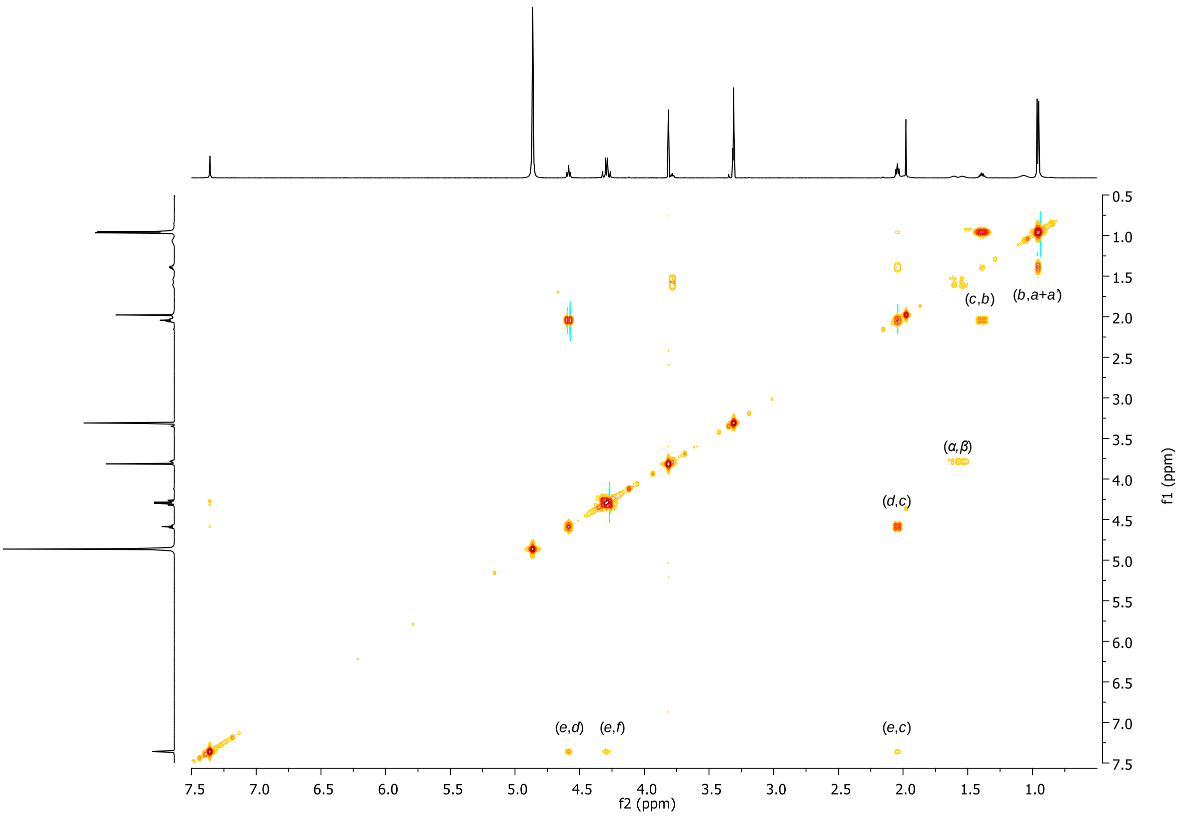

Figure S13. COSY NMR (600 MHz, methanol-d_4) spectrum of complex of **1** with L-arginine methyl ester.

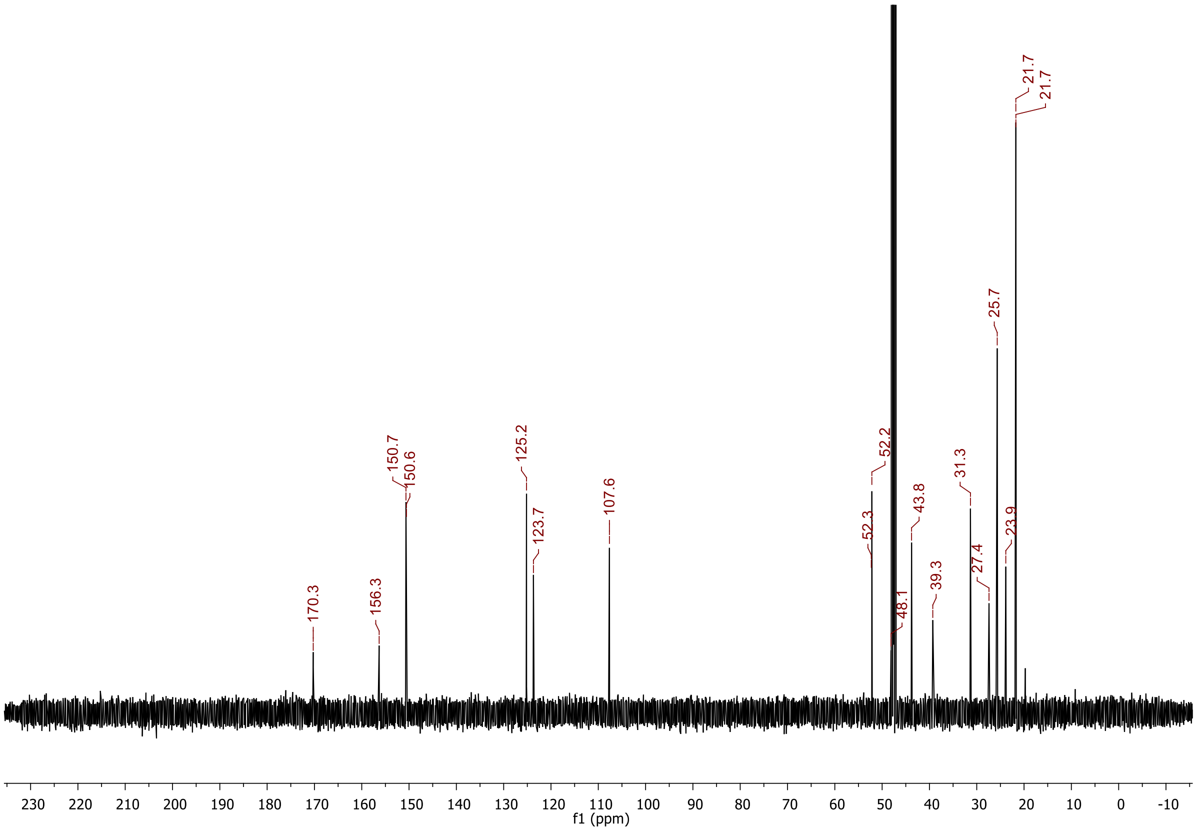

Figure S14. ^{13}C NMR (150 MHz, methanol-d_4) spectrum of complex of **1** with L-arginine methyl ester.

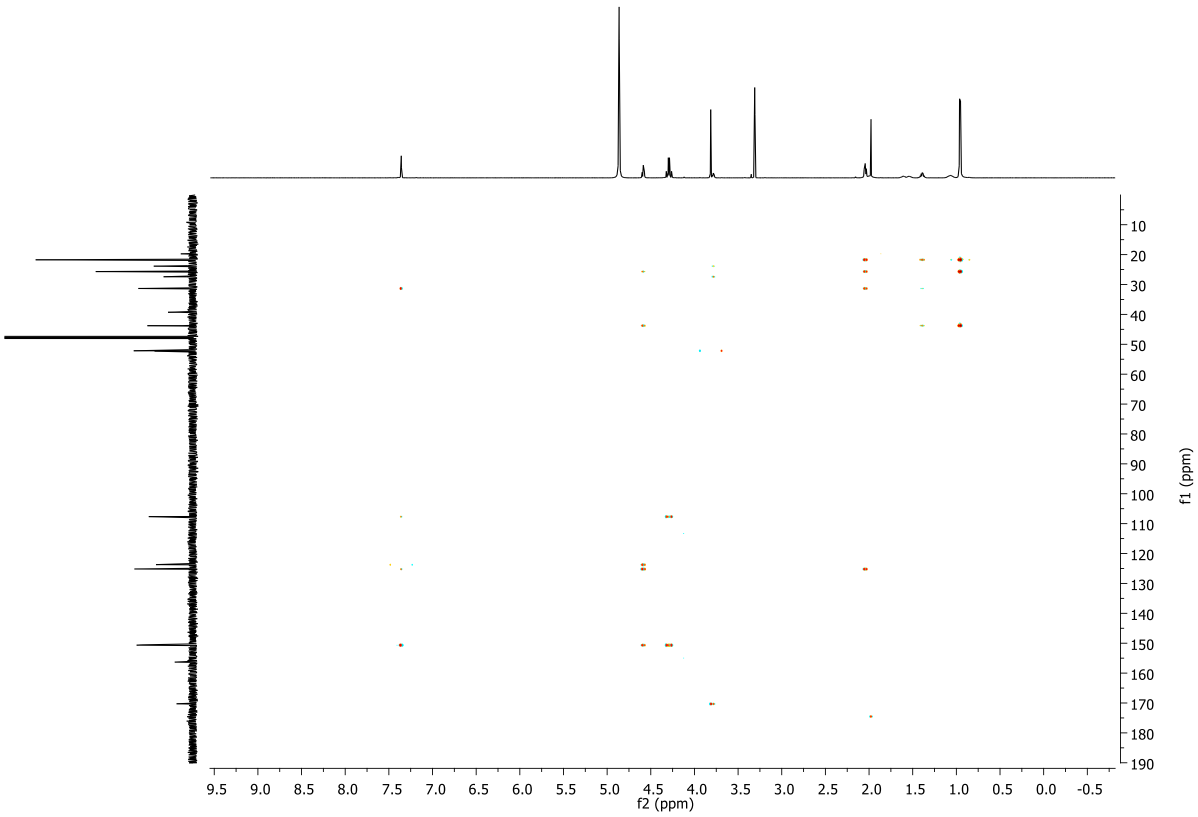

Figure S15. HMBC NMR (600 MHz, methanol-d_4) spectrum of complex of **1** with L-arginine methyl ester.

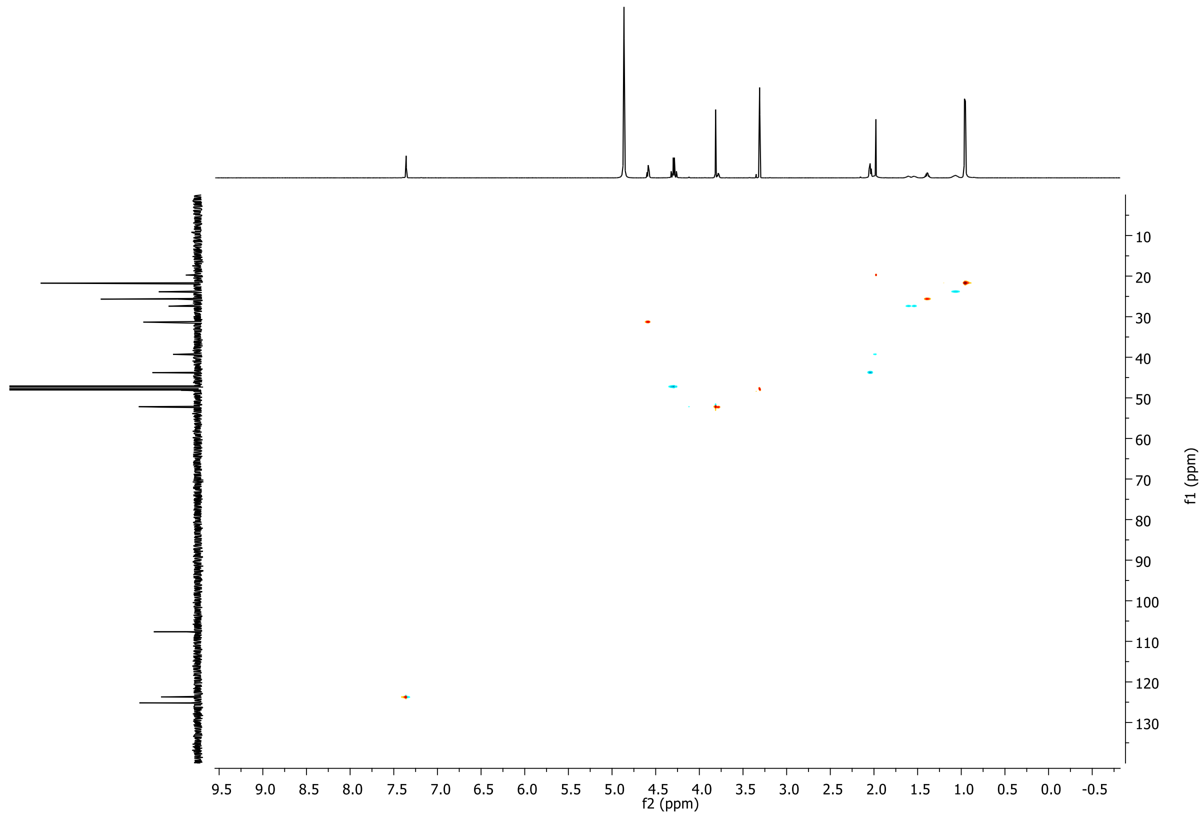

Figure S16. HSQC NMR (600 MHz, methanol-d_4) spectrum of complex of **1** with L-arginine methyl ester.

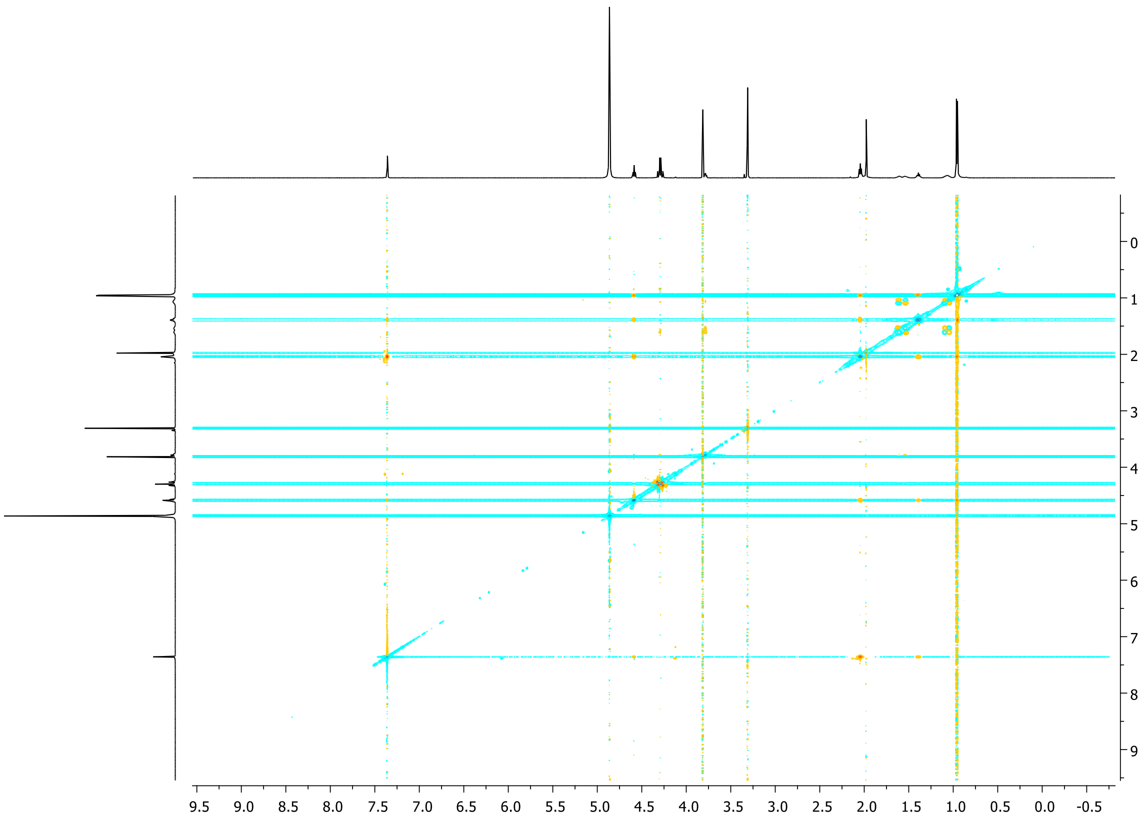

Figure S17. ROESY NMR (600 MHz, methanol-d_4) spectrum of complex of **1** with L-arginine methyl ester.

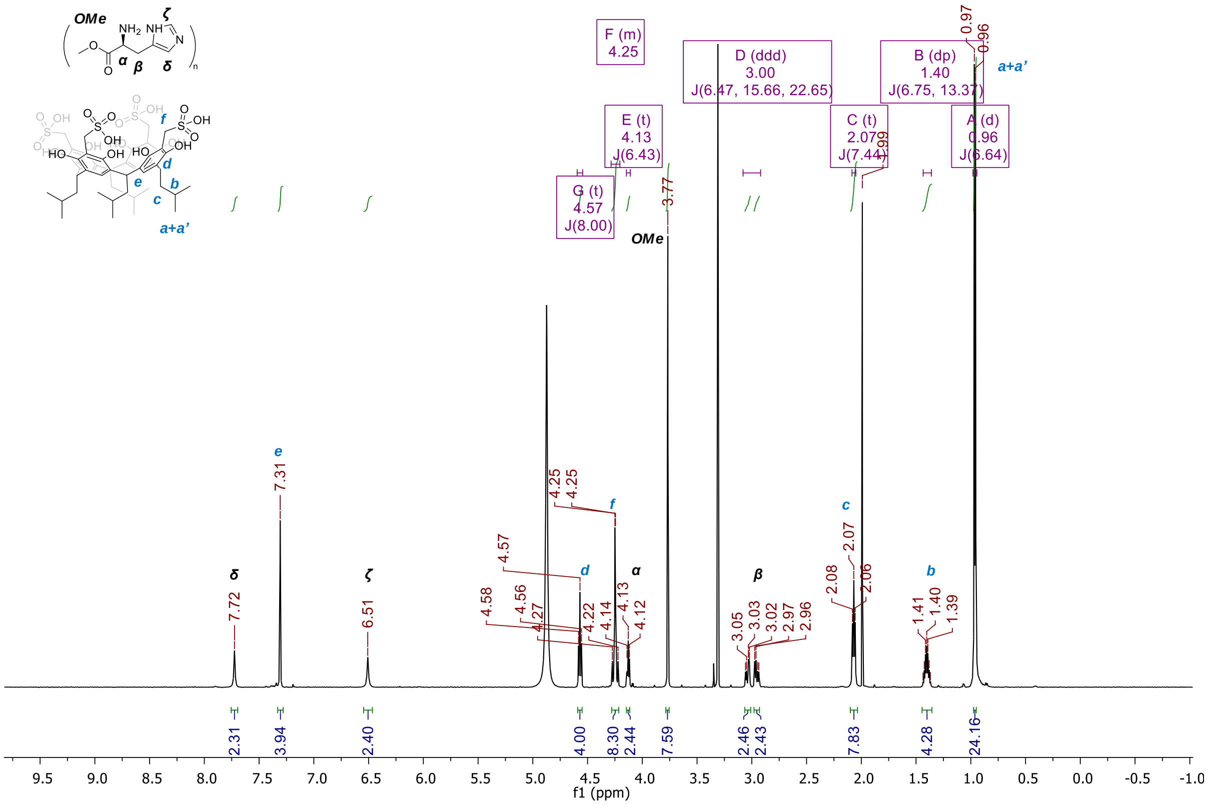

Figure S18. ^{1}H NMR (600 MHz, methanol-d_4) spectrum of complex of **1** with L-histidine methyl ester.

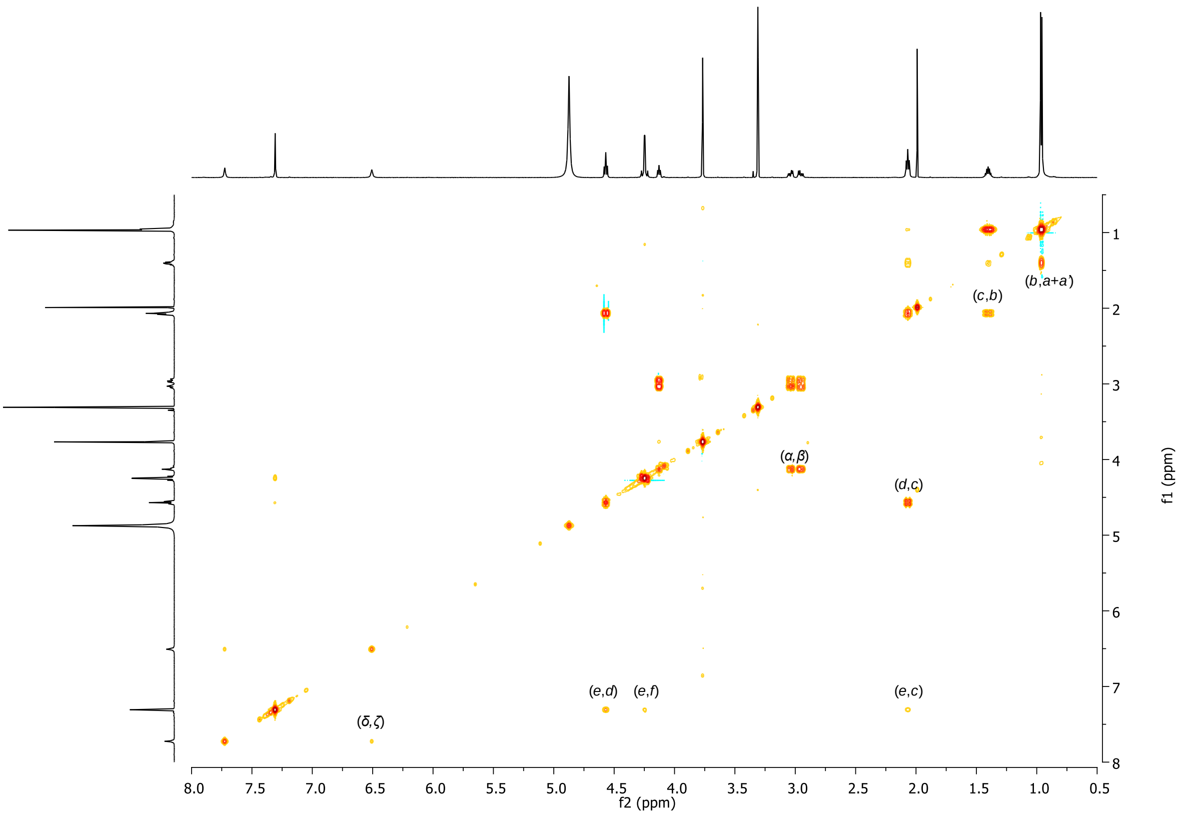

Figure S19. COSY NMR (600 MHz, methanol-d_4) spectrum of complex of **1** with L-histidine methyl ester.

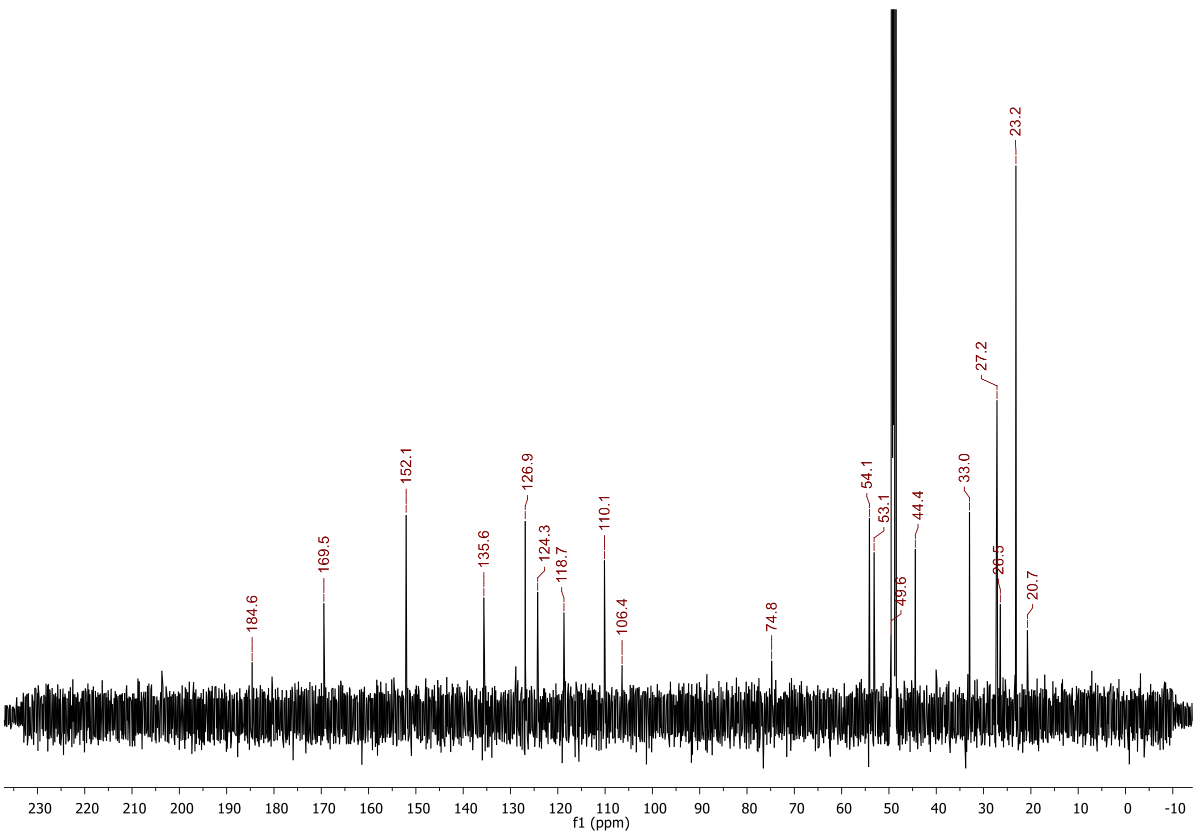

Figure S20. ^{13}C NMR (150 MHz, methanol-d_4) spectrum of complex of **1** with L-histidine methyl ester.

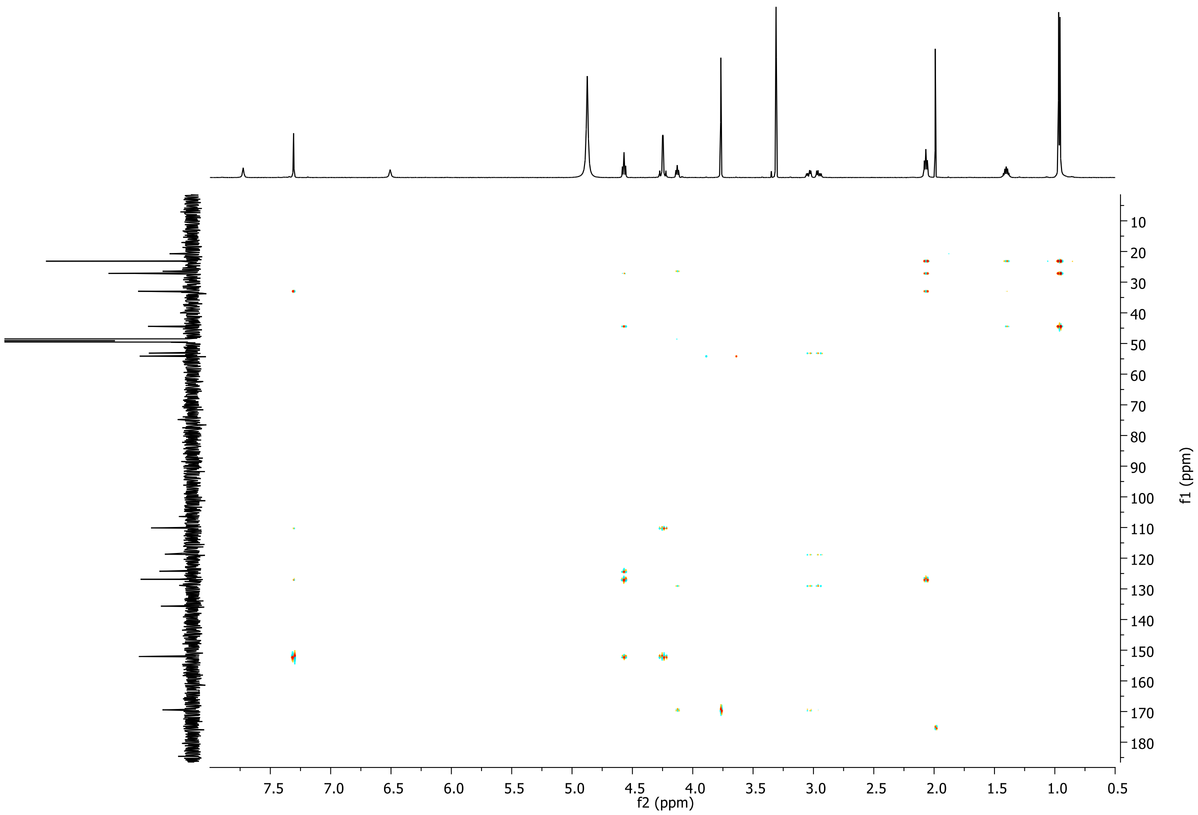

Figure S21. HMBC NMR (600 MHz, methanol-d_4) spectrum of complex of **1** with L-histidine methyl ester.

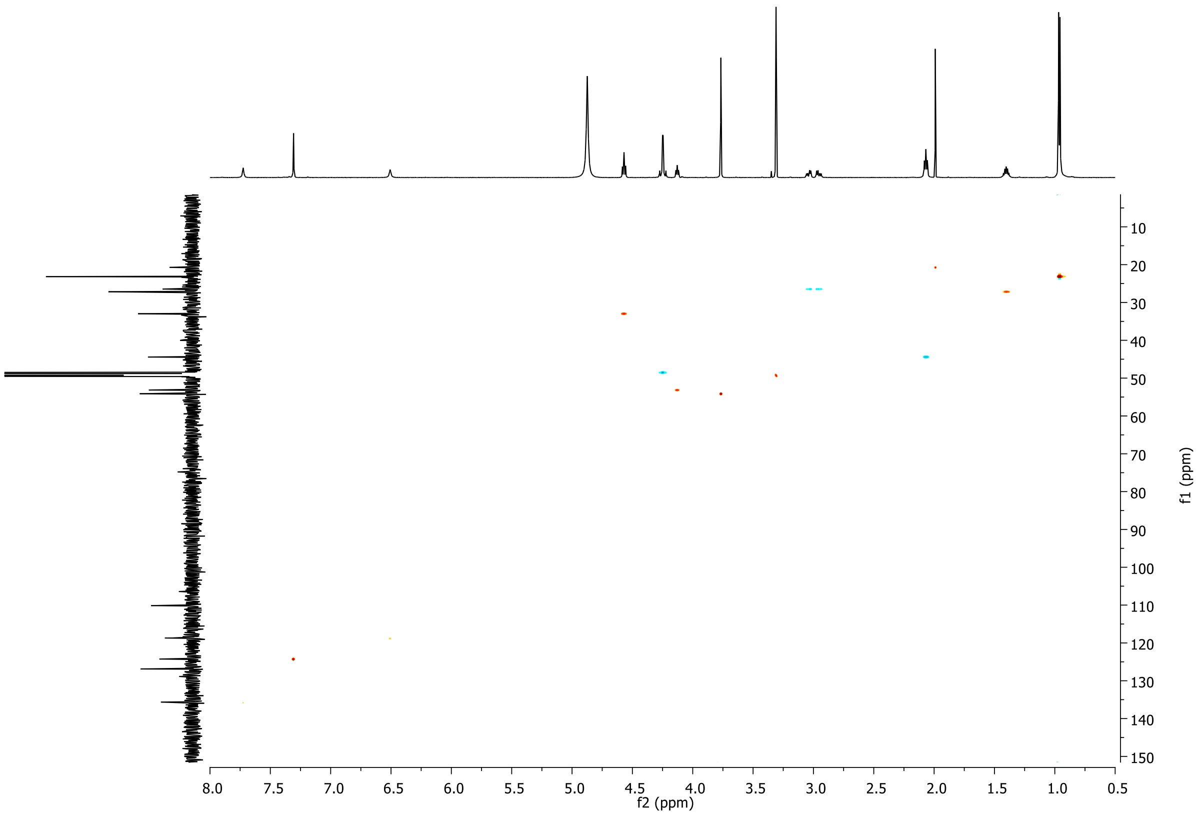

Figure S22. HSQC NMR (600 MHz, methanol-d_4) spectrum of complex of **1** with L-histidine methyl ester.

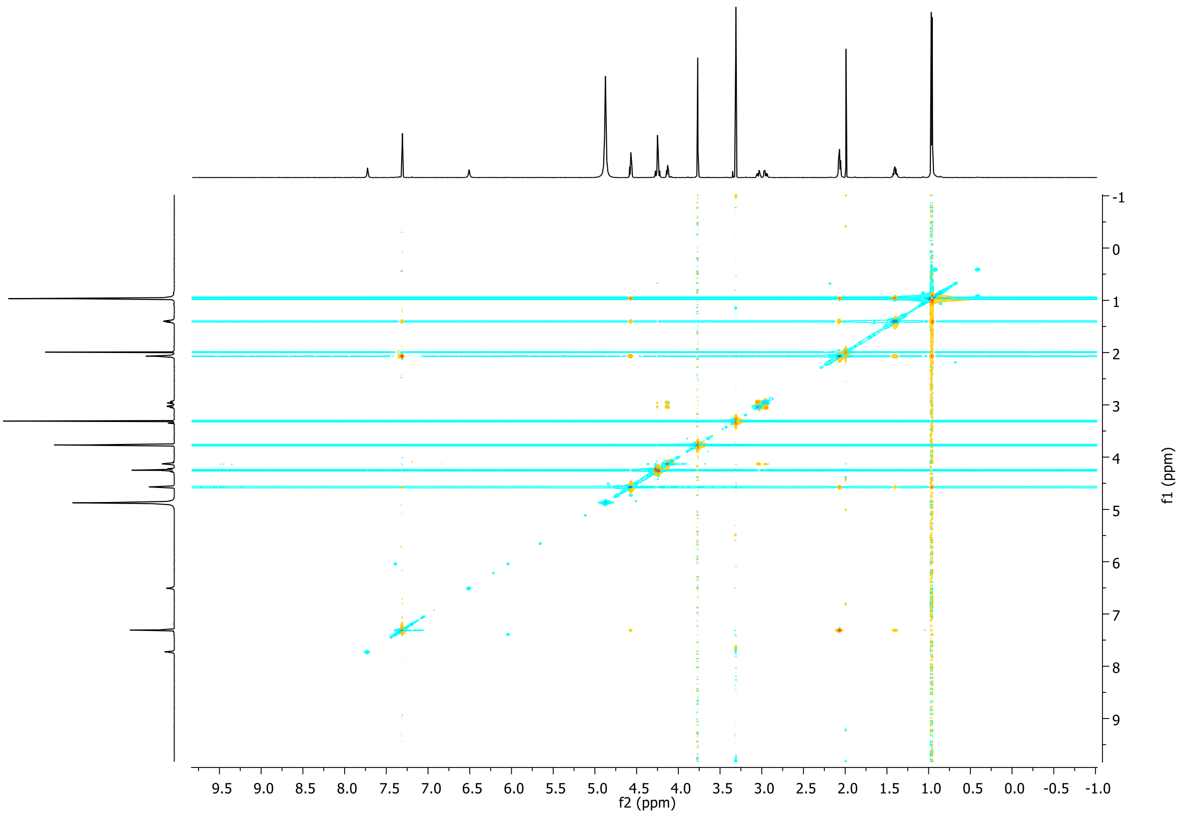

Figure S23. ROESY NMR (600 MHz, methanol-d_4) spectrum of complex of **1** with L-histidine methyl ester.

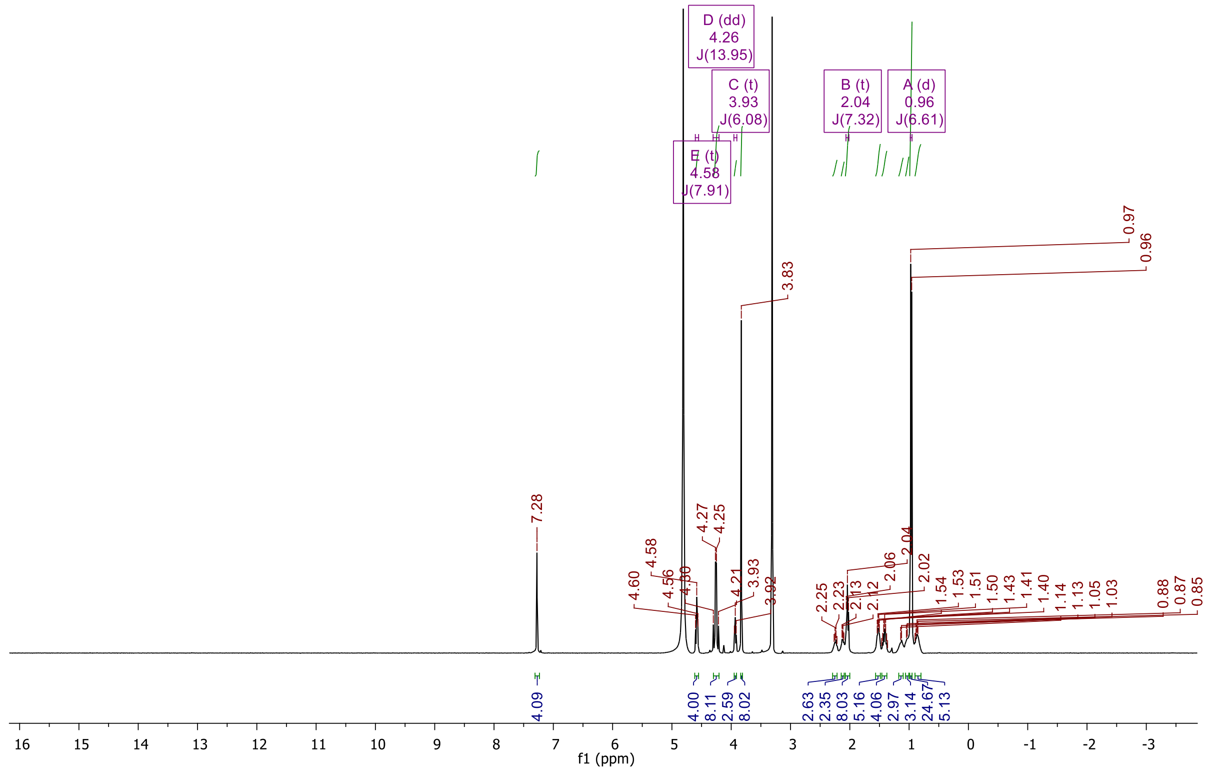

Figure S24. ^{1}H NMR (400 MHz, methanol-d_4) spectrum of complex of **1** with D-lysine methyl ester.

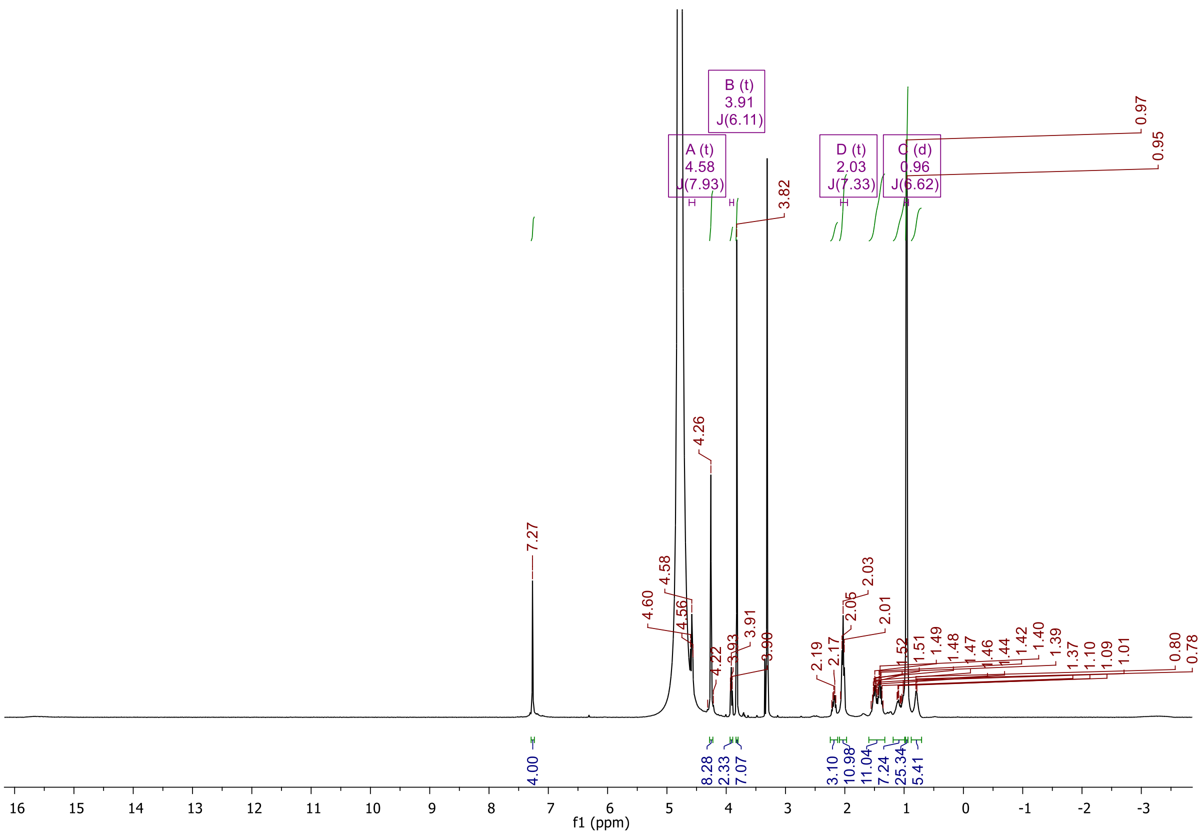

Figure S25. ^{1}H NMR (400 MHz, methanol-d_4) spectrum of complex of **1** with (*rac*)-lysine methyl ester.

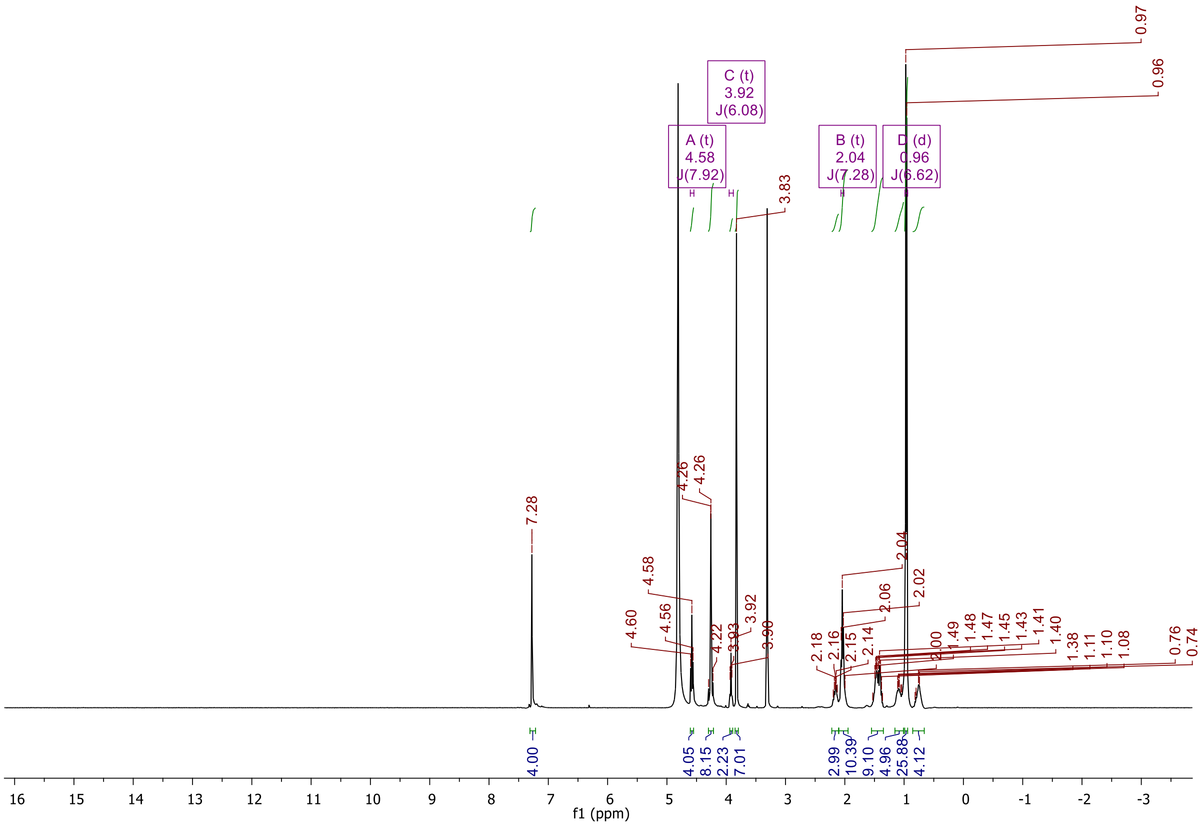

Figure S26. ^{1}H NMR (400 MHz, methanol-d_4) spectrum of complex of **1** with L- and (*rac*)-lysine methyl ester.

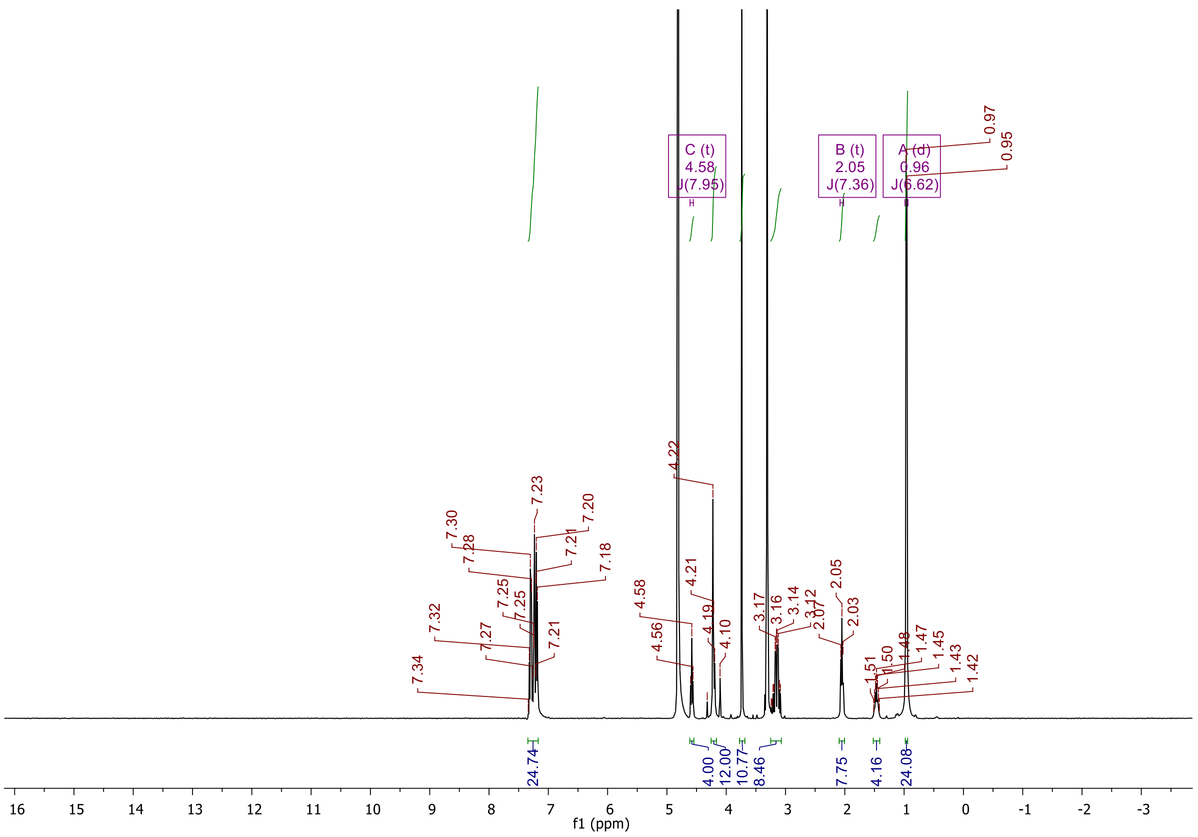

Figure S27. ^{1}H NMR (400 MHz, methanol-d_4) spectrum of complex of **1** with L-phenylalanine methyl ester.

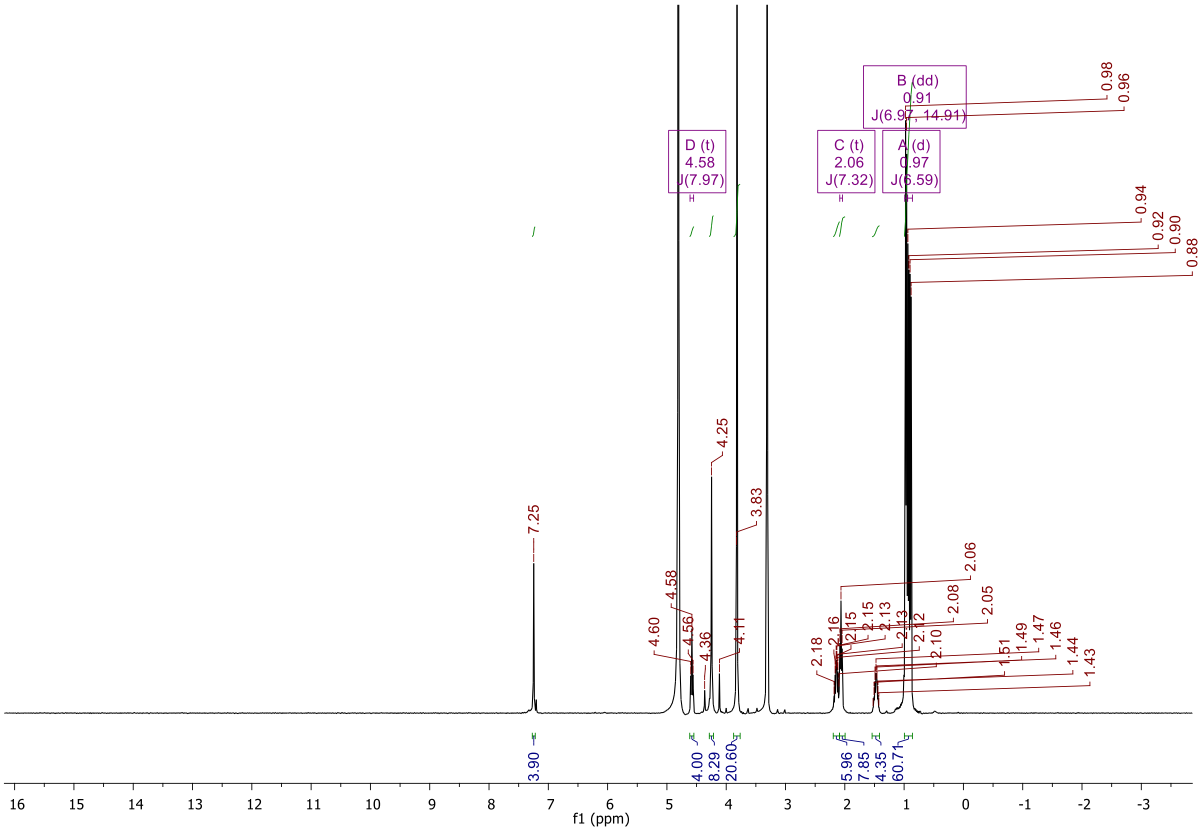

Figure S28. ^{1}H NMR (400 MHz, methanol-d_4) spectrum of complex of **1** with L-valine methyl ester.

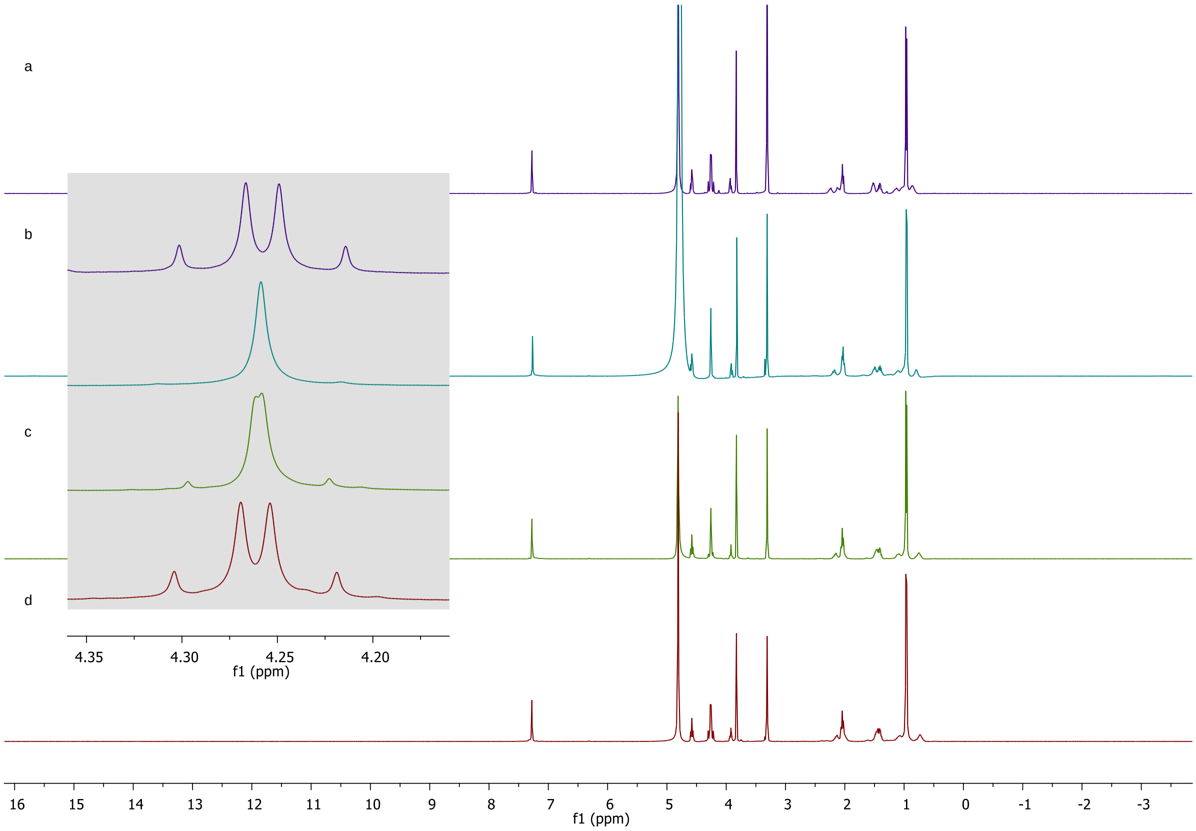

Figure S29. ^{1}H NMR (400 MHz, methanol-d_4) spectra of complexes of **1** with (a) L–lysine methyl ester; (b) (*rac*)-lysine methyl ester; (c) mixture of L- and (*rac*)-lysine methyl ester and (d) D–lysine methyl ester. Inset (grey) depict *f* protons of **1**.

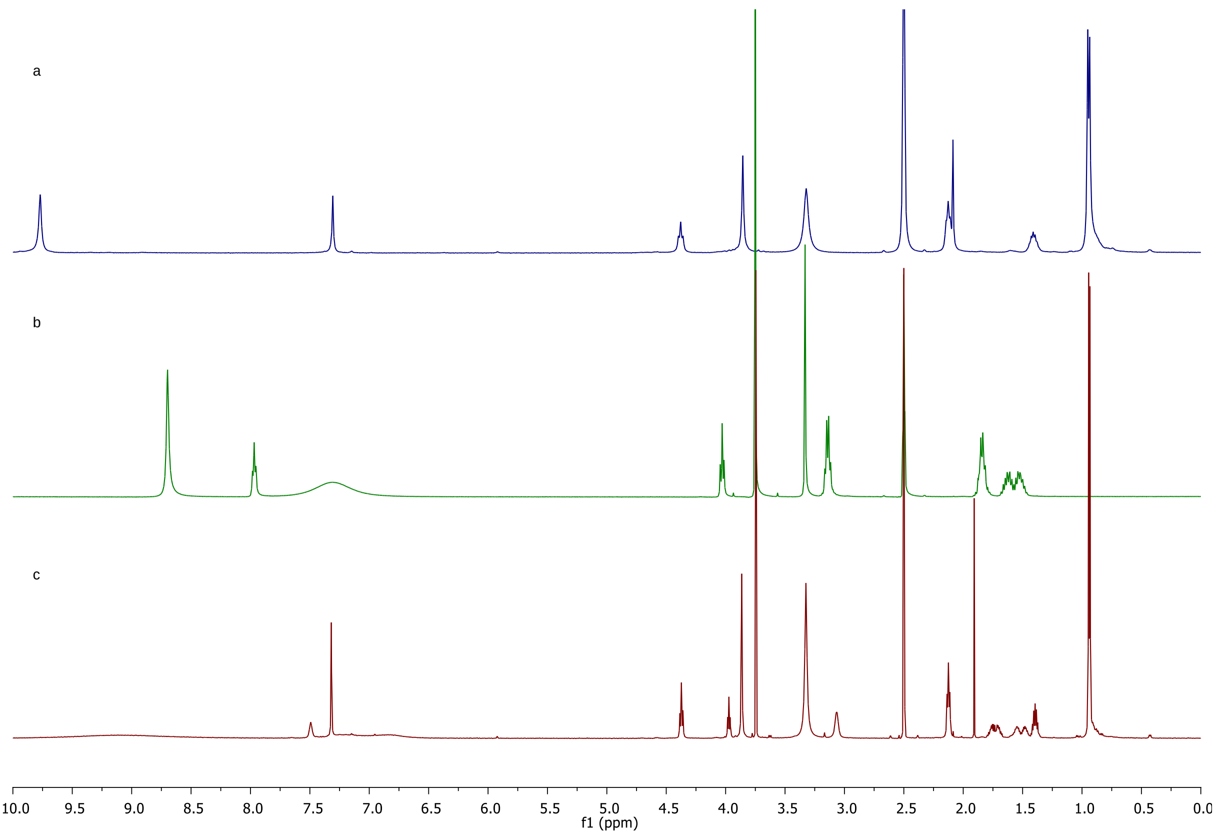

Figure S30. ^{1}H NMR (400 MHz and 600MHz, DMSO-d_6) spectra of (a) **1**; (b) L-arginine methyl ester; (c) **1**(ArgOMe)$_2$.

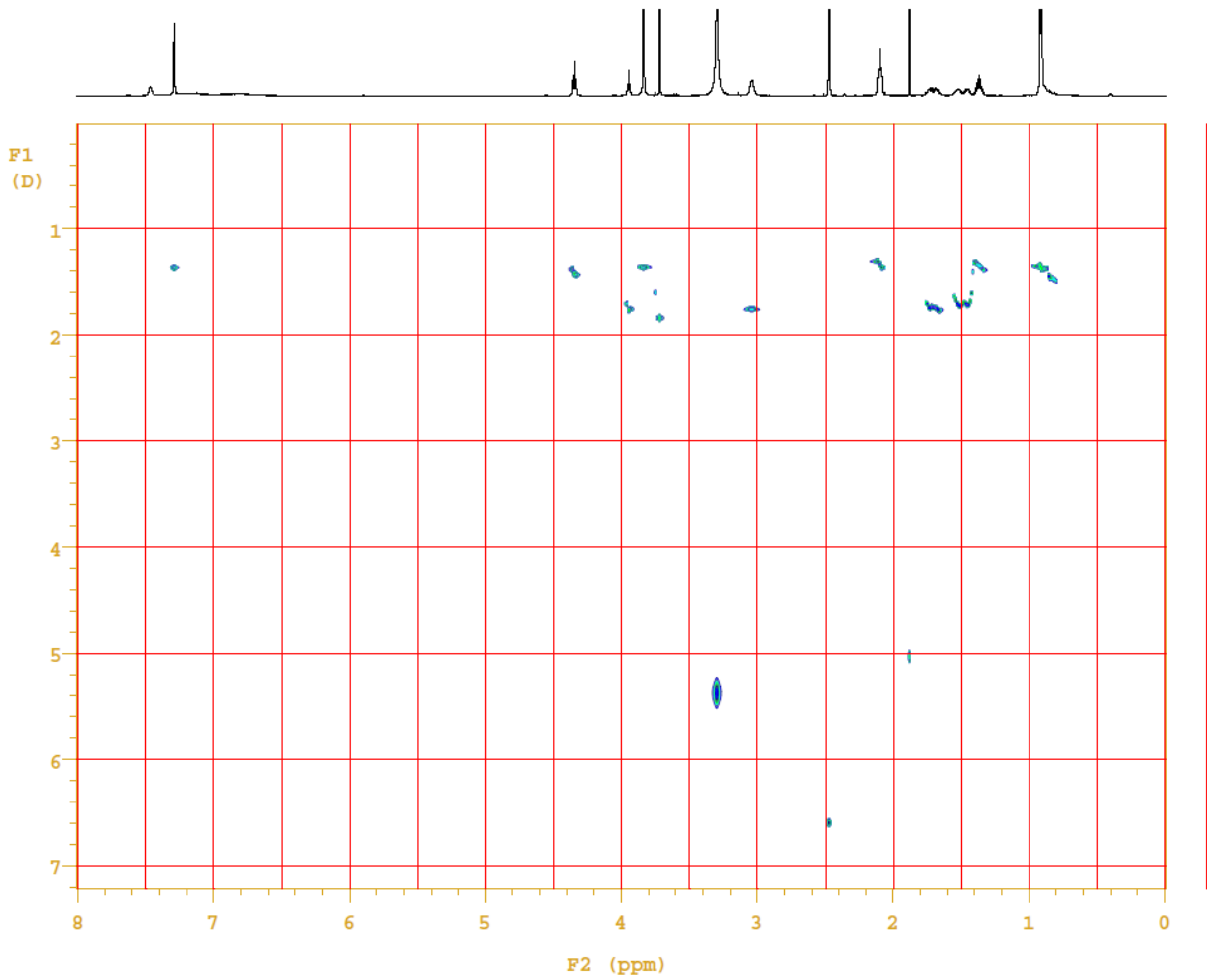

Figure S31. DOSY NMR spectrum of **1**(ArgOMe)$_2$ DMSO-d_6, at 298K, 600 MHz.

.

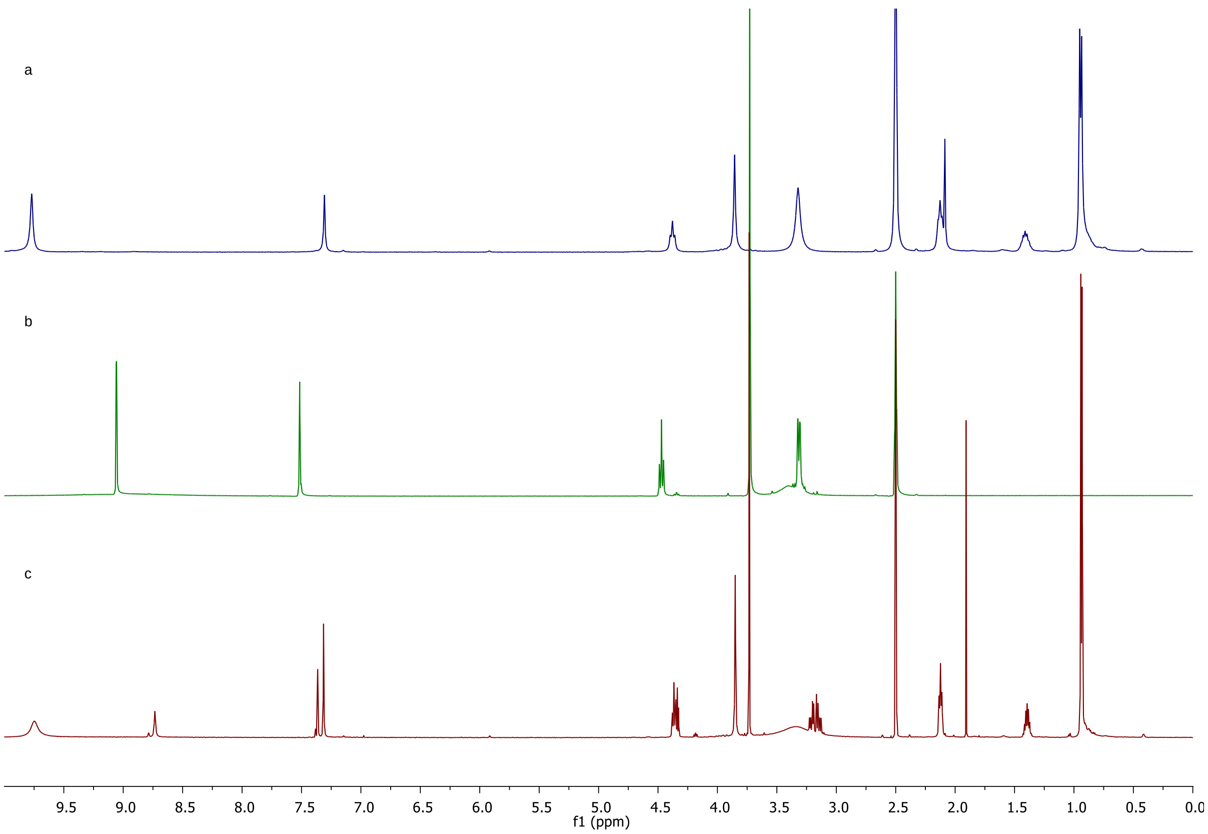

Figure S32. ^{1}H NMR (400 MHz and 600MHz, DMSO-d_6) spectra of (a) **1**; (b) L-histidine methyl ester; (c) **1**(HisOMe)$_2$.

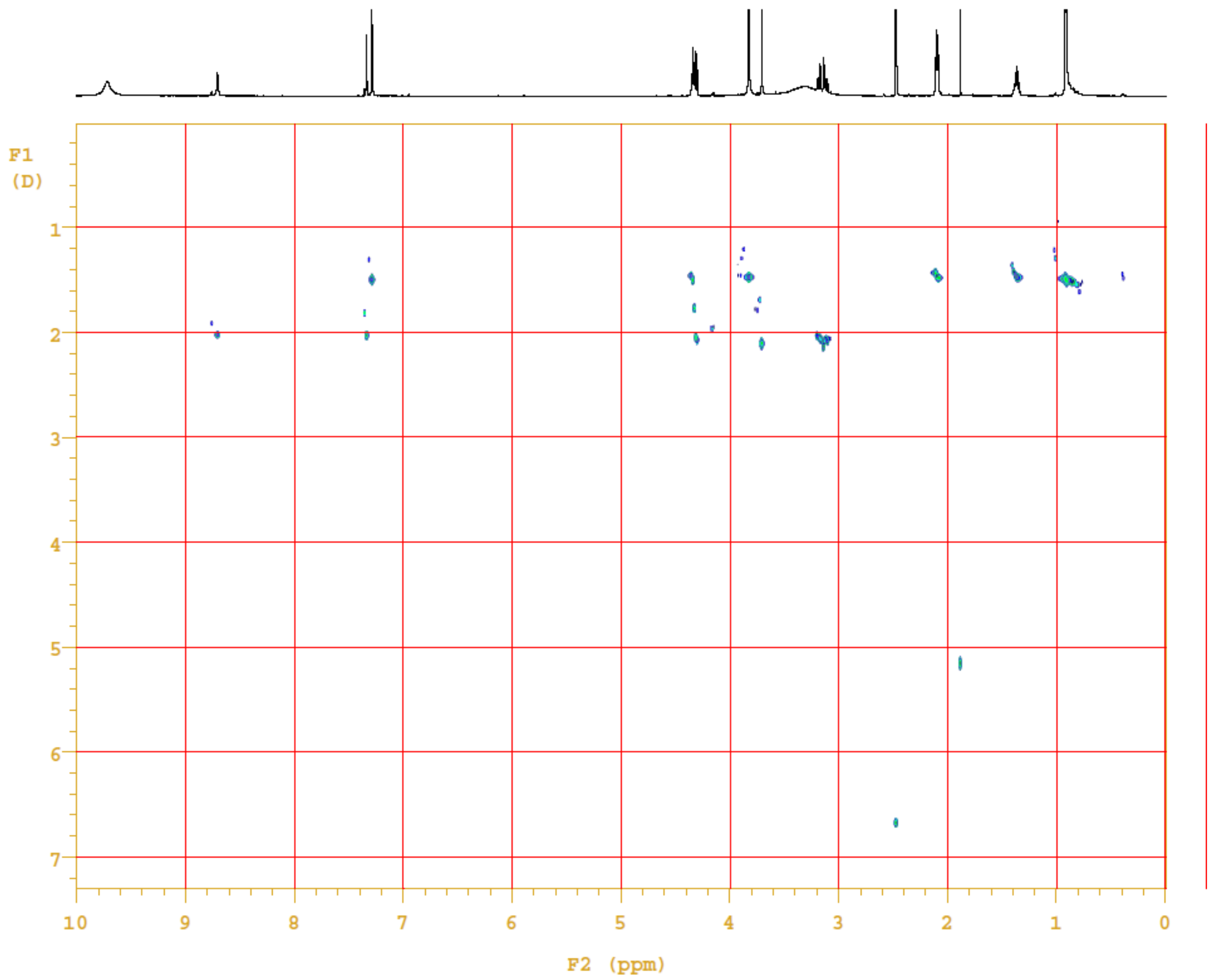

Figure S33. DOSY NMR spectrum of **1**(HisOMe)$_2$ DMSO-d_6, at 298K, 600 MHz.

1.3. NMR spectra of chiral cavitands

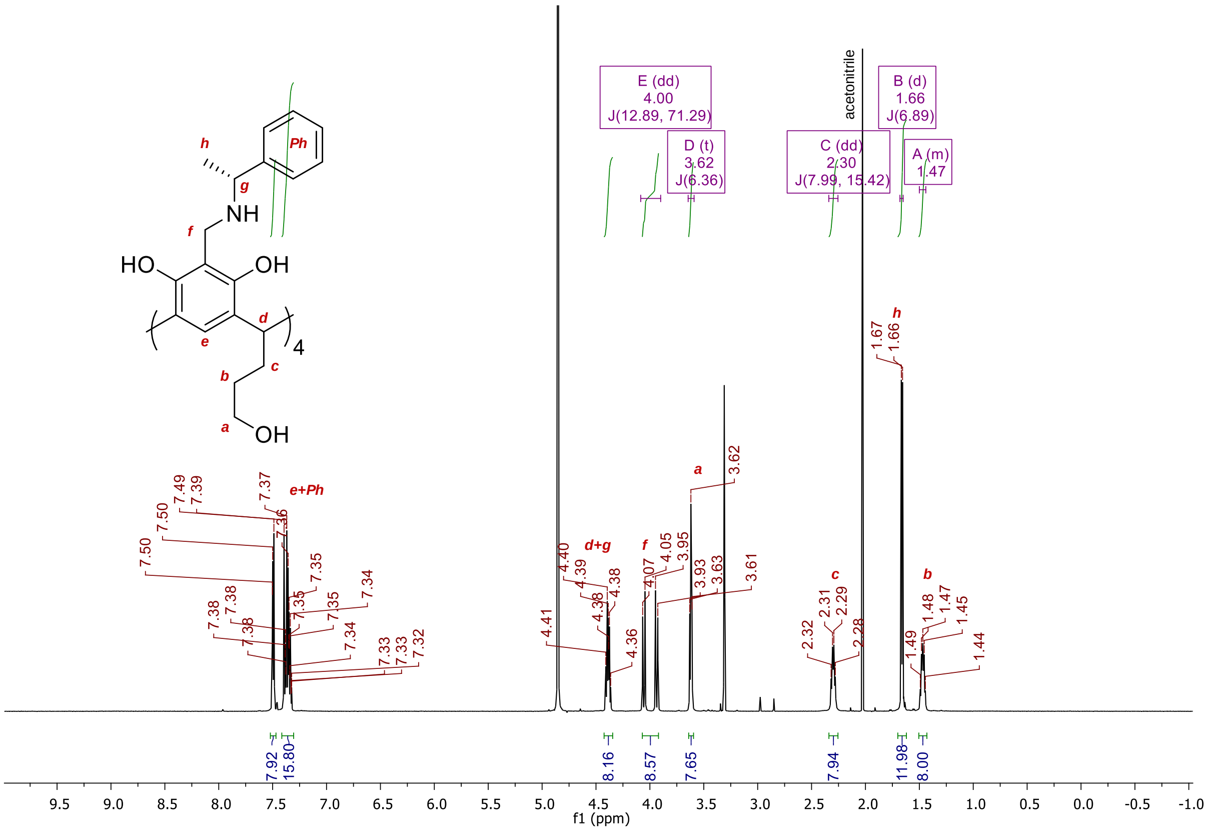

Figure S34. ^{1}H NMR (600 MHz, methanol-d_4) spectrum of (R)-**2**.

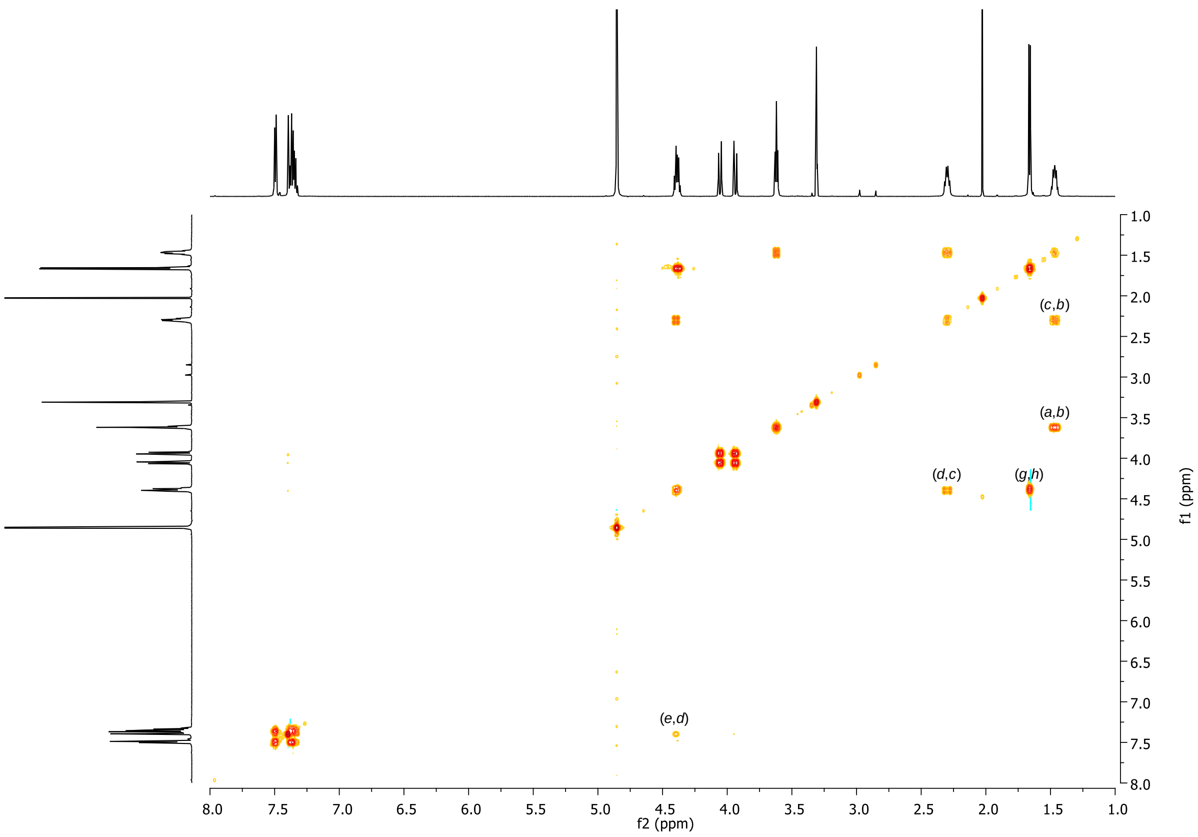

Figure S35. COSY NMR (600 MHz, methanol-d_4) spectrum of (*R*)-**2**.

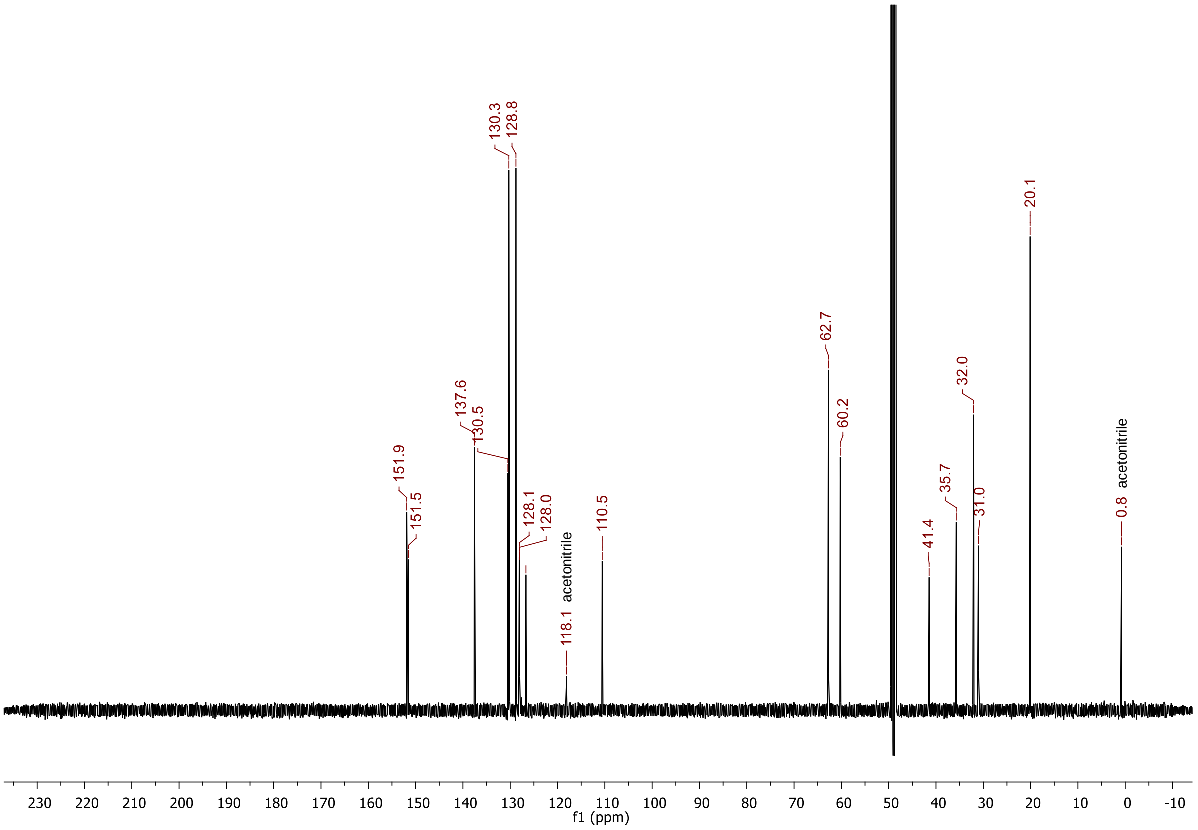

Figure S36. ^{13}C NMR (150 MHz, methanol-d_4) spectrum of (*R*)-**2**.

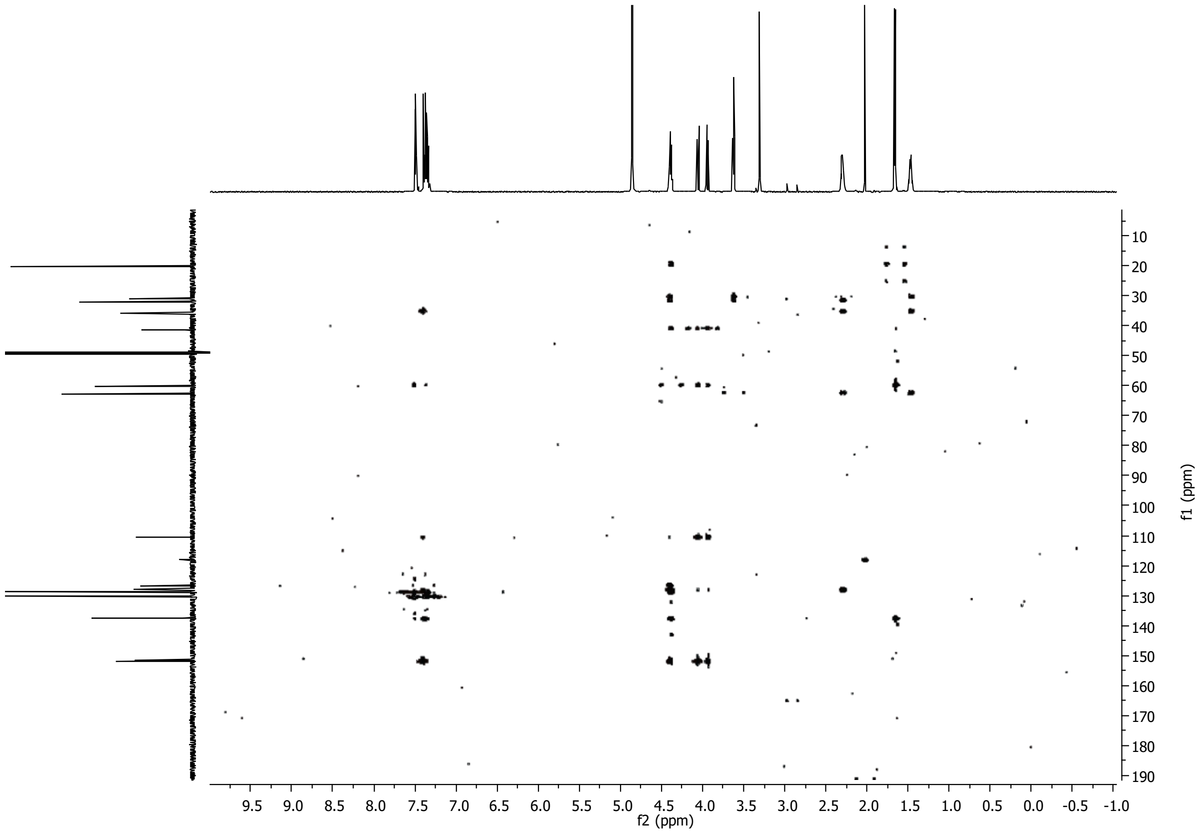

Figure S37. HMBC NMR (600 MHz, methanol-d_4) spectrum of (R)-**2**.

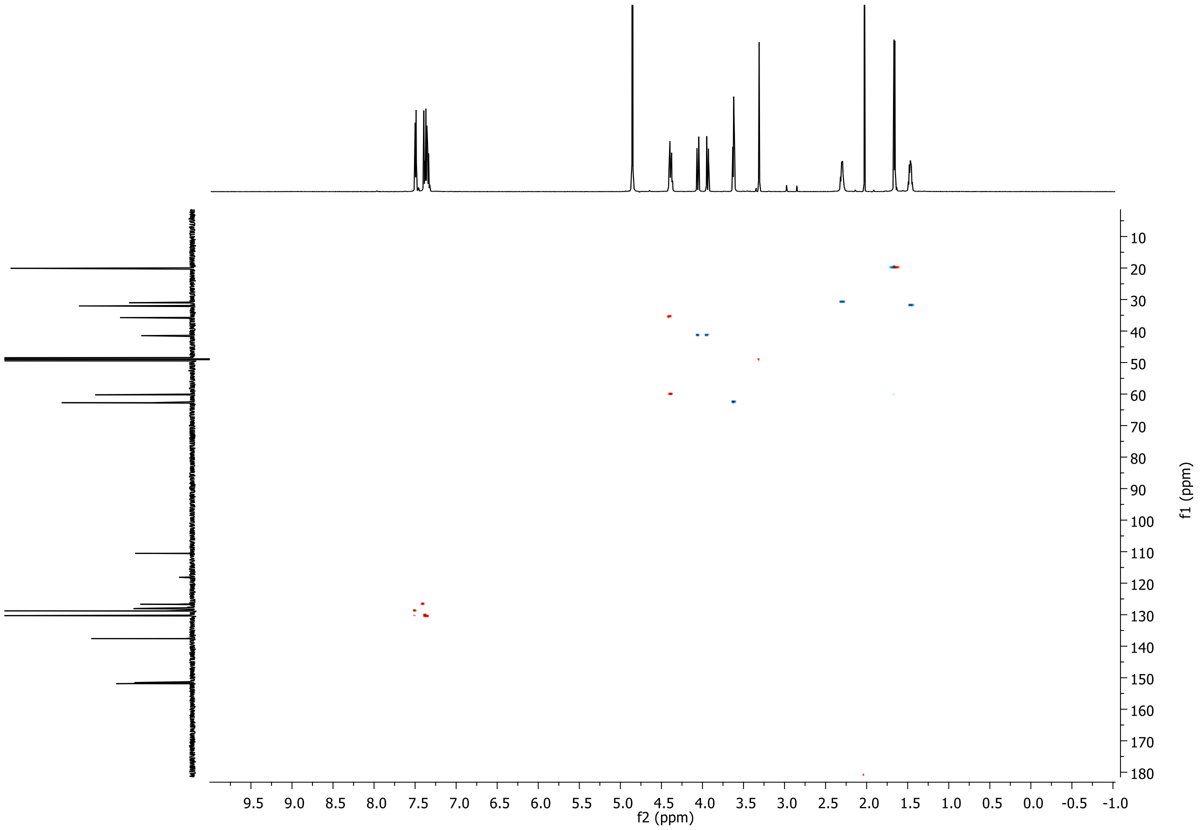

Figure S38. HSQC NMR (600 MHz, methanol-d_4) spectrum of (R)-**2**.

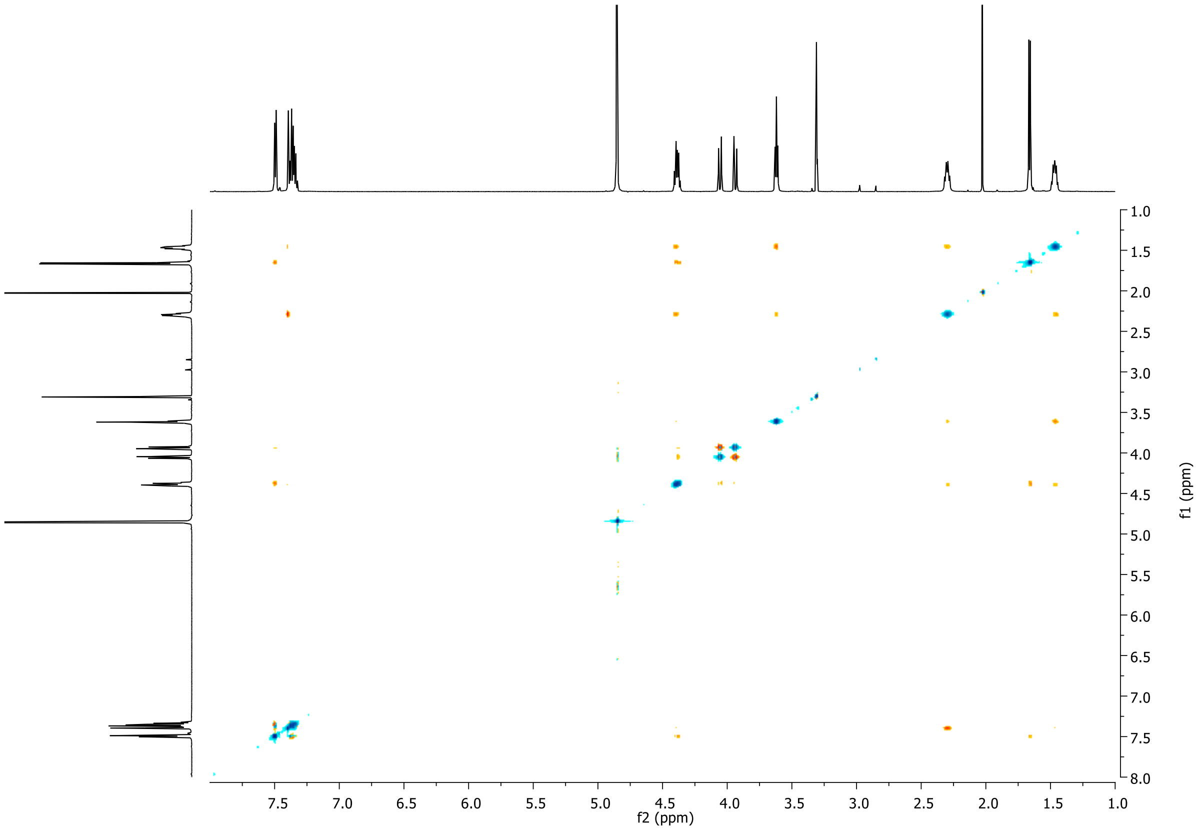

Figure S39. ROESY NMR (600 MHz, methanol-d_4) spectrum of (*R*)-**2**.

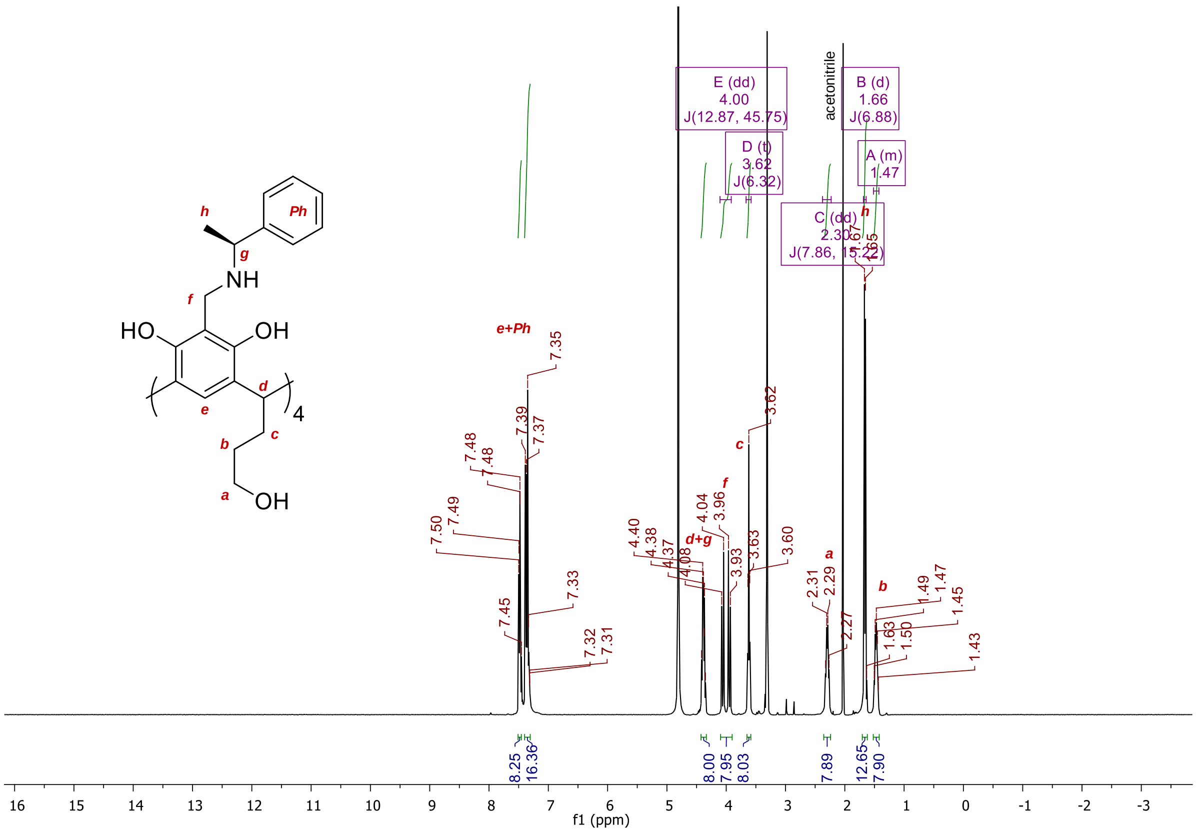

Figure S40. ^{1}H NMR (400 MHz, methanol-d_4) spectrum of (*S*)-**2**.

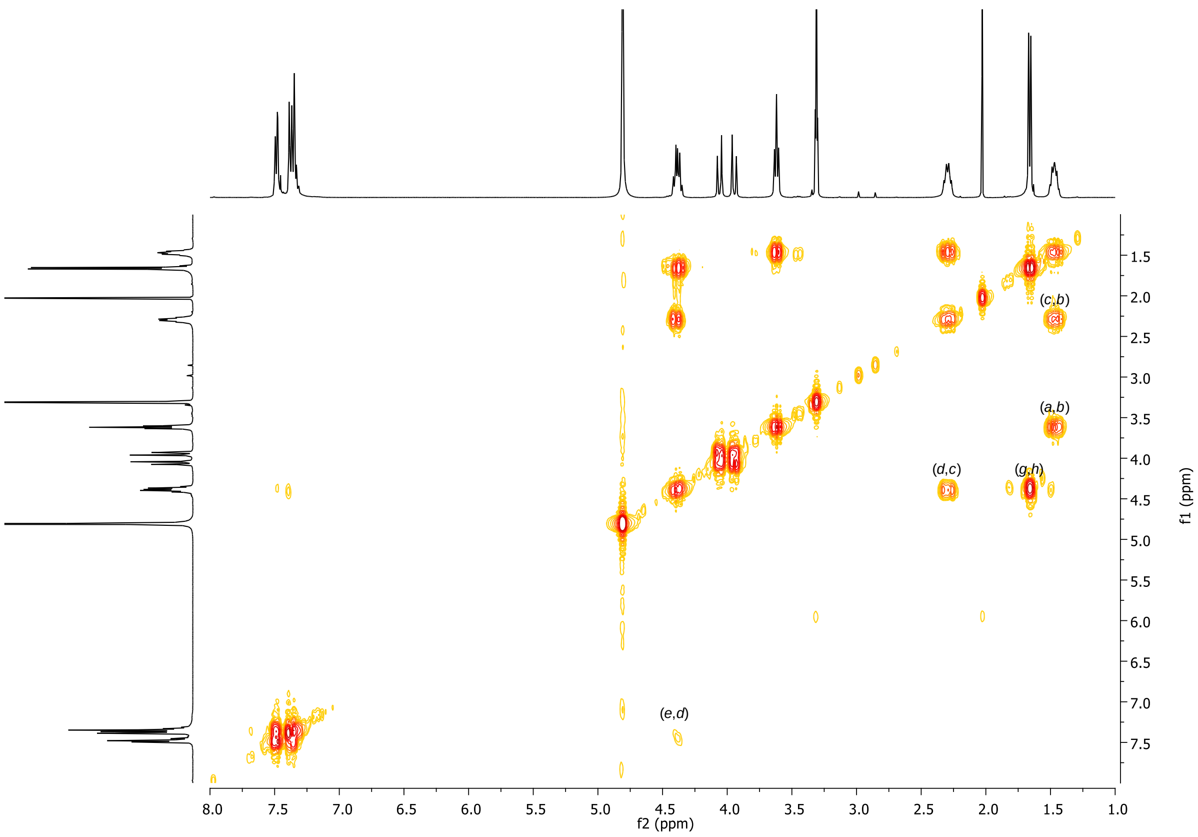

Figure S41 COSY NMR (400 MHz, methanol-d_4) spectrum of (*S*)-**2**.

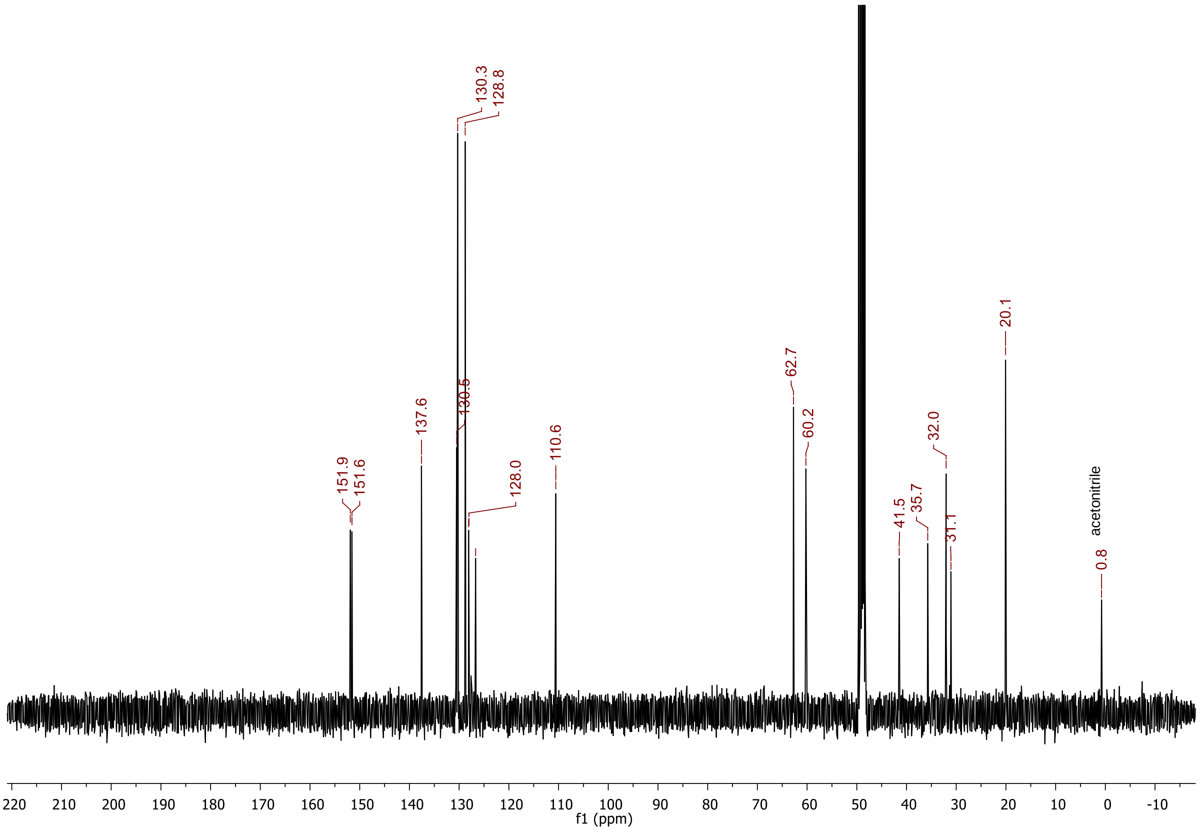

Figure S42. ^{13}C NMR (100 MHz, methanol-d_4) spectrum of (*S*)-**2**.

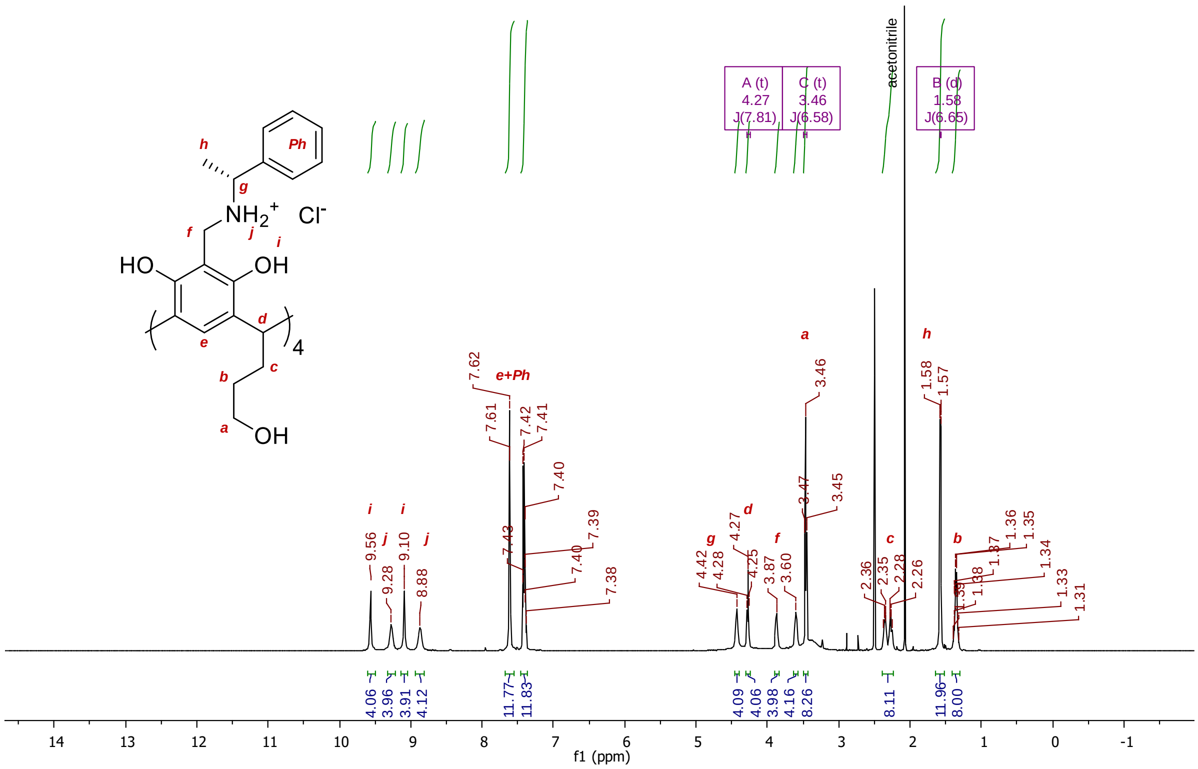

Figure S43. ^{1}H NMR (600 MHz, DMSO-d_6) spectrum of (*R*)-**2**.

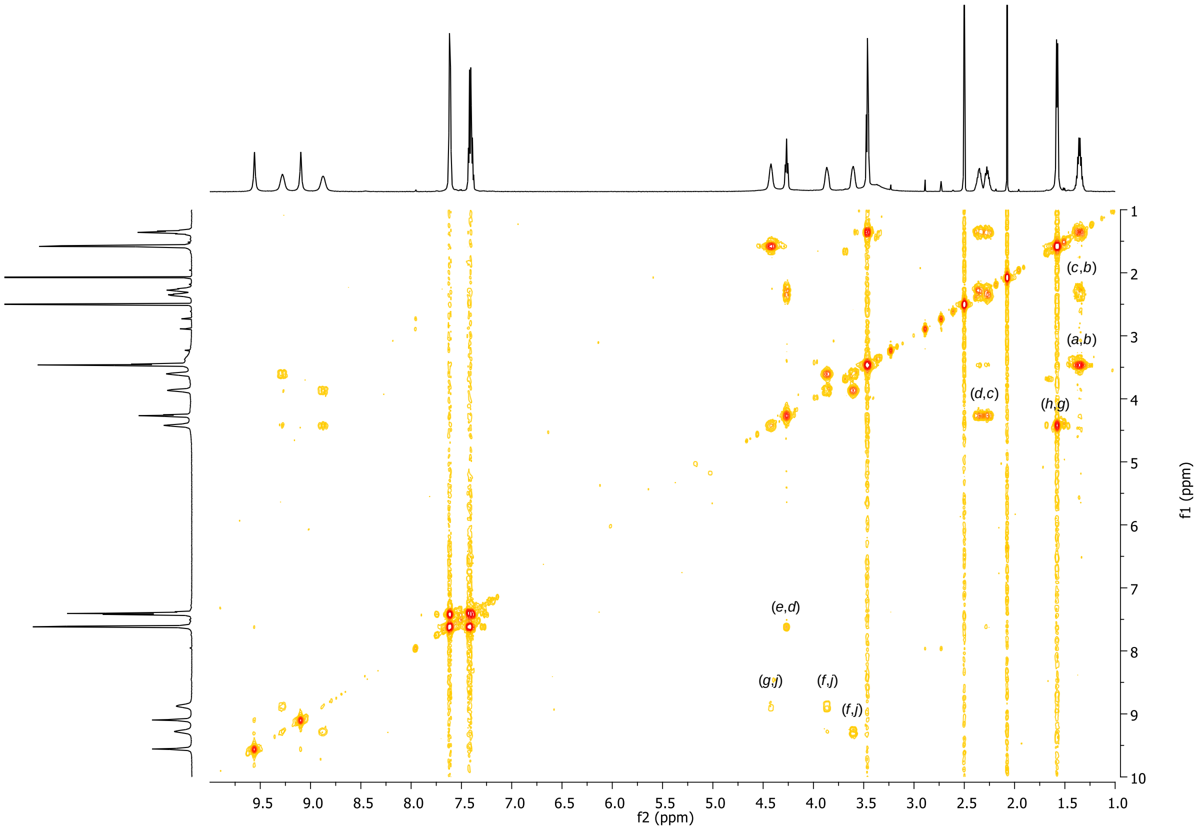

Figure S44. COSY NMR (600 MHz, DMSO-d_6) spectrum of (*R*)-**2**.

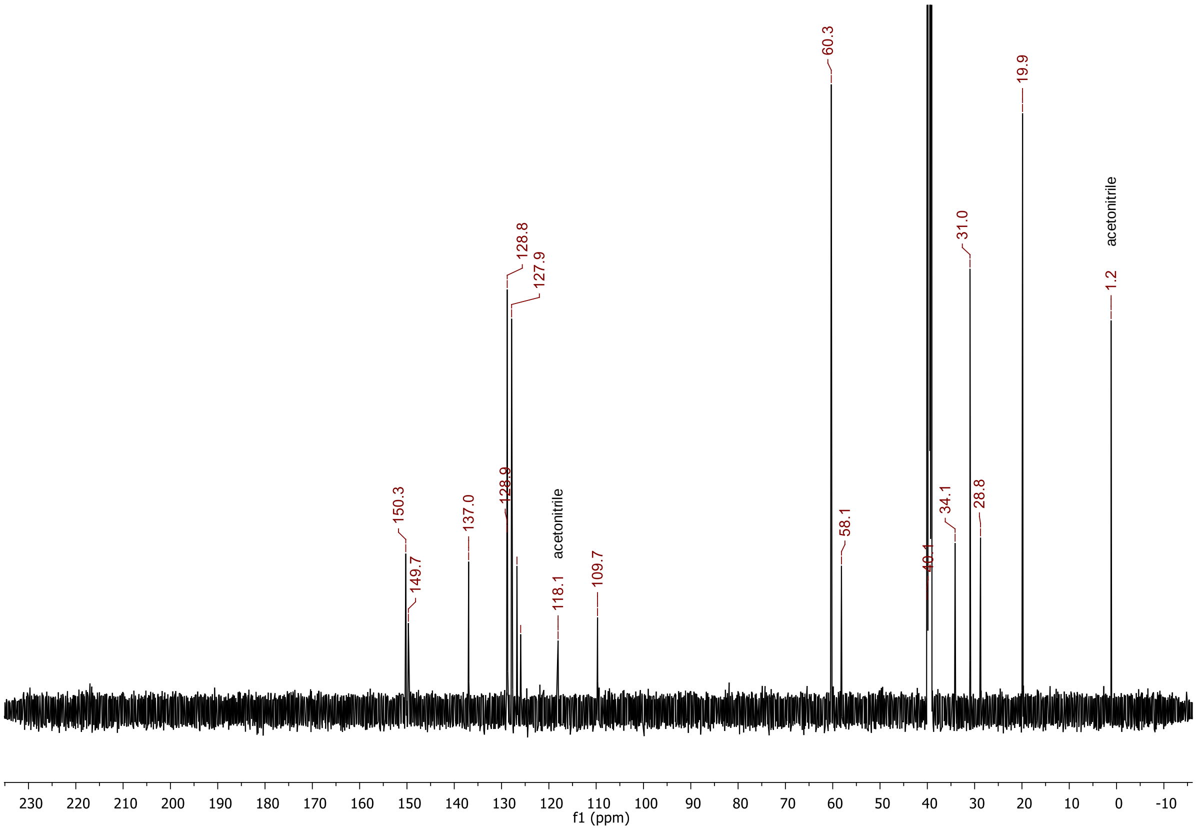

Figure S45. ^{13}C NMR (150 MHz, DMSO-d_6) spectrum of (R)-**2**.

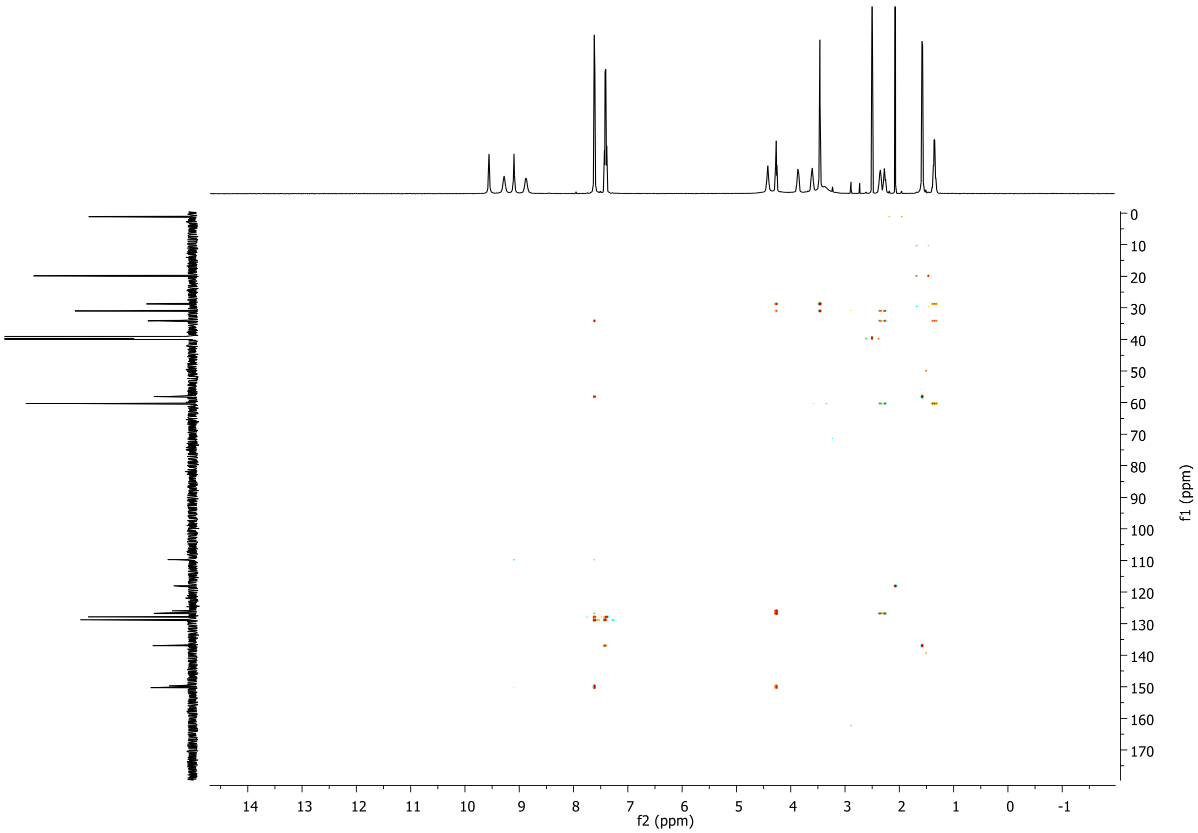

Figure S46. HMBC NMR (600 MHz, DMSO-d_6) spectrum of (R)-**2**.

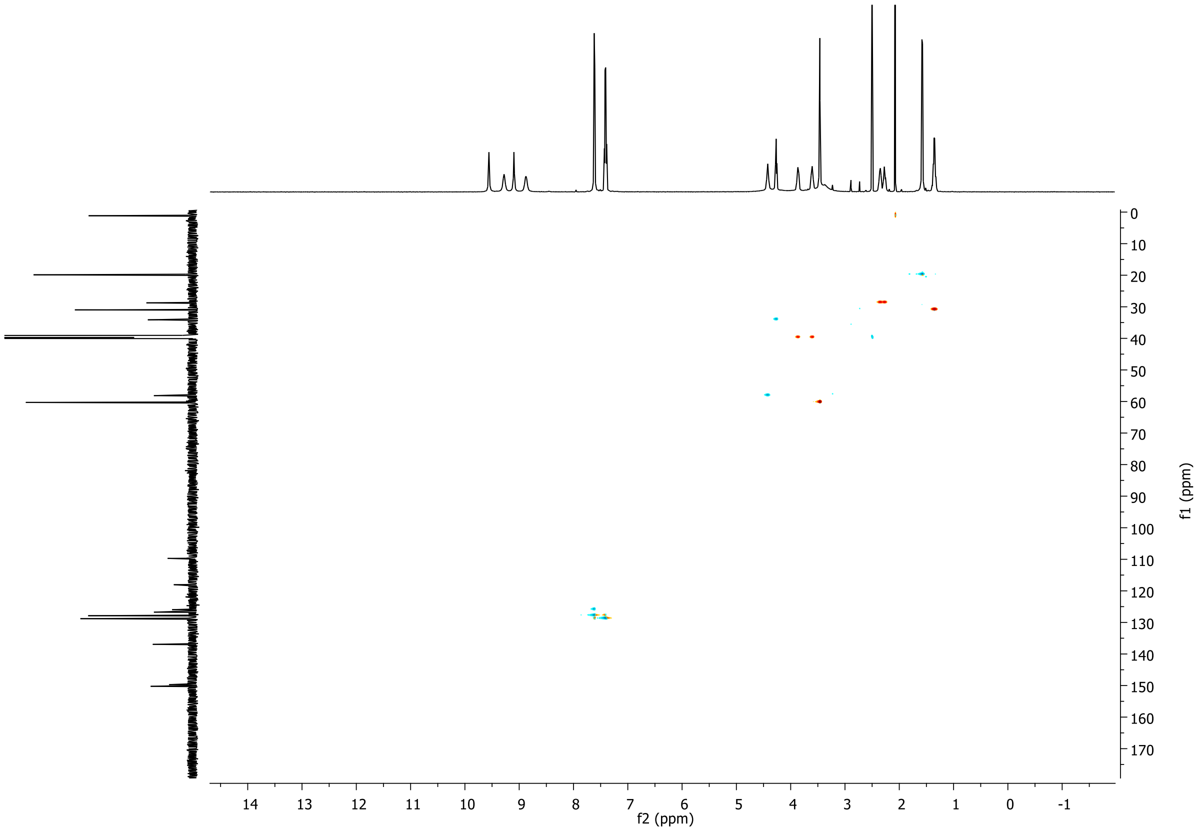

Figure S47. HSQC NMR (600 MHz, DMSO-d_6) spectrum of (*R*)-**2**.

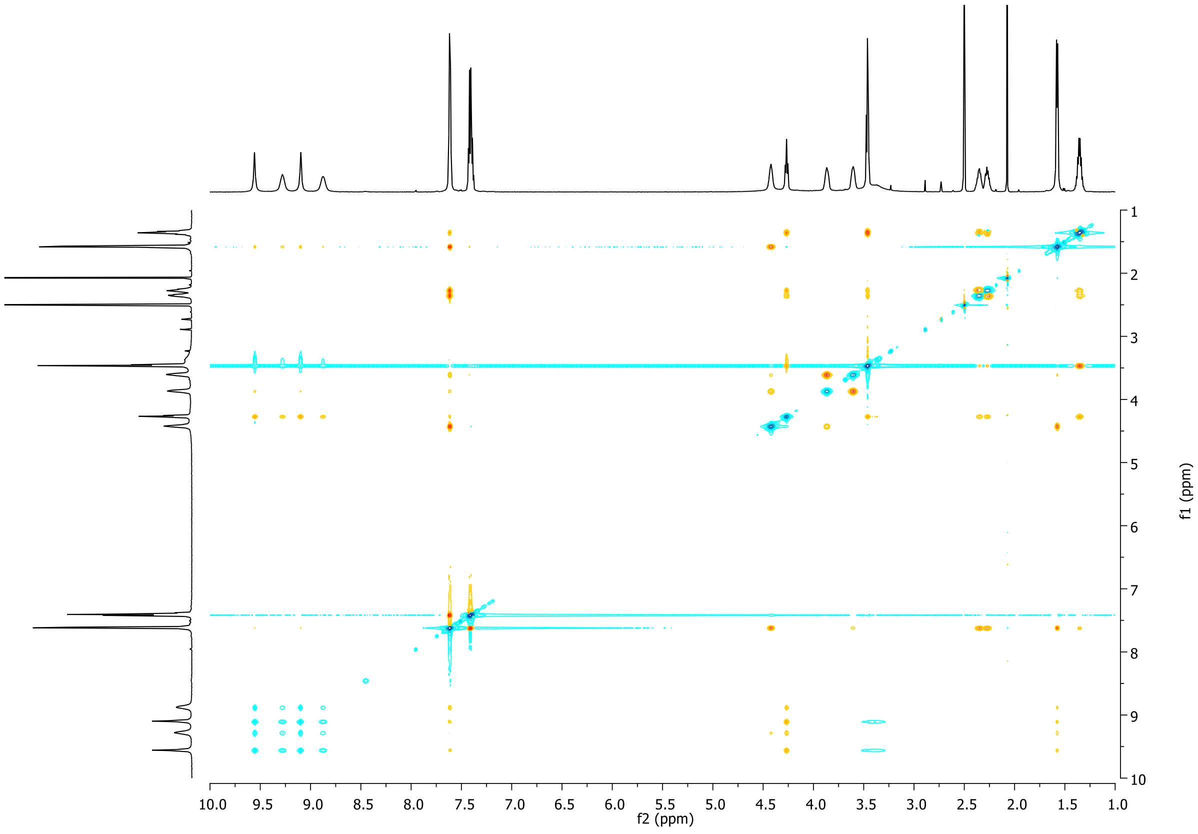

Figure S48. ROESY NMR (600 MHz, DMSO-d_6) spectrum of (*R*)-**2**.

1.4. NMR spectra of capsules

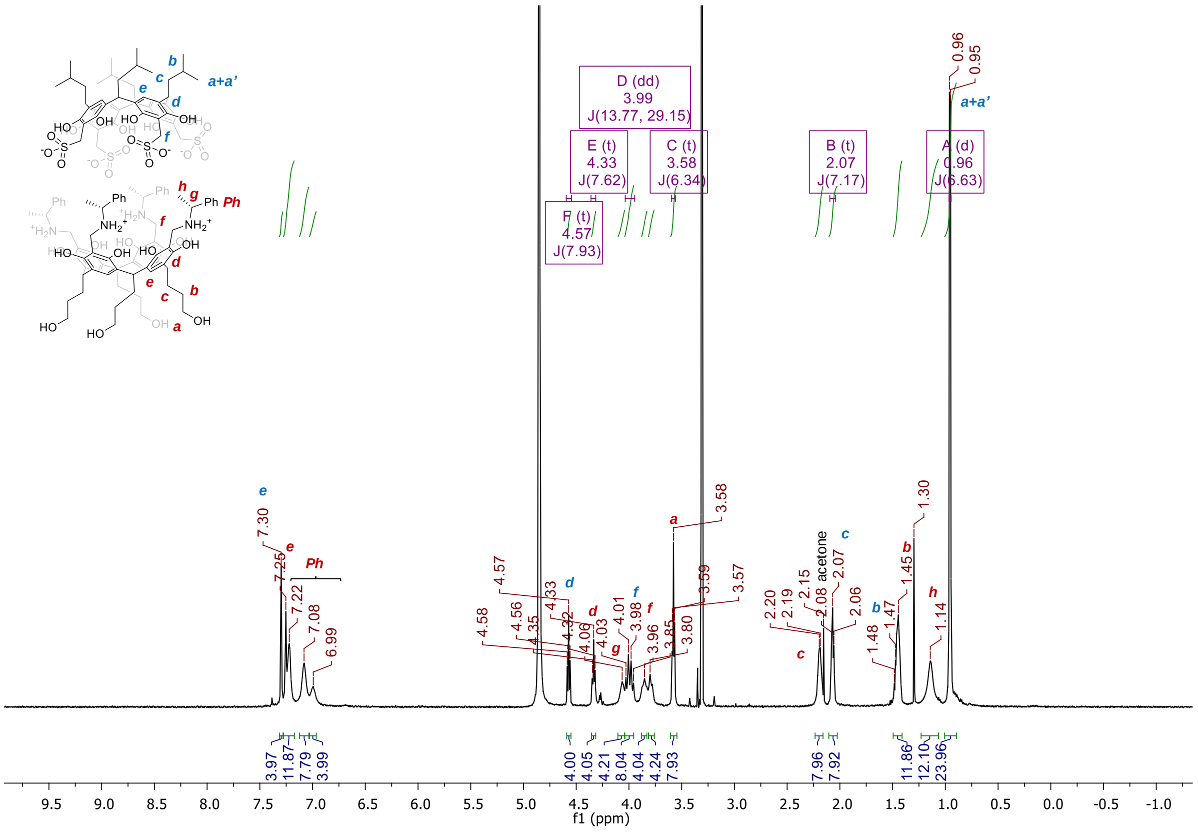

Figure S49. ^{1}H NMR (600 MHz, methanol-d_4) spectrum of capsule composed of **1** + (R)-**2**.

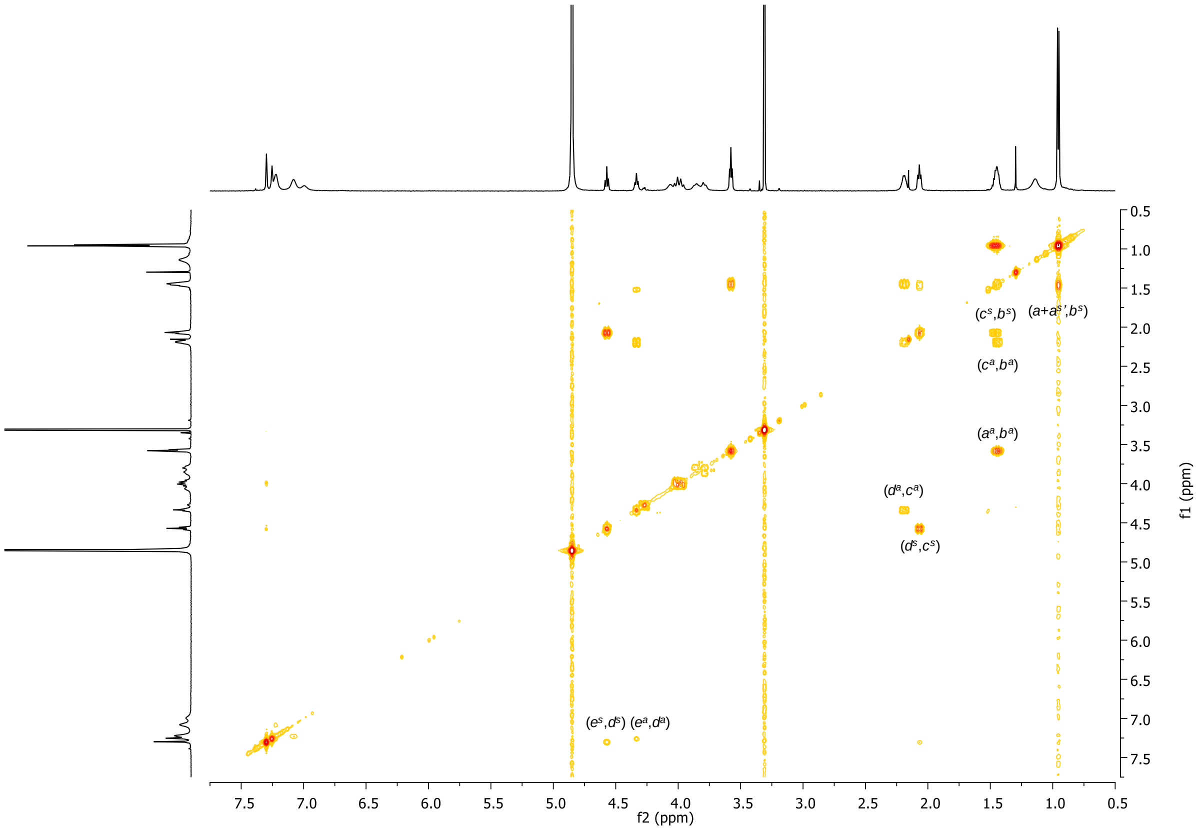

Figure S50. COSY NMR (600 MHz, methanol-d_4) spectrum of capsule composed of **1** + (R)-**2**.

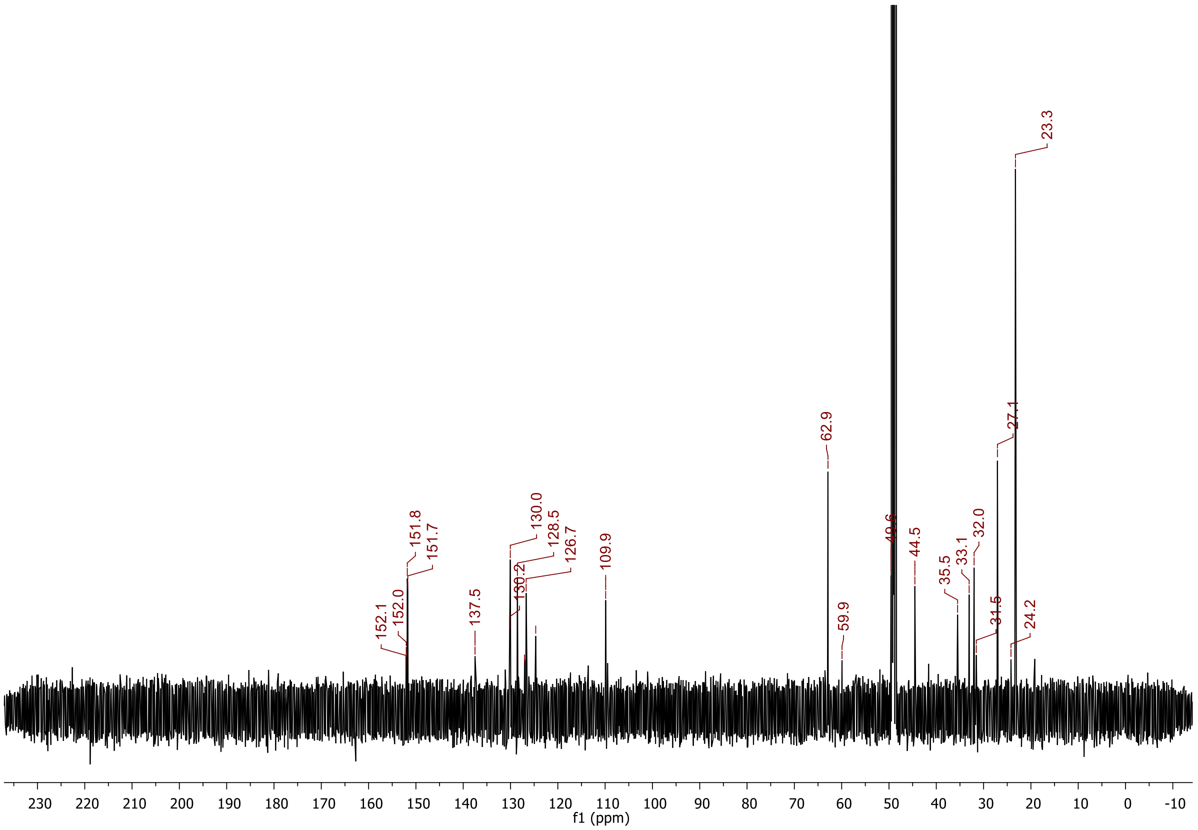

Figure S51. ^{13}C NMR (150 MHz, methanol-d_4) spectrum of capsule composed of **1** + (R)-**2**.

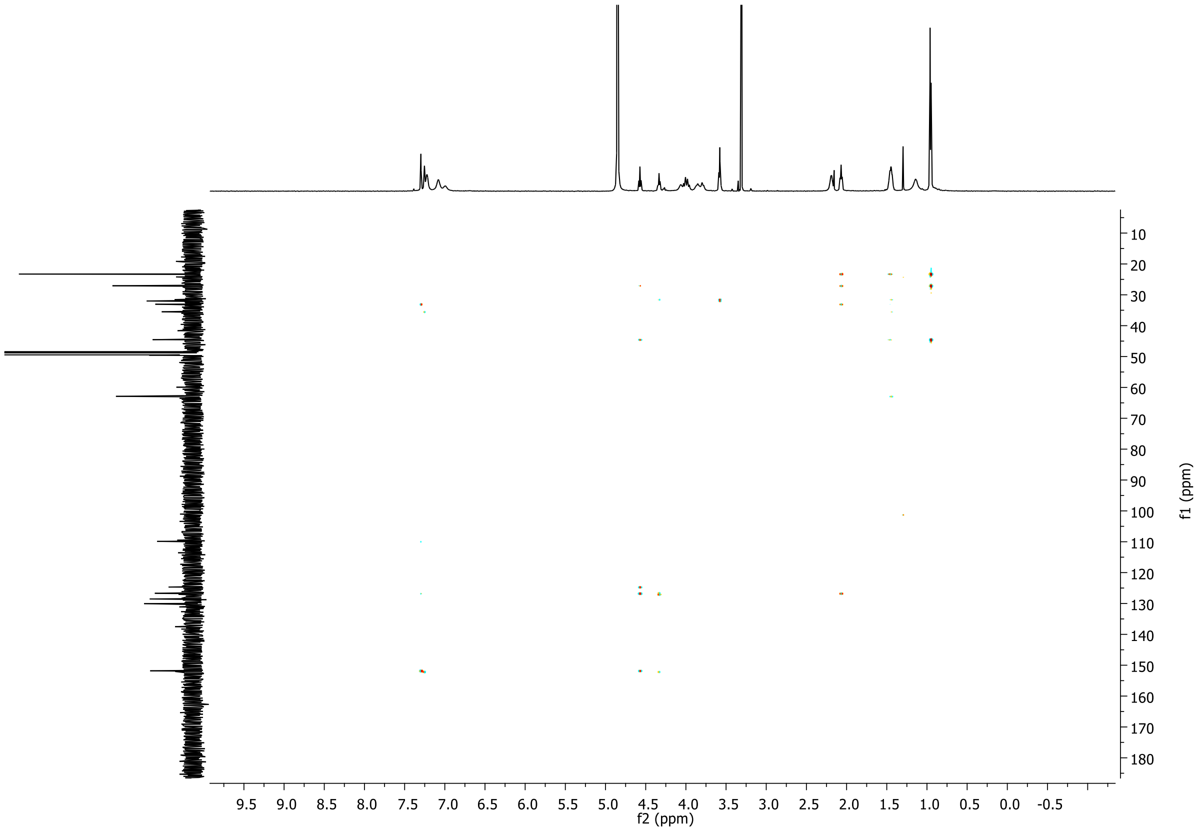

Figure S52. HMBC NMR (600 MHz, methanol-d_4) spectrum of capsule composed of **1** + (*R*)-**2**.

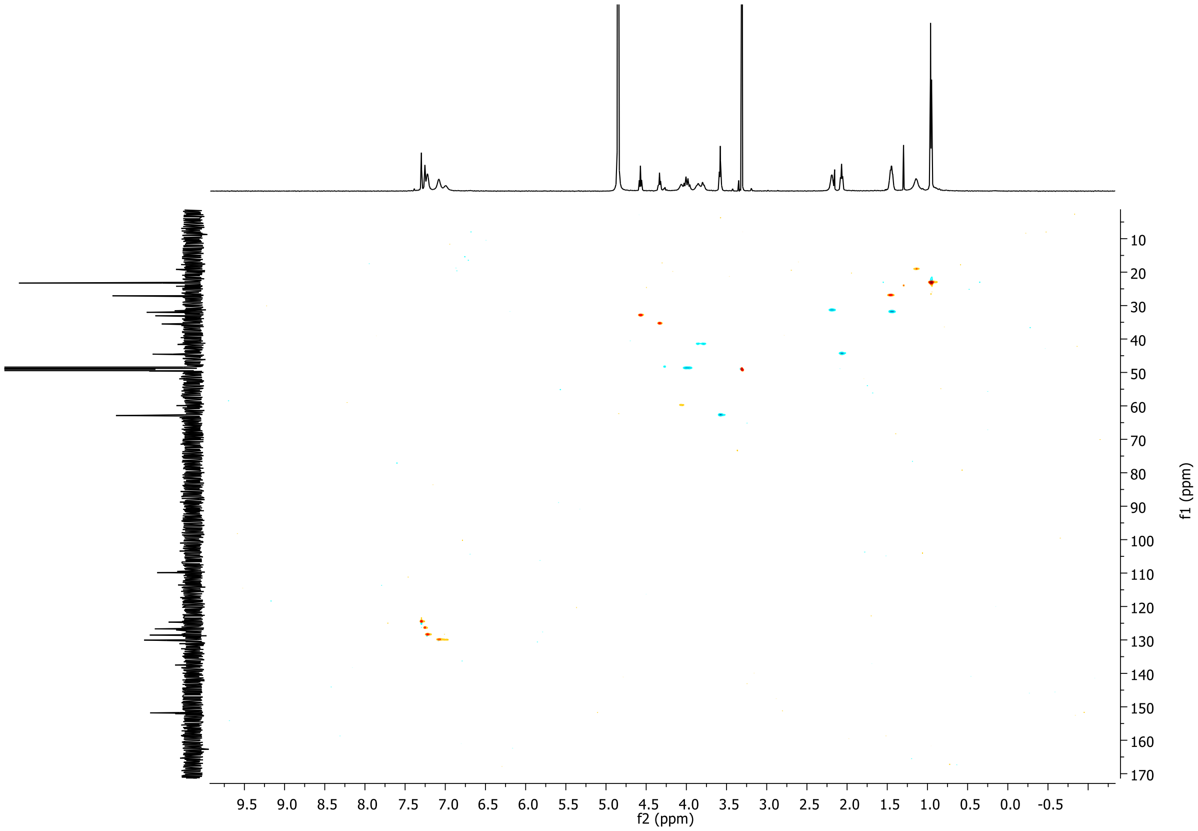

Figure S53. HSQC NMR (600 MHz, methanol-d_4) spectrum of capsule composed of **1** + (*R*)-**2**.

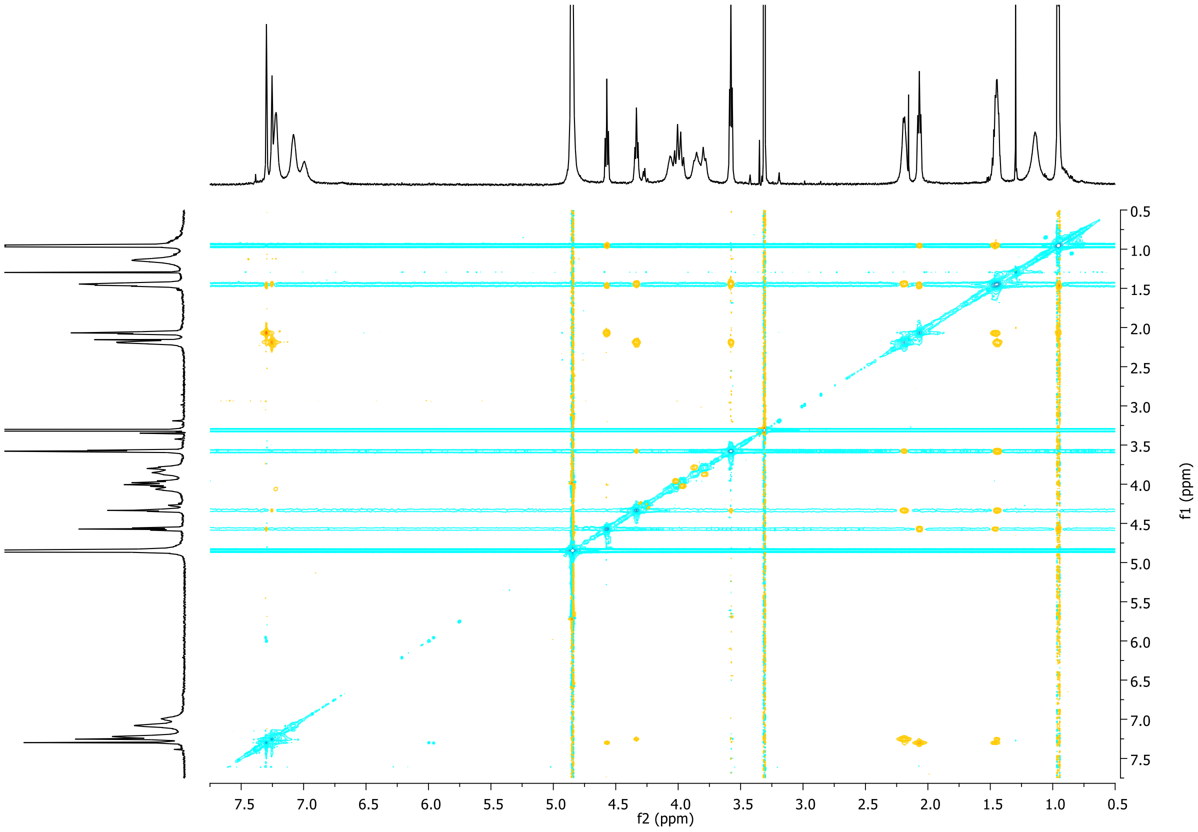

Figure S54. ROESY NMR (600 MHz, methanol-d_4) spectrum of capsule composed of **1** + (*R*)-**2**.

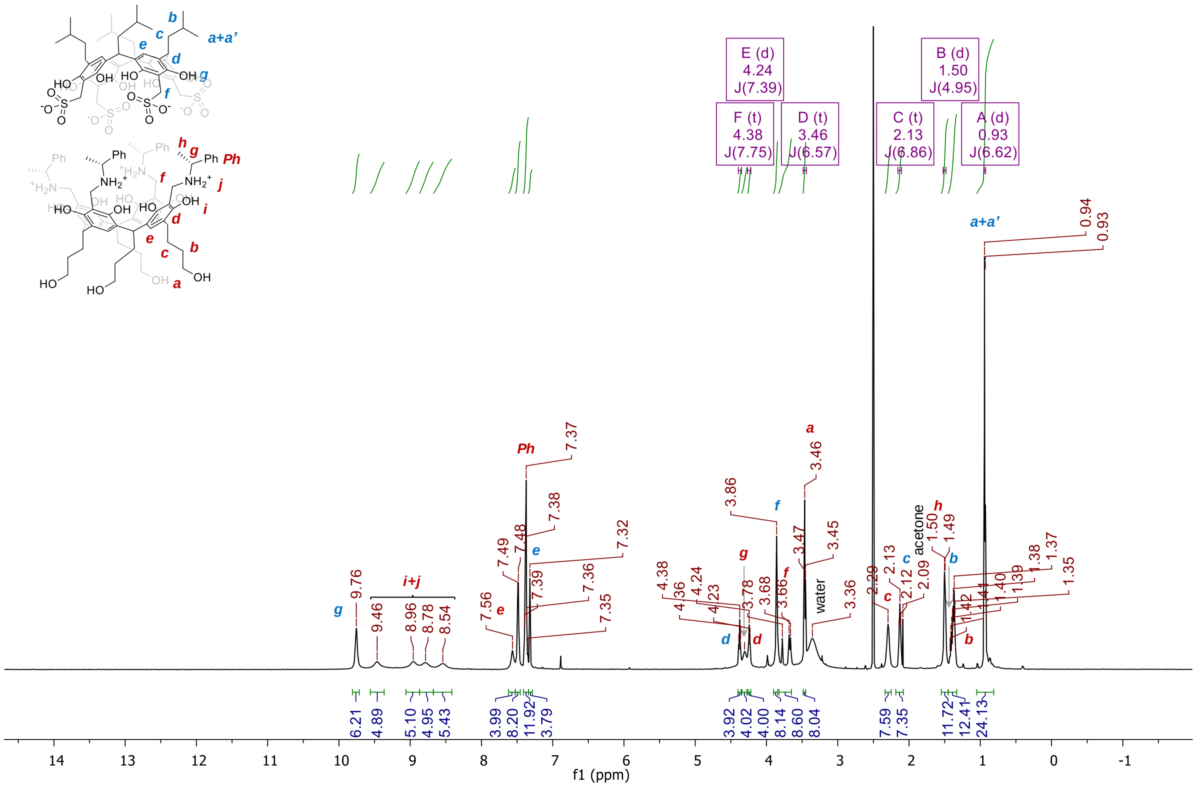

Figure S55. ^{1}H NMR (600 MHz, DMSO-d_6) spectrum of capsule composed of **1** + (*R*)-**2**.

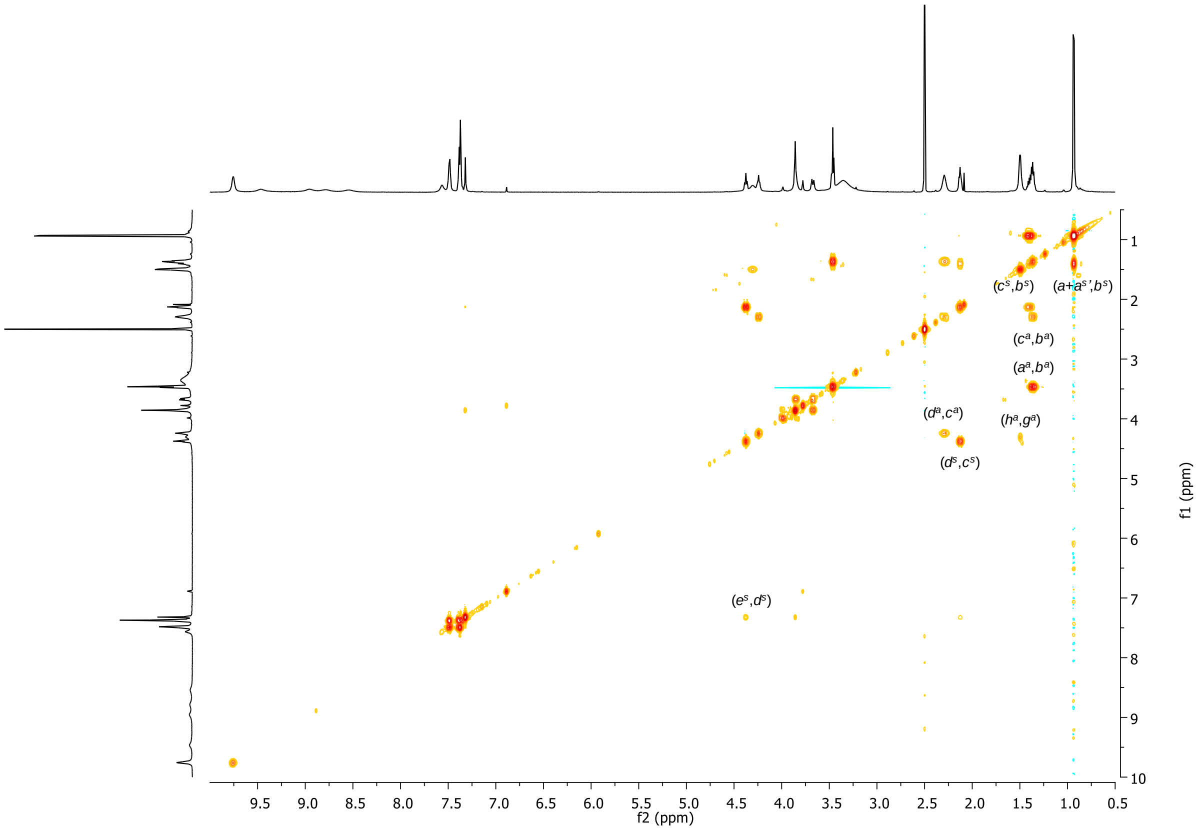

Figure S56. COSY NMR (600 MHz, DMSO-d_6) spectrum of capsule composed of **1** + (R)-**2**.

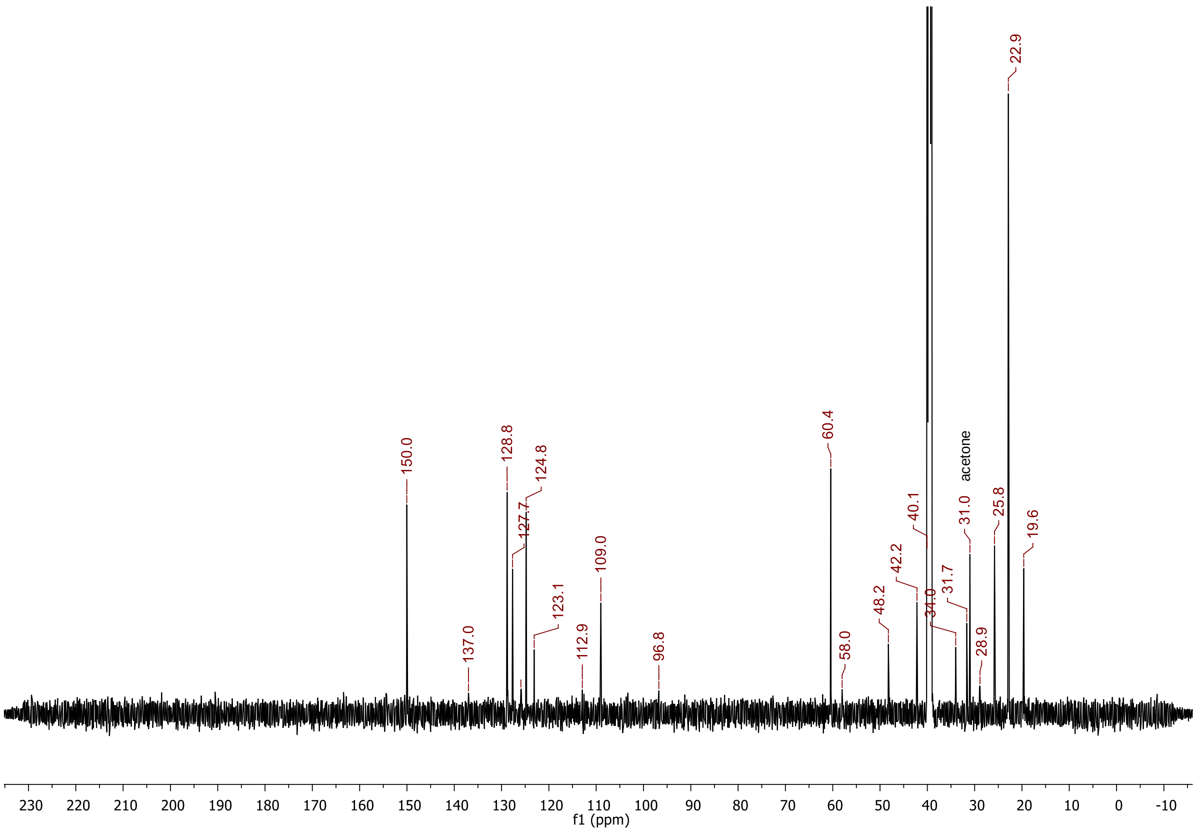

Figure S57. ^{13}C NMR (150 MHz, DMSO-d_6) spectrum of capsule composed of **1** + (R)-**2**.

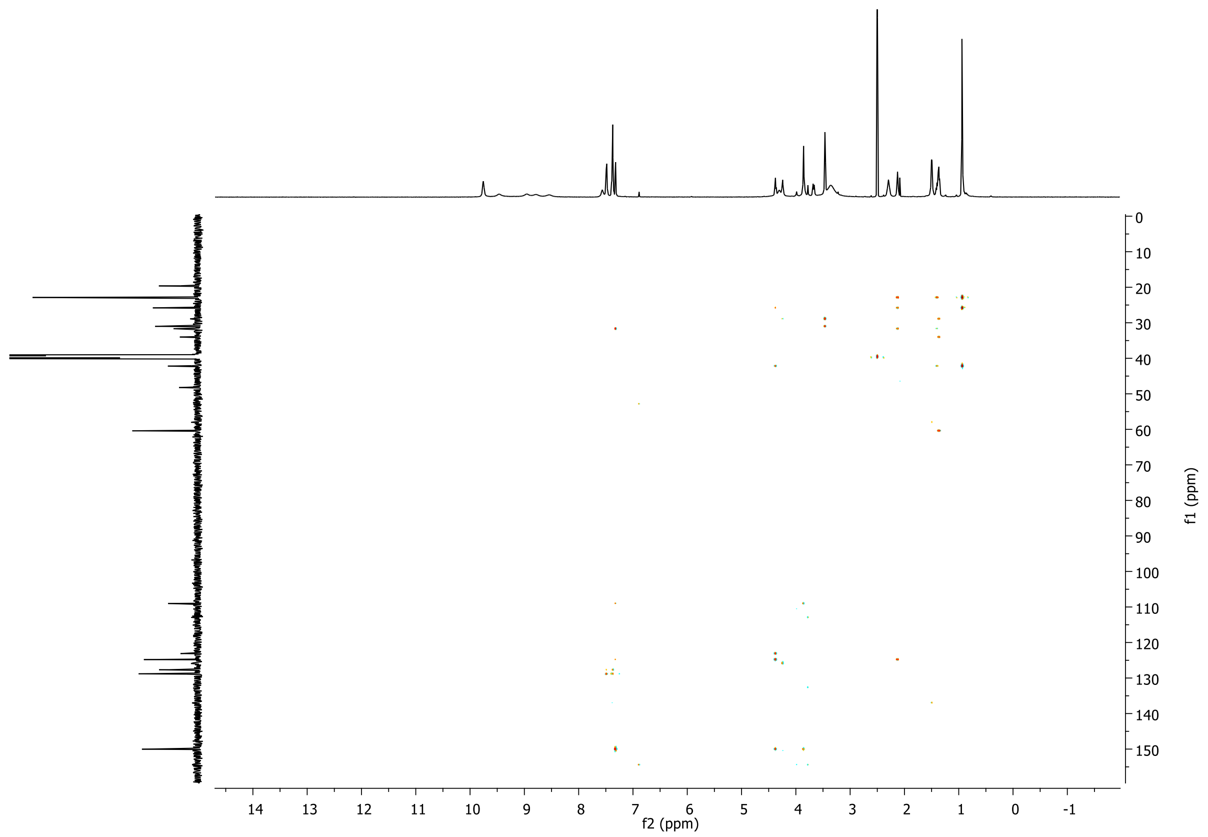

Figure S58. HMBC NMR (600 MHz, DMSO-d_6) spectrum of capsule composed of **1** + (*R*)-**2**.

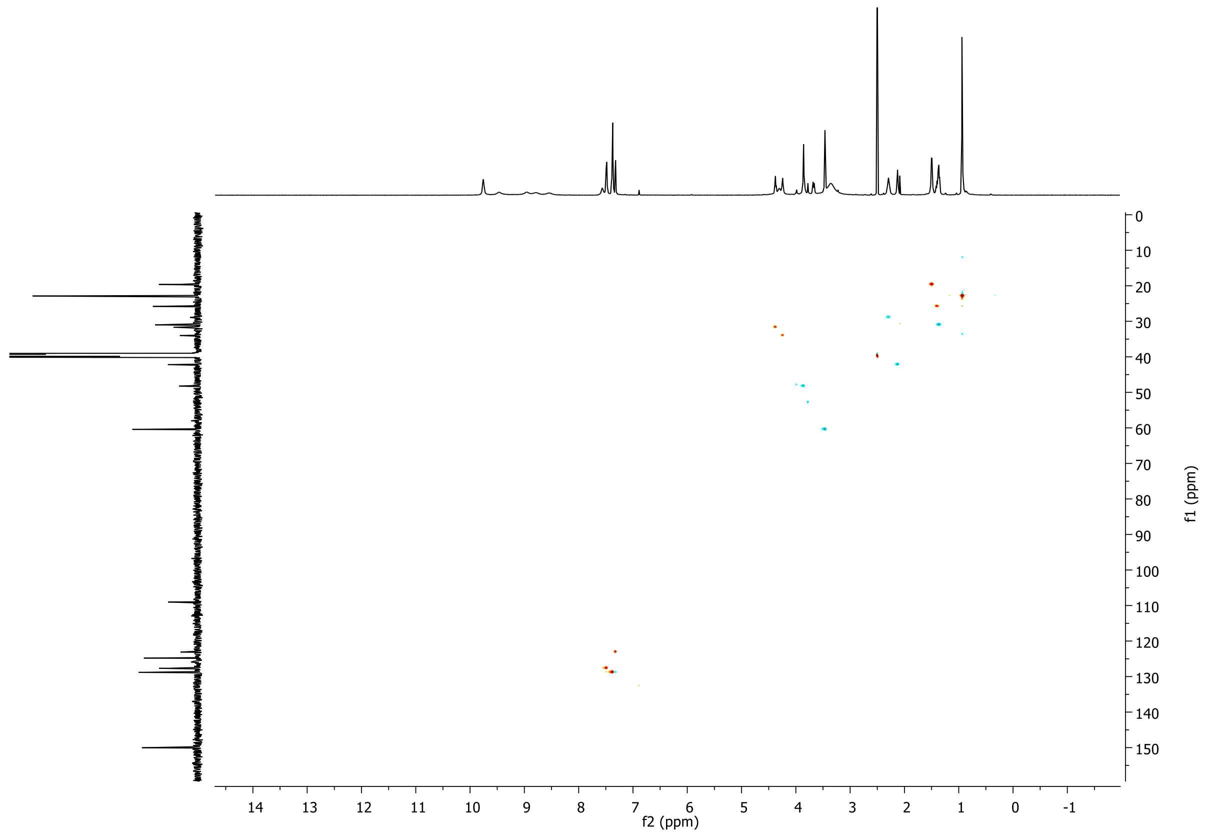

Figure S59. HSQC NMR (600 MHz, DMSO-d_6) spectrum of capsule composed of **1** + (*R*)-**2**.

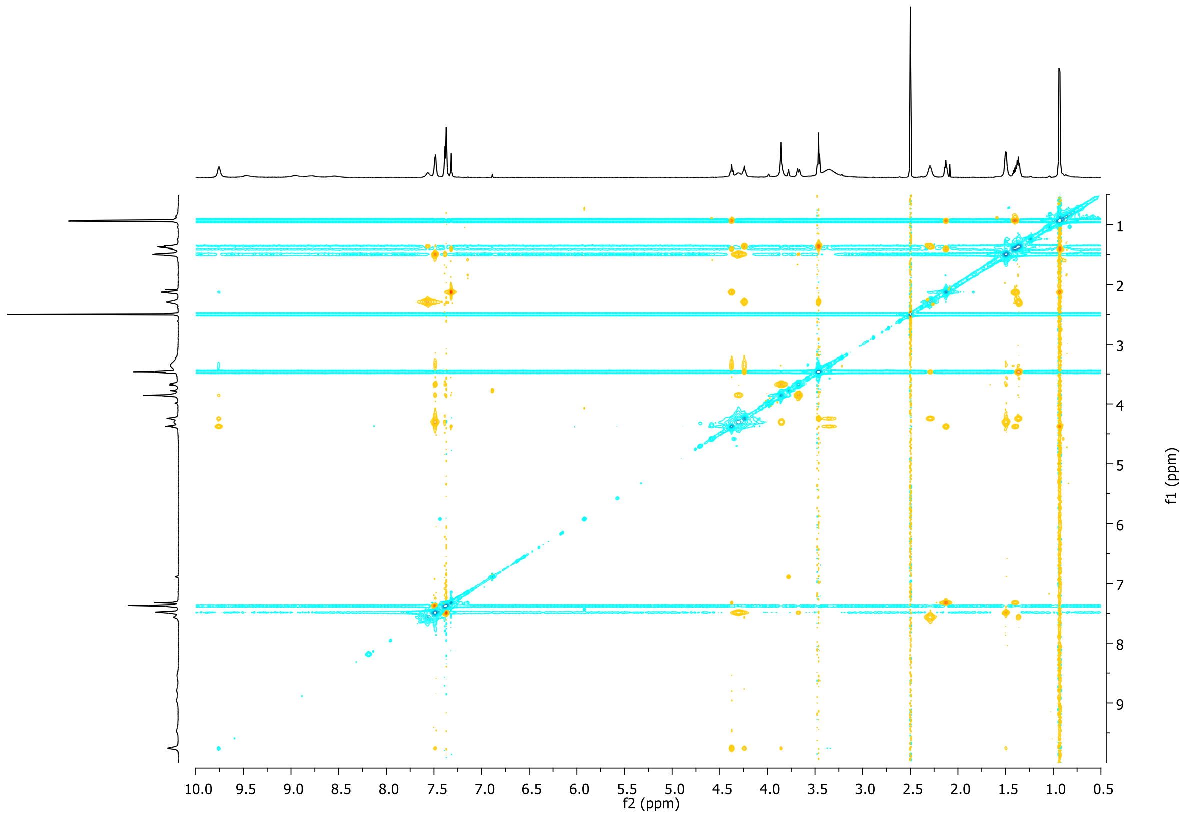

Figure S60. ROESY NMR (600 MHz, DMSO-d_6) spectrum of capsule composed of **1** + (*R*)-**2**.

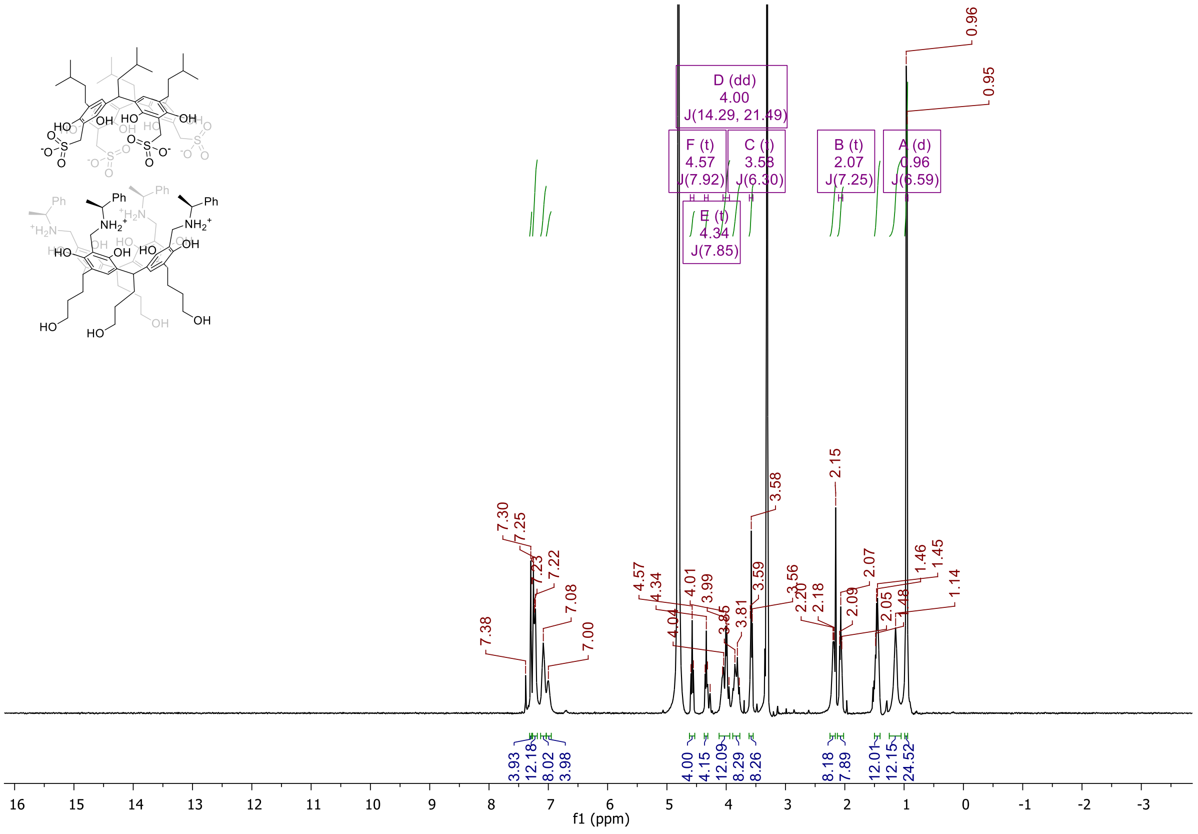

Figure S61. ^{1}H NMR (400 MHz, methanol-d_4) spectrum of capsule composed of **1** + (*S*)-**2**.

2. UV and ECD spectra

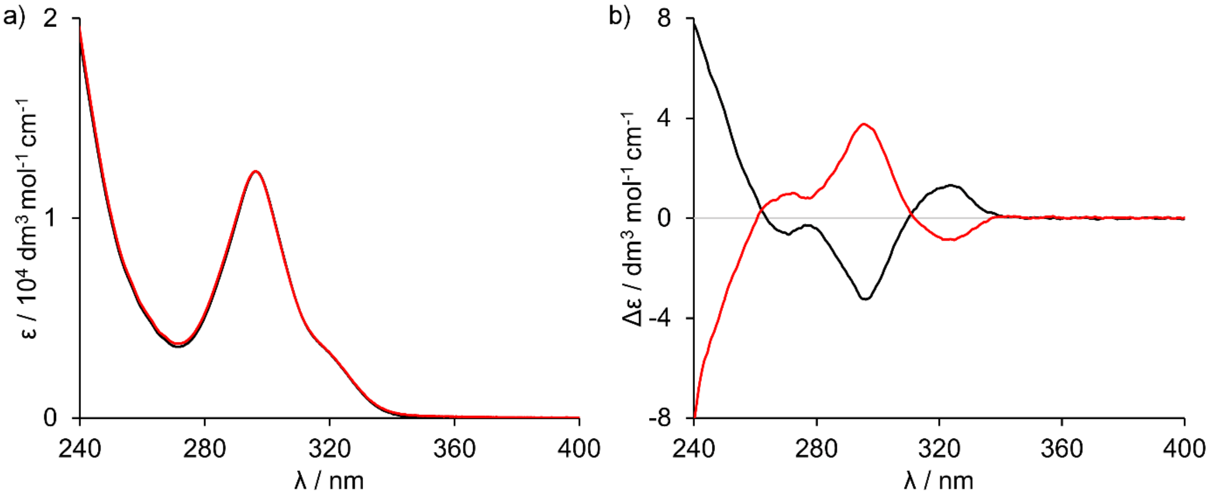

Figure S62. a) UV spectra of (*R*)-**2** (**black**) and (*S*)-**2** (**red**) in H_2O; b) ECD spectra of (*R*)-**2** (**black**) and (*S*)-**2** (**red**) in H_2O. The intensities of all spectra were normalized by using the UV band at 296 nm of sample (*R*)-**2**.

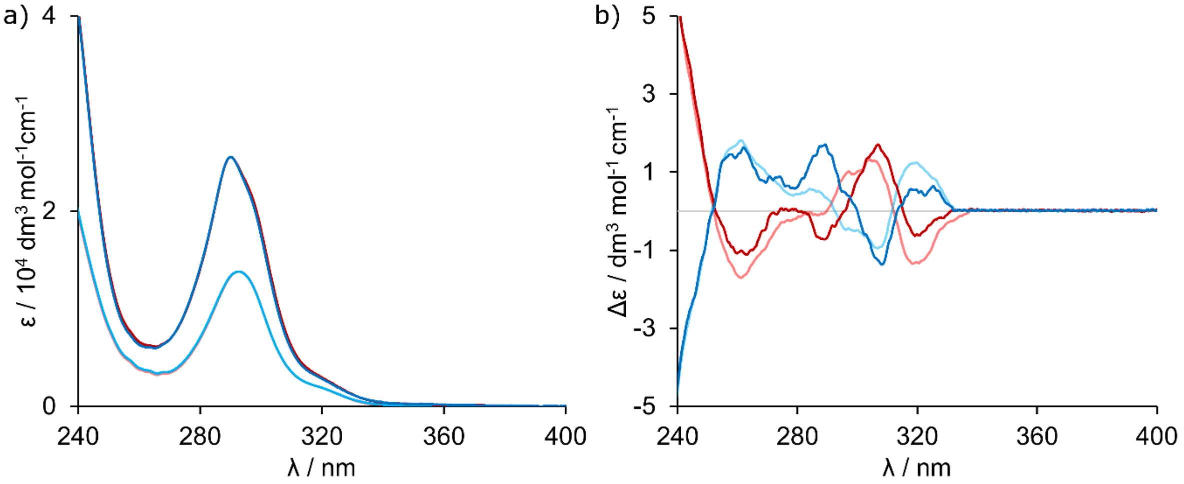

Figure S63. a) UV spectra of (*R*)-**2** (**light red**), (*S*)-**2** (**light blue**) and complexes **1** + (*R*)-**2** (**red**), **1** + (*S*)-**2** (**blue**) in CH_3OH; b) ECD spectra of (*R*)-**2** (**light red**), (*S*)-**2** (**light blue**) and complexes **1** + (*R*)-**2** (**red**), **1** + (*S*)-**2** (**blue**) in CH_3OH. The intensities of cavitand spectra were normalized by using the UV band at 290 nm of sample (*R*)-**2** whereas intensities of capsules spectra were normalized by using the UV band at 308 nm of sample **1** + (*R*)-**2**. The intensities of capsules spectra were multiplied by 2.

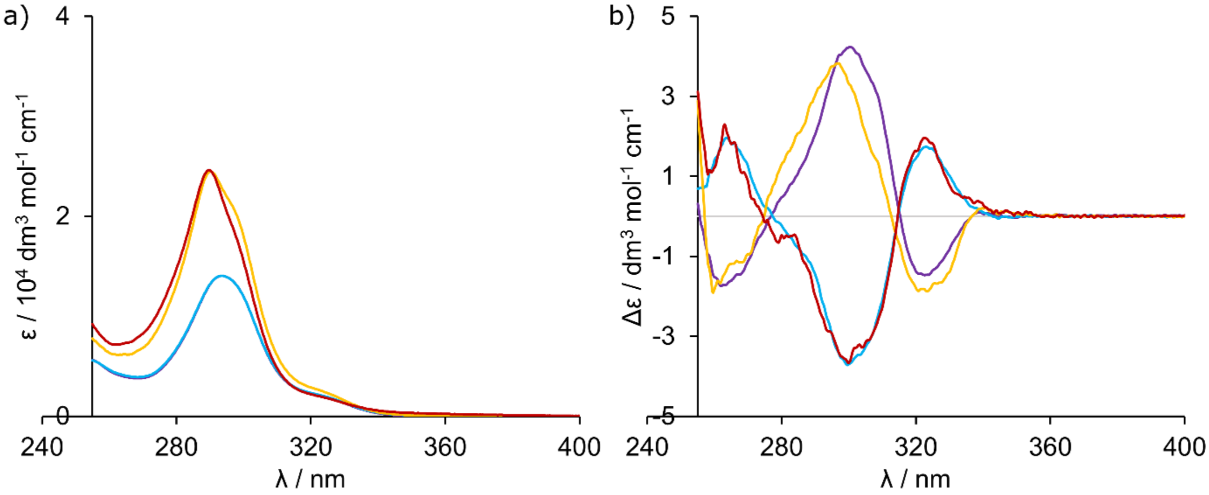

Figure S64. a) UV spectra of (*R*)-**2** (**violent**), (*S*)-**2** (**light blue**) and complexes **1** + (*R*)-**2** (**yellow**), **1** + (*S*)-**2** (**red**) in DMSO; b) ECD spectra of (*R*)-**2** (**violent**), (*S*)-**2** (**light blue**) and complexes **1** + (*R*)-**2** (**yellow**), **1** + (*S*)-**2** (**red**) in DMSO. The intensities of cavitand spectra were normalized by using the UV band at 293 nm of sample (*R*)-**2** whereas intensities of capsules spectra were normalized by using the UV band at 290 nm of sample **1** + (*R*)-**2**. The intensities of capsules spectra were multiplied by 2.

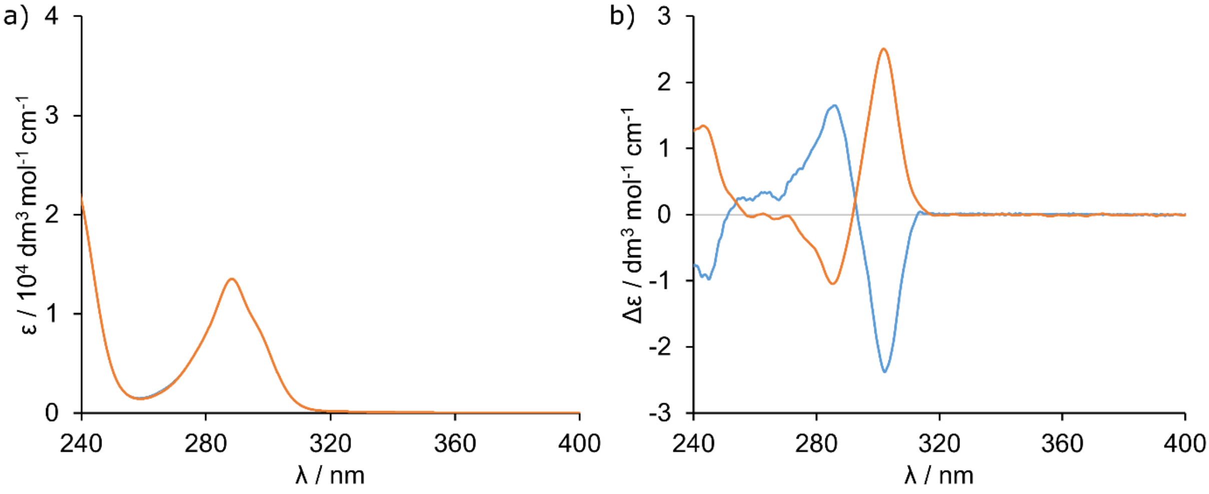

Figure S65. a) UV spectra of **1**(L–LysOMe)$_2$ (**orange**) and **1**(D–LysOMe)$_2$ (**blue**) in CH_3OH; b) ECD spectra of **1**(L–LysOMe)$_2$ (**orange**) and **1**(D–LysOMe)$_2$ (**blue**) in CH_3OH. The intensities of all spectra were normalized by using the UV band at 296 nm of sample **1**(L–LysOMe)$_2$.

3. ESIMS spectrum

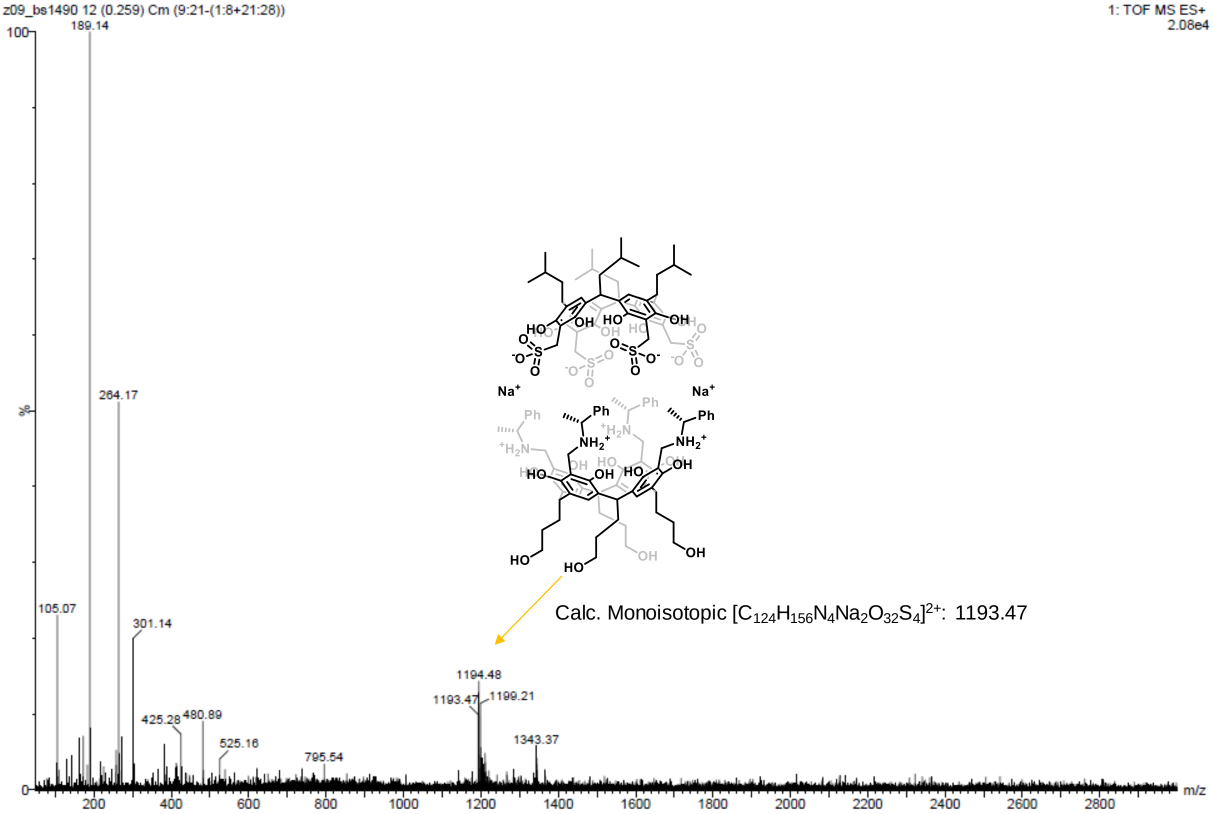

Figure 66S. ESIMS spectrum of **1** + (*R*)-**2**.
